# First-Principles Dynamics of Fluorine Adsorption on Clean and Monohydrogenated Si{001}

**DOI:** 10.1021/acs.langmuir.2c00740

**Published:** 2022-06-01

**Authors:** Ian Y. H. Wu, Stephen J. Jenkins

**Affiliations:** Yusuf Hamied Department of Chemistry, University of Cambridge, Lensfield Road, Cambridge CB2 1EW, United Kingdom

## Abstract

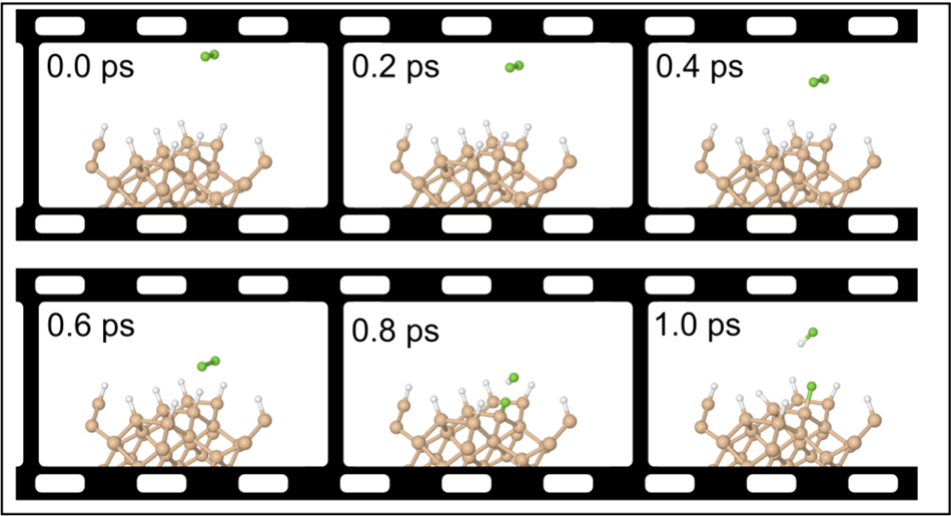

The interaction of highly reactive species with solid surfaces can result in modes of adsorption quite distinct from the classic molecular and dissociative events that are usually thought to dominate. For instance, compelling experimental evidence suggests that adsorption of F_2_ at the Si{001} surface is often initiated by abstraction (and binding at the surface) of just one fluorine atom from the molecule; the second fluorine atom subsequently experiences either a separate atomic adsorption event or ejection from the surface altogether. Molecular dynamics simulations using empirical potentials support this concept but massively overestimate the prevalence of atomic ejection. In this work, we report first-principles molecular dynamics calculations that correctly show atomic ejection to be rare while providing insight into the details of abstractive adsorption. In addition, we also examine the case of F_2_ adsorption onto a monohydrogenated Si{001} surface, finding evidence for a different type of abstractive adsorption, in which a hydrogen atom may be removed from the surface to form a short-lived HFF intermediate. The latter rapidly decomposes to produce either HF or (via reaction with another surface hydrogen atom) H_2_.

## I Introduction

Textbook treatments of molecular adsorption on solid surfaces tend to describe just two possible scenarios: one in which the molecule remains intact upon adsorption, and the other in which the molecule dissociates (partially or wholly) with all products becoming bound to the surface (however briefly). A third possibility does exist, however, albeit far less frequently encountered, in which only one fragment of a dissociating molecule becomes bound to the surface, with its complement departing promptly back into the medium from which it originated. An archetypal example of this third class of adsorption may be found in the interaction of molecular fluorine (F_2_) on the Si{001} surface, as predicted by the molecular dynamics simulations of Carter et al.^1−4^ and confirmed by the supersonic molecular beam experiments of Li et al.^5^ Here, in a substantial fraction of adsorption events, a single fluorine atom will be abstracted from the molecule to adsorb at the surface, with the remaining isolated fluorine atom ejected back into the space above. Precisely how frequently this occurs, in comparison with events where both atoms bind to the surface, is a matter of some discrepancy between theory and experiment. Nevertheless, an insightful discussion of the relevant kinetics was subsequently provided by Sholl,^6^ and a detailed kinetic model was proposed by Tate and co-workers.^7,8^

The matter is of more than purely academic importance, it should be said, having some passing relevance to the semiconductor industry. In one form or another, fluorine and fluorine-containing compounds are often used in the processing of silicon for device applications. For example, treatment with hydrofluoric acid (HF in aqueous solution) is used to remove layers of silicon oxide (SiO_2_), leaving behind a hydrogen-passivated silicon surface.^9^ On the other hand, treatment with atomic fluorine (in either plasma or gaseous state) can abstract hydrogen from a passivated surface^10^ and even etch into the silicon substrate itself via a process that liberates SiF_*x*_ (*x* = 2–4) species into the gas phase.^11−19^ Similar processes occur too on the surfaces of silicon compounds, such as SiO_2_, Si_3_N_4_, and SiC,^20,21^ and much insightful discussion of the wider field may be found in recent review articles by Kanarik et al.^22^ and by Rahman and Runyon.^23^

In contrast, molecular fluorine has not traditionally been widely employed for processing within the semiconductor industry, but exposure to gaseous F_2_ has been utilized in the context of basic research as a means to produce fluorinated surfaces for study purposes.^24,25^ It has also been noted that the molecular species will indeed etch silicon, albeit at a rather lower (room temperature) rate than does the atomic species.^26−33^ Moreover, in recent years, molecular fluorine (at elevated temperature) has been shown to be an extremely efficient alternative to greenhouse gases such as NF_3_ or SF_6_ for the purposes of cleaning silicon deposits from the walls of chemical vapor deposition (CVD) or atomic layer deposition (ALD) chambers.^34^

One unresolved discrepancy between experimental and computational studies of fluorine adsorption on Si{001} relates to the relative probability of the abstraction/ejection process (i.e., that which results in a single adsorbed fluorine atom and prompt desorption of its partner) versus the normal dissociative process (i.e., that resulting in two adsorbed fluorine atoms). Experiments suggest that abstraction/ejection accounts for only around 12% of adsorption events,^5^ while the simulations indicate well above 50% when modeling incoming molecules with comparable translational energies.^1−4^ In addition, the ejected fluorine atoms in the simulations are found to possess kinetic energies averaging in excess of 0.4 eV (velocities averaging in excess of 2000 m·s^–1^) while the experimental time-of-flight data yields an average kinetic energy of 0.126 eV (average velocity 1125 m·s^–1^) when working at a surface temperature of 250 K, rising to 0.139 eV (average velocity 1181 m·s^–1^) when working at a surface temperature of 1000 K. One possible explanation for this discrepancy may be the use of empirical potentials to describe the interatomic forces in the simulations. Notwithstanding the fact that parameters for such potentials may be fitted either to experimental data or to quantum chemical calculations, it is generally accepted that their accuracy tends to be questionable when bonds are either made or broken. Scope exists, therefore, to gain further insight by avoiding the necessity for fitted potentials altogether.

In the years since the original simulations discussed above, advances in computational power have rendered first-principles molecular dynamics feasible. In this approach, forces between atoms are calculated on-the-fly at each time step, using an appropriate first-principles method, such as density functional theory (DFT). It should be stressed that the computational resources required remain significant at the present time, and that compromise may be necessary over matters such as the size of simulation cell and/or the number of trajectories computed. The compensation for these compromises, however, is that one can have high confidence in the reasonableness of forces throughout the simulation, not to mention access to information concerning changes in the electronic structure from moment to moment as bonds are made and broken. Often, a so-called NVT ensemble is invoked, where the system temperature (*T*) is controlled by a thermostat, with both the system volume and particle numbers (*V* and *N*) held fixed (see, for instance, calculations of overlayer structure,^35^ surface stress,^36^ or interfacial diffusion^37^). This approach is suitable for describing the statistical mechanics of the system over time, but fails to capture the detail of individual reactive events, in which a thermostat would incorrectly dampen any significant exo- or endothermicity. For these situations, the NVE ensemble is most suitable, where the total system energy (*E*) is held fixed. Such methods have been increasingly employed to investigate the details of diverse reactive behaviors including, for instance, the role of vibrational excitation during molecular adsorption,^38−40^ the migration of molecules across a surface after electron excitation,^41−43^ and the induction of rotation in desorbing molecules.^44,45^

In previous work using the first-principles molecular dynamics approach, we examined reactions of ozone (O_3_) with the Si{001} surface, obtaining results somewhat reminiscent of the fluorine adsorption case. While some computed trajectories resulted in the complete dissociation of ozone into three oxygen adatoms, others resulted only in partial dissociation, culminating in adsorption of a single oxygen adatom and prompt ejection from the surface region of molecular oxygen (O_2_).^46,47^ Furthermore, in studies of ozone adsorption onto a hydrogen-passivated silicon surface, our calculated trajectories revealed an unusual radical-mediated mechanism, in which a hydrogen atom was first abstracted from the surface to form a vibrationally excited HO_3_ radical that subsequently dissociated to yield a surface-bound hydroxyl (−OH) species and gas-phase molecular oxygen.^48^ This, then, constitutes a fourth mode of adsorption, in addition to the intact, dissociative and abstractive modes discussed above. Inspired by these findings for ozone, we here report on simulations not only of F_2_ adsorption onto the clean Si{001} surface, but also onto a hydrogen-passivated version of the same.

## II Computational Method

Calculations were carried out using the CASTEP computer code, which implements first-principles density functional theory within periodic boundary conditions.^49^ The dimensions of the simulation cell were consistent with a *c*(4 × 4) unit cell in the surface-parallel plane, extending to a length equivalent to 16 layers of silicon in the [001] crystallographic direction. The surface itself was modeled with a slab of eight such layers, of which the back three layers were fixed at their bulk positions. The back surface of the slab was saturated with two hydrogen atoms per silicon atom, while the top surface was reconstructed by the formation of alternately buckled dimers and allowed to relax into its minimum energy geometry. Electronic wave functions were represented within a basis set of plane waves, up to a kinetic energy cutoff at 350 eV, and the Brillouin zone was sampled over a 3 × 3 × 1 Monkhorst–Pack mesh.^50^ The electron–ion interactions were included through the use of ultrasoft pseudopotentials,^51^ and the exchange-correlation interactions between electrons were included through the Perdew–Burke–Ernzerhof functional.^52^ The system was permitted to explore solutions having partial band occupancies and/or nonzero spin, both of which situations may arise while bonds are made or broken.

The dynamic simulations were performed within the NVE ensemble using a time-step of 0.5 fs and initialized with the incoming molecule approaching the surface along the surface normal at a speed of 362 m·s^–1^ (kinetic energy 0.026 eV). This corresponds to the most probable speed for gaseous F_2_ at 300 K, according to the kinetic theory of gases, and is tolerably close to the speed of 390 m·s^–1^ (kinetic energy 0.030 eV) used in the supersonic beam experiments reported by Li et al.^5^ The silicon atoms were all initialized with zero velocity, corresponding to a surface temperature of 0 K, which is at odds with surface temperatures in the range 250–1000 K described in the experimental work. We note, however, that the experimental results are rather insensitive to surface temperature and that the lower end of the investigated temperature range corresponds to a *k*_B_*T* value of only about 0.02 eV. Each Si dimer would therefore possess around 0.06 eV of kinetic energy, if we were to thermally populate its vibrational modes according to an ergodic assumption. By way of comparison, dissociation of F_2_ in the course of trajectories calculated in this work typically results in highly localized liberation of at least 4 eV, of kinetic energy, entirely swamping any thermal energy that we omit from our substrate model.

**Figure 1:**
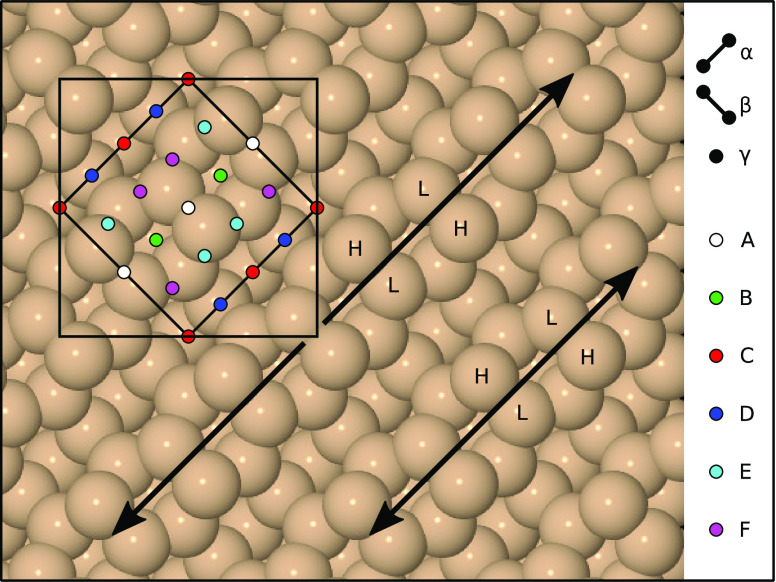
Schematic top-down view of the clean Si{001} surface, highlighting four individual dimers (with H and L indicating high- and low-lying dimer atoms) and two dimer rows (with double-headed arrows running along adjacent examples). The surface exhibits a (2 × 2) reconstruction, whose primitive cell is marked with the smaller square, but calculations were performed within a *c*(4 × 4) unit cell, marked with the larger square. Incoming molecules were aimed at sites A–F, shown here in multiple instances to emphasize the uniformity of their distribution. Note that the surface displays 2-fold rotational symmetry about sites B and D, glide symmetry along the dimer rows, and mirror symmetry across them, reducing the number of symmetrically distinct sites that must be considered. At the monohydrogenated surface, each dimer atom is decorated with a single hydrogen atom and the dimer tilt is removed, causing sites E and F to become equivalent. Molecular axis orientations are indicated by bars labeled α (along the dimer rows) and β (across the dimer rows). A third orientation, γ, has the molecular axis perpendicular to the surface.

In each trajectory, the molecule was aimed at one of six nominal target sites (A–F) that span the surface unit cell in a rather uniform fashion (see Figure 1). In the case of the hydrogen-passivated surface, where the tilt of the silicon dimers is nullified, symmetry relates two of these sites (E and F) so that only one must be explicitly calculated. For each target site, simulations were initialized with the molecule aligned with its axis pointing along the dimer row, across the dimer row, or vertically, and we label these orientations α, β, and γ, respectively. We therefore calculate 18 distinct trajectories on the clean surface, and 15 on the passivated surface.

When describing in detail the system dynamics for a particular trajectory, it will be necessary to reduce a panoply of continually varying geometric parameters to a small number of discrete events that can be captured in words. For instance, a given atom may move closer to one atom or further from others, and while some such changes prove to be critical turning points in the fate of that atom, others amount to little more than footnotes in its wider history. By way of injecting some rigor into our analysis, we shall adopt a few conventions to aid in sorting the wheat from the chaff. Note, however, that these are based on purely geometric considerations, for reasons of simplicity; bonding signatures based on calculated forces or orbital occupancies might permit a more compelling analysis, but would be prohibitively costly to evaluate at every time-step. First, we draw the reader’s attention to a set of equilibrium bond lengths for all combinations of species that arise in the present work (Table 1). When two atoms approach each other at distances below the relevant equilibrium bond length, it is reasonable to consider that they experience a repulsive interaction. If a given pair of atoms should happen to pass into this repulsive regime for the first time, we shall describe the moment of closest approach as an impact or collision. If they should then pass repeatedly into the repulsive regime, we shall describe their motion as an oscillation or vibration, and consider that a bond has formed. Identification of bond breaking is more subjective, but we take note both of the duration and extremity of periods spent beyond the equilibrium bond length in our determination of when such an event may have occurred. We estimate vibrational frequencies for extant bonds directly from the periodic spacing of minima in the interatomic distance, quoting results only to the nearest multiple of 5 cm^–1^ when fewer than 10 cycles are available. Since this approach naturally incorporates anharmonic effects, we do not apply any scaling factor to these estimates.

**Table 1:** Equilibrium Bond Lengths Used as a Benchmark for Identifying Collisions between Atom Pairs^a^

bond	notes	length (Å)
H–H	calculated gas phase	0.76
H–F	calculated gas phase	0.95
H–Si	H attached to Si dimer	1.48
F–F	calculated gas phase	1.42
F–Si	F attached to Si dimer	1.60
Si–Si	experimental bulk value	2.35

a Apart from the bulk silicon bond length (taken from experiment) all others have been calculated by us (with convergence parameters identical to our molecular dynamics calculations) for either gas-phase molecules or for adsorbed adatoms relaxed in the stated geometries at 0.125 ML coverage.

To assist in analyzing electronic structure, we keep track of two different spin measures as each trajectory proceeds. The first, which we shall describe as the integrated net spin, is defined as
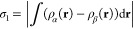
1where ρ_α_(**r**) and ρ_α_(**r**) are the spin densities of the two spin species accounted for in our calculations and where the integral spans the full volume of the supercell. Since it is essentially arbitrary which spin species happens to be globally dominant, we take the modulus of the integral with no loss of generality. The second measure, which we shall describe as the integrated spin modulus, is defined as
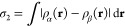
2in which the modulus of the integrand is taken, rather than that of the integral. The distinction is that the integrated net spin, σ_1_, will only be nonzero when the system possesses a well-defined majority spin species whereas the integrated spin modulus will be nonzero whenever the system possesses regions of local spin imbalance, even if multiple such regions counterbalance one another perfectly when considered together. For an idealized system in which spins are well-localized on atomic sites, therefore, the integrated spin modulus will reveal the total amount of localized spin while the integrated net spin will indicate whether the regions of localized spin are aligned parallel or antiparallel with one another. Evidently the interpretation will be less straightforward when spin is rather more delocalized, but nevertheless these two measures carry complementary information and reward close study. Very often, we shall find that these two measures take similar values (indicating largely parallel spins) but substantial differences can occasionally arise (indicating somewhat antiparallel spins) and will be noted as and when they occur.

## III Adsorption on the Clean Surface

The first glaring result from our study of F_2_ adsorption on the clean Si{001} surface is that none of the 18 trajectories considered results in the ejection of any species from the surface. On the basis of experiment, we ought to have expected perhaps one or two ejection events, given the estimate from Li et al.^5^ that ca. 12% of F_2_ adsorption events resulted in ejection of a single F atom from an initially clean surface. It is, of course, true that our simulated trajectories are relatively few in number, but they have been chosen systematically and ought to be reasonably representative of a random ensemble. In fact, not only do we fail to observe any ejection events, but none of our simulated trajectories appear even close to displaying this behavior, let alone with the high ejection velocities inferred from experiment. This apparent failure to match the experimental results stands in contrast to earlier computational simulations, where ejection was (at least equally erroneously, we must point out) observed in around 50% of adsorption events.^1−4^ Those earlier calculations, however, were based on the use of empirically derived potentials, and the current simulation ought therefore to be viewed as inherently more reliable. The potential energy surface explored in the present work derives from an accurate quantum mechanical calculation performed on-the-fly at each step in the simulation, whatever the arrangement of atoms happens to be at that particular moment. Empirical potentials, on the other hand, are typically fitted to reproduce structural, energetic, and vibrational properties only for intact molecules; extrapolation to predict the forces that act while bonds are either made or broken cannot, therefore, be considered entirely reliable, since the training data does not include similar scenarios. Indeed, as noted in our introductory remarks above, both the frequency of ejection events and the velocity of ejected atoms are considerably overestimated by the empirical technique. The initial conditions necessary to induce an atom-ejection event are discussed in some depth below, after our analysis of nonejection trajectories and their final geometries.

### III.A Categorization of Final Geometries

While the detailed motion of atoms differs in the course of each currently simulated trajectory, it is nevertheless possible to categorize them quite meaningfully according to the final state attained by the system. Although the atoms do not, of course, ever cease moving, in all cases the system eventually achieves a situation in which motion is purely vibrational, with all translational and rotational degrees of freedom quenched. On reaching such a state, the system exhibits one of just four final conditions: (i) two adatoms attach to separate dimer atoms; (ii) one adatom attaches to a dimer atom, the other to a second-layer silicon atom; (iii) two adatoms attach to separate second-layer silicon atoms; or (iv) two adatoms attach to a single dimer atom, the dimer itself being cleaved. Across our 18 simulated trajectories, these outcomes occur in the ratio 10:5:2:1 (see Table 2).

**Table 2:** Summary of Outcomes from Trajectories in Which F_2_ Was Aimed at Six Different Sites of the Clean Surface (A–F) in Three Different Orientations (α–γ) as Defined in Figure 1, Grouped According to the New Features Created at the Surface upon Adsorption^a^

new features	label	trajectory
2×(Si–F)_1_	i(a)	B/γ, C/γ
	i(b)	B/α
	i(c)	A/β, B/β, F/γ
	i(d)	A/α
	i(e)	D/β, E/α, F/α
1×(Si–F)_1_ &	ii	C/β, D/γ, E/β, E/γ, F/β
1×(Si–F)_2_ &		
1×(Si – )_3_		
2×(Si–F)_2_ &	iii	C/α, D/α
2×(Si−)_3_		
1×(F–Si–F)_1_ &	iv	A/γ
1×(Si=)_1_		

a The notation (Si–F) indicates a single fluorine atom bound to a silicon atom, while (F–Si–F) indicates two fluorine atoms bound separately to a single silicon atom, (Si−) indicates a silicon atom that gains a dangling bond where none were originally present, and (Si=) indicates a silicon atom that gains a second dangling bond where only one was originally present. In each case, a subscript indicates the surface layer in which the relevant silicon atom resides, taking the uppermost layer to be the first. Roman numerals label the four types of behavior described in the text, subdivided by lowercase letters as appropriate.

Among the commonest of these four scenarios, where the adatoms attach to two separate dimer atoms, it is interesting to further subdivide the outcomes according to which dimers are involved. In two cases, which we shall label i(a), both adatoms attach to a single dimer, while in another, i(b), they attach to two dimers that neighbor each other within a single dimer row, with these dimers being just 3.83 Å apart. In three further cases, i(c), the adatoms attach to dimers that neighbor each other in adjacent dimer rows, and in another, i(d), the involved dimers are next-nearest neighbors within a single dimer row. Both of these subcategories of adsorption involve dimers that are 7.67 Å apart. Finally, we note three examples, i(e), where adatoms attach to dimers that lie diagonally across from one another (at a separation of 8.57 Å) in adjacent dimer rows.

We have not explicitly subdivided the scenarios in which adatoms attach to second-layer silicon atoms, labeled ii and iii, but note that there is nevertheless some degree of variation among the detailed outcomes. These scenarios do, however, share the important common feature that a dangling bond is created at one of the third-layer silicon atoms, identified in all such cases by a dramatic stretching (interpreted as cleavage) of one of the Si–Si bonds between the second and third layers. In the single observed case where both adatoms are attached to the same silicon atom, labeled iv, the involved dimer actually breaks apart, leaving the second dimer atom with two dangling bonds rather than one. Taken as a whole, we see that adsorption of F_2_ typically features some combination of dimer cleavage, dangling bond creation, and/or the wide spatial separation of adatoms.

### III.B Descriptive Dynamics

While it would be excessive to describe in detail the dynamics of all our calculated trajectories, it will nevertheless prove instructive to look closely at a few representative examples (see the Associated Content statement for availability of data from all trajectories). In each case, we shall examine the evolution of the system’s spin characteristics alongside selected interatomic separations, with a view to understanding the making and breaking of bonds as the process of adsorption proceeds.

#### III.B.1 B/γ Trajectory

**Figure 2:**
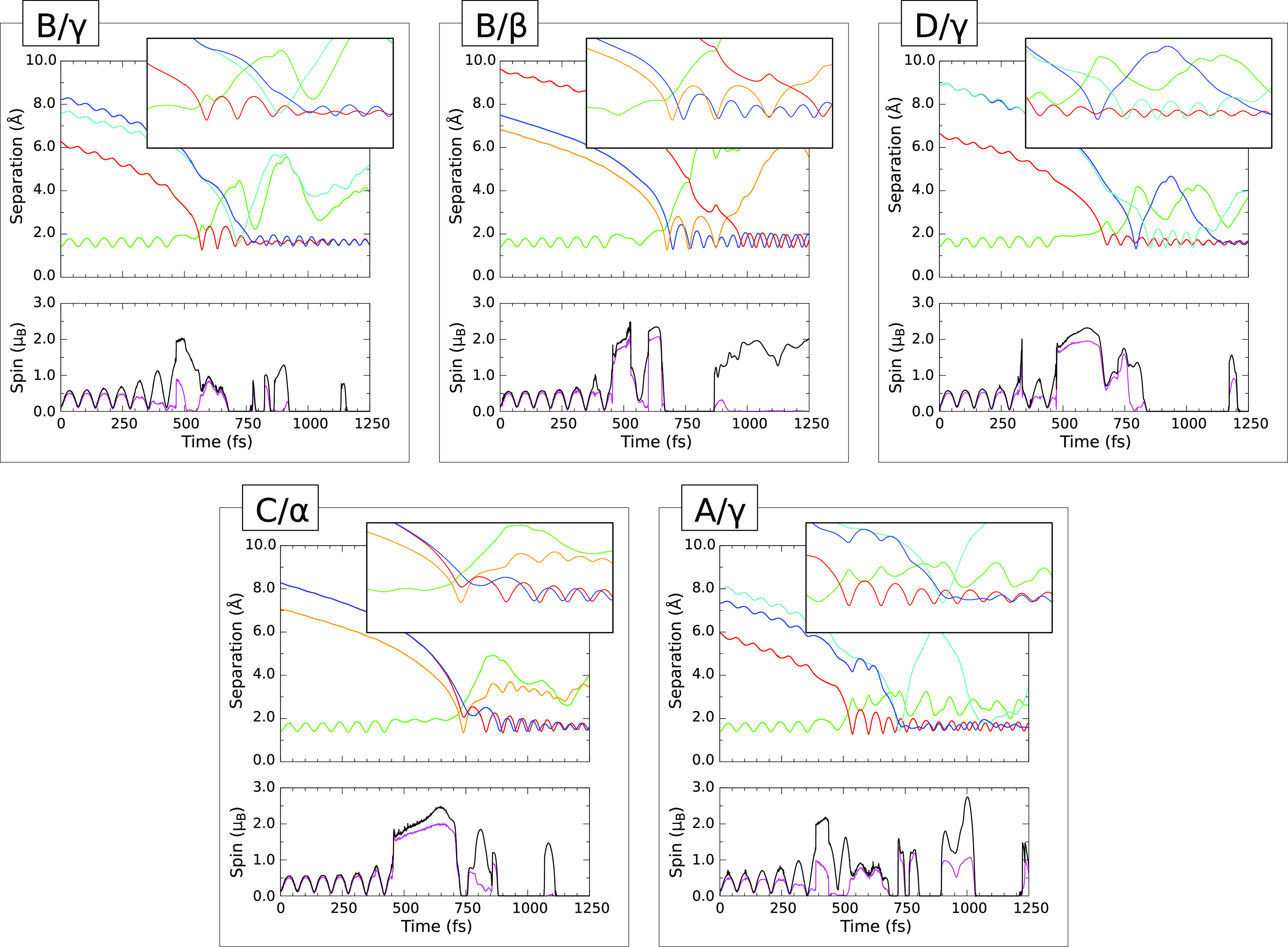
Evolution of selected trajectories on the clean surface. In each case, the upper panel shows interatomic separations. The F–F distance is shown always in green; Si–F distances involving the prompt fluorine atom in red or orange; and Si–F distances involving the tardy fluorine atom in blue or cyan (see text for details). Expanded insets cover a temporal range of 500 fs and the spatial range 0–5 Å. Lower panels for each trajectory show the integrated net spin in magenta and the integrated spin modulus in black.

Let us begin with a trajectory that leads to a final geometry of the type labeled i(a) in Table 2 specifically the B/γ case, whose progress is displayed in Figure 2B/γ. Here, in the upper panel, we indicate the F–F separation with a green line, as we shall do throughout all the graphs of this type discussed below. Regarding Si–F separations, however, there are a great many such traces that could conceivably be displayed, among which we must make some sensible choice. We opt to show, for each of the two fluorine atoms, the variation in distance from the silicon atom to which it will eventually bond (the red and blue curves). Finally, we note that one of the two fluorine atoms (the one corresponding to the blue trace) first interacts strongly with an entirely different silicon atom prior to connecting with its final bonding partner, and so we include one additional curve to show the interatomic separation relevant to this interaction too (the cyan curve). In the lower panel, we show the variation of integrated net spin (eq 1) and integrated spin modulus (eq 2) with black and magenta traces respectively.

Inspection of Figure 2B/γ allows us to follow the progress of adsorption rather closely, commencing with the initial approach of the molecule over the first 450 fs of the simulation. Interaction with the surface is already in evidence from the start, inducing a modest vibration in the molecule at a frequency of about 480 cm^–1^. This is substantially red-shifted (by 29%) relative to the fundamental vibrational frequency of 894 cm^–1^ measured for F_2_ by means of matrix-isolation Raman spectroscopy but remarkably similar to that of 475 cm^–1^ obtained for the F_2_^–^ anion in the same experiment.^53^ At this stage, the integrated net spin of the system (magenta curve) oscillates between 0.1 μ_B_ and about 0.5 μ_B_, with minima and maxima varying in phase with the F–F separation (green curve). It seems likely that this unpaired spin is associated with transfer of electronic charge from the substrate to the proto-adsorbate, resulting in a discernible weakening of the molecular bond.

Between around 450 and 550 fs into the simulation, however, matters change dramatically, with the fluorine atom that is closest to the surface (which we shall henceforth describe as the prompt fluorine atom) steering decisively toward the higher-lying atom of a nearby silicon dimer, while the F–F bond length stabilizes temporarily in the 1.80–1.90 Å range (green curve). While the corresponding Si–F distance drops ever faster toward first impact (red curve), the integrated spin modulus of the system (black curve) first rises sharply to around 2.0 μ_B_, before falling back to a little under 1.0 μ_B_. The latter change coincides with the affected dimer transitioning from a buckled to a nonbuckled geometry, suggesting that the previously empty dangling bond at the originally low-lying dimer atom accepts an electron to permit the formation of a Si–F bond involving the originally high-lying one.

In the simulation period from 550 to 700 fs, the prompt fluorine atom consolidates its nascent bond and commences high-amplitude stretch oscillations at a frequency of around 495 cm^–1^ (red curve). The tardy fluorine atom, as we shall call the second to arrive, now bereft of its partner, reaches its moment of maximum isolation at a simulation time of 645 fs, when it is more than 3.5 Å distant from any other atom. Nevertheless, both the integrated net spin and the integrated spin modulus (magenta and black curves) take values close to 0.6 μ_B_, suggesting that we cannot quite envision it as remaining entirely aloof from its surroundings; the latter measure ought to exceed 1.0 μ_B_ if a truly isolated fluorine atom were present in the system.

This eventful passage of play comes to an abrupt close, shortly after 700 fs, when the tardy fluorine atom collides with the higher-lying atom of a second silicon dimer, rebounding from a closest approach of around 1.6 Å (cyan curve) only to then collide, shortly before the 800 fs mark, with the only remaining unsaturated silicon atom of the first dimer (blue curve). From this moment on, the two fluorine atoms remain robustly attached to their respective bonding partners, settling into an in-phase stretch oscillation with a mean Si–F bond length of 1.63 Å and frequency of 785 cm^–1^ (red and blue curves). Such values are not outrageously different from the bond length of 1.55 Å and symmetric stretch frequency of 800 cm^–1^ reported for the Si–F bonds found in SiF_4_.^54,55^ The spin characteristics of the system continue to fluctuate occasionally, but tend increasingly toward a fully compensated configuration, consistent with saturation of both the dangling bonds belonging to a single dimer.

#### III.B.2 B/β Trajectory

Turning now to the B/β trajectory (Figure 2B/β) which leads to a final geometry of the type that we labeled i(c) in Table 2, the initial approach scenario is very similar to the case just described. The F_2_ molecule is induced, as a consequence of its interaction with the surface, to vibrate at a frequency close to 480 cm^–1^ (green curve) while the integrated net spin of the system oscillates in time with this motion, between extrema of 0.1 μ_B_ and 0.6 μ_B_ (magenta curve). This cruise phase persists until a little after 450 fs have elapsed, after which the integrated spin modulus rises sharply to around 2.0 μ_B_ (black curve) and then more slowly to around 2.5 μ_B_ by the 525 fs mark.

Interestingly, these abrupt changes in spin are not accompanied by equally abrupt changes in the system geometry. Throughout the 450–600 fs period, the molecule merely continues its drift toward the surface, accelerating gradually but otherwise showing few signs of distress. Reminiscent of the previous case, however, the F–F bond length does temporarily stabilize in the 1.80–2.00 Å range during this preimpact period of high spin. After a very brief contraction in the F–F bond length just after the 550 fs mark, coincident with a nearly complete disappearance of spin from the system, the decisive moment comes within the 600–650 fs period, when the spin dramatically recovers, the bond stretches once again, and the molecular center of gravity shifts laterally in the direction of a nearby silicon dimer.

For the following 250 fs (650–900 fs) the situation superficially resembles the end point of the preceding example, with both fluorine atoms binding to silicon atoms from the same dimer. Indeed, the spin collapses to zero at the start of this period, remaining so almost to its end, and the dimer buckling is once again undone. Looking closer, however, one sees that while one fluorine atom (we shall call it the tardy atom, as it is the second to collide with a silicon atom) undergoes gradually decaying oscillations in its Si–F bond length (blue curve) at a frequency that rapidly approaches 710 cm^–1^ from below, the other (the prompt atom) maintains a very high amplitude of visibly anharmonic oscillation in its Si–F bond length (orange curve) at a frequency that starts at around 380 cm^–1^ and declines. Shortly after the 900 fs mark, this prompt fluorine atom moves closer to the low-lying atom of a silicon dimer within the adjacent row (red curve) whereupon it is captured and dragged away from its original silicon partner. Just a little in advance of this, the integrated spin modulus (black curve) can be seen to have risen abruptly, to a value slightly in excess of 1.0 μ_B_, and indeed it continues to rise more gradually toward around 2.0 μ_B_ over the following 100 fs. Although this property will fluctuate somewhat as the trajectory proceeds further, it will spend the vast majority of its time within the 1.5–2.0 μ_B_ bracket. The integrated net spin, on the other hand, remains essentially zero, barring a brief blip (magenta curve) while the prompt fluorine atom actually exchanges silicon partners. The implication is that a single unpaired spin resides on each of the two silicon dimers involved, consistent with the existence of a single unsaturated dangling bond associated with each dimer atom that does not host a fluorine adatom, and that these spins happen to be antiparallel.

In the end, this trajectory settles into a regular pattern in which both Si–F bonds oscillate around mean lengths of 1.70 Å at frequencies of 655 cm^–1^ (red and blue curves). On both counts, it would appear that these bonds are slightly weaker than when both adatoms were attached to the same dimer, which must surely reflect the proximity of the unsaturated dangling bond in each instance. In comparison with the previous trajectory, where it will be recalled that one of the fluorine atoms was briefly separated from all other atoms by distances exceeding 3.5 Å, the tardy atom here is never more than 2.50 Å from another atom, and the prompt atom’s minimum separation never exceeds 2.15 Å until after it has first collided (at a distance of around 1.25 Å) with one of the silicon atoms. While the B/γ trajectory may sensibly be considered as an example of abstractive adsorption, therefore, the B/β trajectory probably cannot.

#### III.B.3 D/γ Trajectory

Whereas both trajectories discussed in detail above result in binding of fluorine adatoms to silicon dimer atoms, we had previously noted the possibility that one of the adatoms would instead attach to a second-layer silicon atom. This alternative scenario, labeled ii in Table 2 is satisfactorily exemplified by the D/γ trajectory, upon which we shall now focus (see Figure 2D/γ).

The trajectory commences with a now-familiar cruise phase, during which the molecule vibrates at a frequency close to 470 cm^–1^ and gradually accelerates toward the surface. Only at a simulation time of around 450 fs does anything of real note occur, when the integrated spin modulus rises to around 2.0 μ_B_ (black curve). At the same time, the F–F bond length stabilizes in the 1.90–2.00 Å range for a period of around 150 fs, before rising to a temporary maximum of just over 2.05 Å at the 675 fs mark (green curve). Throughout this high-spin period, the molecule drifts laterally toward the high-lying atom of a nearby silicon dimer, and the end of this phase marks the moment of impact for the lower-lying fluorine atom, which we now designate as prompt (red curve). The integrated spin modulus simultaneously falls to just below 1.0 μ_B_ (black curve) and the Si–F bond length of the prompt atom immediately starts to oscillate (red curve) and eventually converges upon a mean bond length of 1.61 Å and a frequency of 780 cm^–1^.

The tardy fluorine atom, meanwhile, reaches its moment of maximum isolation just after the 760 fs mark, when it is no closer than about 2.80 Å from any other atom. Evidently this is less isolated than was the equivalent atom in the B/β case, but it is still probably reasonable to consider the present trajectory to be an example of abstractive adsorption. Certainly the tardy fluorine atom does not experience its first impact with a silicon atom until just before the 800 fs mark, fully 125 fs later than the first impact of the prompt fluorine atom (blue curve). Furthermore, the tardy atom in fact bounces off this first silicon atom (which resides in the second layer, adjacent to the dimer that hosts the prompt atom) and then briefly attaches to another for around 200 fs (cyan curve) before returning to the first (blue curve). Ultimately, the corresponding Si–F distance oscillates at a frequency close to 800 cm^–1^ around a mean bond length of roughly 1.59 Å.

Curiously, the integrated spin modulus (black curve) drops to zero at around the time that the tardy atom establishes a bond with its second partner, and remains negligible from that point forward, barring a brief excursion just before the 1200 fs mark. From a geometric standpoint, however, the system would appear to feature two unsaturated dangling bonds–one associated with the dimer that hosts the prompt fluorine atom, and another associated with a third-layer atom whose bond with a second-layer atom is severed when the tardy fluorine atom adsorbs. There is, however, no reason why one of these dangling bonds ought not to be fully occupied while the other is empty, achieving the spin-compensated situation that we observe. It is, perhaps, noteworthy that the Si–F bond length and stretch frequency for the dimer-adsorbed prompt fluorine atom both lie closer to corresponding values found in the B/γ trajectory (where both dimer atoms are saturated) than to those from the B/β trajectory (where only one dimer atom is saturated). In passing, we also note that the Si–F bond length and frequency for the tardy fluorine atom, bound to a second-layer silicon atom, suggests that this may be the strongest such bond we have observed thus far.

#### III.B.4 C/α Trajectory

In view of the results discussed above, it will be interesting now to consider a situation in which both fluorine atoms eventually attach to second-layer silicon atoms, rather than to dimer atoms. Of the two trajectories leading to such a final geometry, labeled as iii in Table 2, we choose to describe in detail the C/α case (Figure 2C/α).

The first 450 fs of the simulation follows very closely the behavior we have described in respect of the previous three trajectories. The molecule accelerates smoothly toward the surface, vibrating at a frequency close to 480 cm^–1^. Then, for a period of around 250 fs, the F–F bond length stabilizes in the 1.80–2.00 Å range (green curve) while the integrated spin modulus (black curve) rises abruptly to around 1.8 μ_B_ and then more gradually up to around 2.5 μ_B_. Following the 700 fs mark, however, the spin rapidly declines to zero, coincident with one of the fluorine atoms (which we shall now identify as the prompt one) steering toward the high-lying atom of a nearby silicon dimer. The moment of impact occurs just before the 750 fs mark (orange curve) but by the 800 fs mark the prompt atom lies much closer to a neighboring second-layer silicon atom, to which it becomes attached (red curve). Meanwhile, the other fluorine atom (the tardy one) reaches its moment of maximum isolation at the 740 fs mark, when it lies just under 2.70 Å from its nearest neighbors. Although it swiftly passes within about 2.15 Å of a second-layer silicon atom, at around the 775 fs mark, it only forms what could plausibly be described as a bond with that atom shortly before the 900 fs mark (blue curve) some 150 fs after the prompt atom experienced its first impact. This eventful interval is marked by fluctuations in the integrated spin modulus, within the 0.80–1.80 μ_B_ range (black curve) but all spin vanishes from the system around the time that the tardy fluorine atom definitively forms its Si–F bond. Fluctuations in the spin do recur, most notably just before the 1100 fs mark, but they are swiftly extinguished as the simulation progresses.

Once both Si–F bonds are fully established (red and blue curves) they exhibit stretch frequencies close to 690 cm^–1^ and mean bond lengths of 1.63 Å. Quite remarkably, therefore, these bonds are apparently red-shifted by almost 14% from the frequency found in the D/γ trajectory for a Si–F bond involving a second-layer atom. The bond length is correspondingly slightly longer than in that case too, confirming that the proximity of two such bonds (each involving a second-layer atom attached to the same silicon dimer) renders both of them considerably weaker than they would have been alone.

#### III.B.5 A/γ Trajectory

Finally, we turn our attention to a case in which two fluorine atoms attach to one and the same silicon atom, breaking apart a surface dimer in the process. This type of geometry, labeled iv in Table 2 is exemplified by only a single instance, namely the A/γ trajectory (Figure 2A/γ).

Although this simulation begins predictably enough, with the molecule oscillating at a frequency of around 470 cm^–1^, the initial approach phase lasts for only the first 350 fs before differences with the four previous trajectories start to emerge. For the next 125 fs, the molecule executes an abnormally slow oscillation (green curve) corresponding to a frequency of around 265 cm^–1^, during which the integrated spin modulus (black curve) rises rapidly to around 2.0 μ_B_ before vanishing almost entirely. Coincident with the end of this excursion, the lower-lying (prompt) fluorine atom accelerates sharply toward the high-lying atom of the nearest silicon dimer, impacting at the 535 fs mark (red curve). At this point, the higher-lying (tardy) fluorine atom lies roughly 2.90 Å from its partner (green curve) drifting only quite slowly toward the surface, reaching its moment of maximum isolation around 140 fs later, when it is no closer than 3.10 Å from any other atom. Aside from a brief spell around the 500 fs mark, where it exceeds 1.5 μ_B_, the integrated spin modulus oscillates within the 0.5–1.0 μ_B_ range throughout this period.

At around the 625 fs mark, the tardy fluorine atom accelerates abruptly toward the midpoint of the nearest silicon dimer, to which the prompt atom is already attached (cyan and blue curves). It collides with the dimer’s unsaturated silicon atom at the 725 fs mark, almost 200 fs after the prompt atom first impacted the other one, but this immediately destabilizes the dimer bond and the corresponding Si–F distance increases rapidly to around 6.30 Å over the next 150 fs (cyan curve). By the 735 fs mark, the tardy fluorine atom’s closest neighbor is the same silicon atom that captured the prompt fluorine atom some 200 fs earlier. Prior to this moment, the first-formed Si–F bond oscillated at roughly 580 cm^–1^, but afterward it is blue-shifted toward around 740 cm^–1^ (red curve). The second-formed Si–F bond oscillates rather erratically, and at a considerably smaller amplitude, but ultimately exhibits a similar frequency (blue curve). The mean bond lengths are also a little erratic, varying slightly over multiple cycles, but typically lie within the 1.60–1.65 Å range. Spin, meanwhile, is largely extinguished after the 1050 fs mark, aside from a brief interlude at the very end of the plotted data (black curve). An extension of the simulation for a further 200 fs, beyond the end of the plotted data, confirms that the integrated spin modulus returns swiftly to zero and remains there for all but around 10 fs of that period. The lack of spin asymmetry at the 2-fold coordinated silicon atom, freshly liberated from its dimer, can readily be explained as arising from rehybridization of its two *sp*^3^ dangling bonds into an occupied orbital of *sp*^2^ character and an unoccupied one of essentially *p*-like character.

### III.C In Search of Rare Events

In retrospect, the rarity of ejection events ought not to be particularly surprising. The initial condition of the system is one in which all electrons are paired, including not only those associated with the incoming F_2_ molecule, but also those localized in the dangling bond of the higher-lying atom within each surface dimer. A final state in which a single F atom is ejected, however, necessarily implies that the ejected atom be a radical (with a single unpaired electron) and since the surface is nonmetallic one expects that it too must have radical character. Such an outcome, where a system with no radical character spontaneously acquires diradical character seems likely to be thermodynamically unfavorable (although the B/β trajectory does seem to manage this trick). Observation of such an outcome ought, therefore, to rest upon some quite particular molecular dynamics.

The expectation that single-atom ejection is rare does not, of course, mean to say that it cannot take place at all. Separately from the systematic calculations described above, we also undertook a number of trial-and-error calculations, starting with the system in various ostensibly plausible intermediate geometries that seemed likely to produce the desired result and running simulations both forward and backward in time. We have employed a similar approach with some success in several previous studies, albeit in those cases we were able to initiate our trajectories in genuine transition states of the various systems investigated. Here, no transition state exists, since dissociation in the present system appears to be barrierless, hence the need to try numerous guesses for a suitable state.

Eventually, after several attempts, we succeeded in finding a single trajectory (compiled by stitching together complementary forward- and reverse-time simulations) that linked an incoming F_2_ molecule with an adsorbed F adatom and an ejected F radical. The ejected atom even carried a not entirely outrageous outgoing velocity of 1802 m·s^–1^ (kinetic energy 0.32 eV) with the surface-normal component amounting to 1507 m·s^–1^ (kinetic energy 0.22 eV). This is a little less than the mean outgoing velocities of ejected atoms in previous simulations (at least 2000 m·s^–1^) albeit still higher that the range reported from experiment (1125–1181 m·s^–1^). Less conveniently, however, the incoming velocity of the F_2_ molecule, which cannot be controlled when making use of the time-reversal technique, was 1937 m·s^–1^ (kinetic energy 0.74 eV) with a surface-normal component of 1765 m·s^–1^ (kinetic energy 0.61 eV). Needless to say, these values far exceed the surface-normal velocity of 390 m·s^–1^ (kinetic energy 0.03 eV) used in the relevant experiments. Nevertheless, this trajectory does demonstrate that atom ejection is possible, even if the example we have uncovered is not particularly representative of such events as a whole. In this regard, it may be worth noting that we deliberately sought out trajectories in which the ejected F atom was “kicked off” the surface by collision with the nonejected F atom (after the latter rebounded from its initial interaction with the surface) in a kind of atomic Newton’s Cradle scenario. Clearly this represents a very particular manifestation of atom ejection, and not necessarily a common one.

At this stage, however, it is probably useful to note that the experimental results of Li et al.^5^ display a marked increase in the probability of ejection events as the surface coverage of fluorine increases (at least until the existing adsorbates eventually block all possible sites for further adsorption). At the time, the authors suggested that the effect stemmed from preadsorbed fluorine effectively passivating the surface, so that single F atoms liberated in the abstractive adsorption of F_2_ would be less likely to be captured prior to ejection from the surface. Alternatively, we might speculate that the radical character of a silicon dimer that hosts a single preadsorbed fluorine atom may play a key role. Certainly, such a site ought to be highly reactive, and we might expect that its unpaired electron may readily be donated to a highly oxidizing incoming F_2_ molecule, resulting in rapid dissociation and the transfer of radical character to a single outgoing F atom. That is, the overall radical character of the system would be conserved during the interaction (albeit transferred from the surface to a single atom) rather than substantially increased (as would be implied on an initially clean surface). Testing either of these hypotheses (i.e., blocking of capture sites, or direct enhancement of abstraction) would be a major undertaking, however, as the configuration space for trajectory initialization would be considerably multiplied in comparison with the work undertaken thus far. We therefore defer any such investigation to a future date.

Confining ourselves strictly to adsorption at the clean surface then, our interim conclusion must be that ejection of individual fluorine atoms is far rarer than was found in calculations that employed empirical potentials, and perhaps even rarer than suggested by experiment. Note, in this connection, that even the very first data point in the molecular beam studies of Li et al.^5^ corresponds to the build-up of a surface coverage of around 0.02 ML (with 1 ML implying a single adsorbed fluorine atom per first-layer silicon atom) and that the reported fraction of adsorption events involving atom ejection is already rising steeply at this point in the experiment. Extrapolation back to absolute zero coverage in such circumstances is always likely to prove tricky, and it is not altogether impossible that a small number of surface defects (vacancies, steps, or even a degree of fluorine contamination from prior experiments) may have a disproportionate influence in the low-coverage regime, leading to rather nonlinear behavior.

## IV Adsorption on the Monohydrogenated Surface

In comparison with results from the clean surface, our calculated trajectories for the monohydrogenated surface display greater variety in their eventual outcomes and greater complexity in their mechanisms. Nevertheless, the systematic approach taken above continues to yield worthwhile insights into the types of adsorption (and reaction) events that may occur.

### IV.A Categorisation of Final Geometries

Once again, we identify a number of major adsorption categories on the basis of the functional groups formed at the surface. In several cases, all involved atoms remain at the surface, leading to the following final conditions: (i) two fluorine atoms bound to two separate silicon atoms from a single dimer, each of the latter retaining its pre-existing hydrogen atom; (ii) two fluorine atoms bound in the manner just described, except bound to silicon atoms from two separate dimers; (iii) one fluorine atom bound as above, with the other inserted into a Si–Si bond; (iv) one fluorine atom bound to a second-layer silicon atom, with the other inserted into a Si–Si bond; and (v) two fluorine atoms bound to a single fourth-layer silicon atom, with two Si–Si bonds broken in the process. We subdivide categories (iii) and (iv) according to the layers involved in the Si–F–Si bonds that are formed, but otherwise there is little intracategory variation to be distinguished.

In addition, however, there are now also cases where only one of the fluorine atoms attaches to the surface, yielding the following final states: (vi) one fluorine atom replaces a hydrogen atom that was bound to a silicon dimer atom, the displaced atom having desorbed with the other fluorine atom in the form of HF; (vii) one fluorine atom bound to a silicon dimer atom that retains its pre-existing hydrogen atom, the other leaving with a hydrogen atom liberated from the other dimer atom, in the form of HF; and (viii) two silicon dimer atoms with dangling bonds, their hydrogen atoms removed to form two desorbing HF molecules. And finally, we note just one more possible outcome: (ix) both fluorine atoms replace hydrogen atoms bound to silicon dimer atoms, the two liberated atoms departing in the form of H_2_. These nine categories are represented among our trajectories in the ratio 4:1:2:3:1:1:1:1:1 (see Table 3) corresponding to the desorption of one H_2_ molecule and four HF molecules for every 15 F_2_ molecules impinging upon the surface.

**Table 3:** Summary of Outcomes from Trajectories in Which F_2_ Was Aimed at Five Different Sites of the Passivated Surface (A–E) in Three Different Orientations (α–γ) as Defined in Figure 1, Grouped According to the New Features Created at the Surface upon Adsorption^a^

new features	label	trajectory
2×(H–Si–F)_1_	i	A/α, A/γ, B/β, B/γ
2×(H–Si–F)_1_ &	ii	B/α
2×(Si−)_1_		
1×(H–Si–F)_1_ &	iii(a)	E/β (*m* = 1, *n* = 2)
1×(Si–F–Si)_*m,n*_	iii(b)	C/γ (*m* = 3, *n* = 4)
1×(Si–F)_2_ &	iv(a)	D/β (*m* = 1, *n* = 2)
1×(Si–F–Si)_*m,n*_ &	iv(b)	C/α, D/γ (*m* = 2, *n* = 3)
1×(Si−)_3_		
1×(F–Si–F)_3_ &	v	D/α
2×(Si−)_4_		
1×(Si–F)_1_ &	vi	E/α
1×HF		
1×(H–Si–F)_1_ &	vii	A/β
1×(Si=)_1_ &		
1×HF		
2×(Si−)_1_ &	viii	C/β
2×HF		
2×(Si–F)_1_ &	ix	E/γ
1×H_2_		

a The notation (Si–F) indicates a single fluorine atom bound to a silicon atom, while (H–Si–F) indicates a single hydrogen atom and a single fluorine atom bound to the same silicon atom, (F–Si–F) indicates two fluorine atoms bound separately to a single silicon atom, (Si−) indicates a silicon atom that gains a dangling bond where none were originally present, and (Si=) indicates a silicon atom that gains a second dangling bond where only one was originally present. A fluorine atom inserted into a Si–Si bond is represented as (Si–F–Si). In each case, a subscript indicates the surface layer in which the relevant silicon atom resides, taking the uppermost layer to be the first. Roman numerals label the four types of behaviour described in the text, subdivided by lowercase letters as appropriate.

### IV.B Descriptive Dynamics

In the accounts that follow, we shall expand upon the consistent color scheme adopted above for plots of electronic and geometric data over time. Magenta and black curves will, once again, be used to display the time evolution of integrated net spin and integrated spin modulus, respectively, while a green curve will always indicate the F–F interatomic distance. As before, we use a red curve to represent the Si–F distance corresponding to the prompt fluorine atom and the silicon atom to which it will eventually bind. If the prompt fluorine atom collides first with a different silicon atom, however, then that Si–F distance will also be shown, with an orange curve. The same logic is applied to the tardy fluorine atom, with blue (and cyan when needed) curves depicting the Si–F distances involving the atom to which it eventually binds (and the atom with which it first collides, if different). In the selected cases that follow (see the Associated Content statement for availability of data from all trajectories) it will occasionally be necessary to include some additional assignments, with olive and violet curves used to indicate certain H–F distances, a gray curve to indicate a particular H–H distance, and in one instance a brown curve to indicate a pair of important Si–Si distances. Details will be provided as the need arises.

#### IV.B.1 B/γ Trajectory

**Figure 3:**
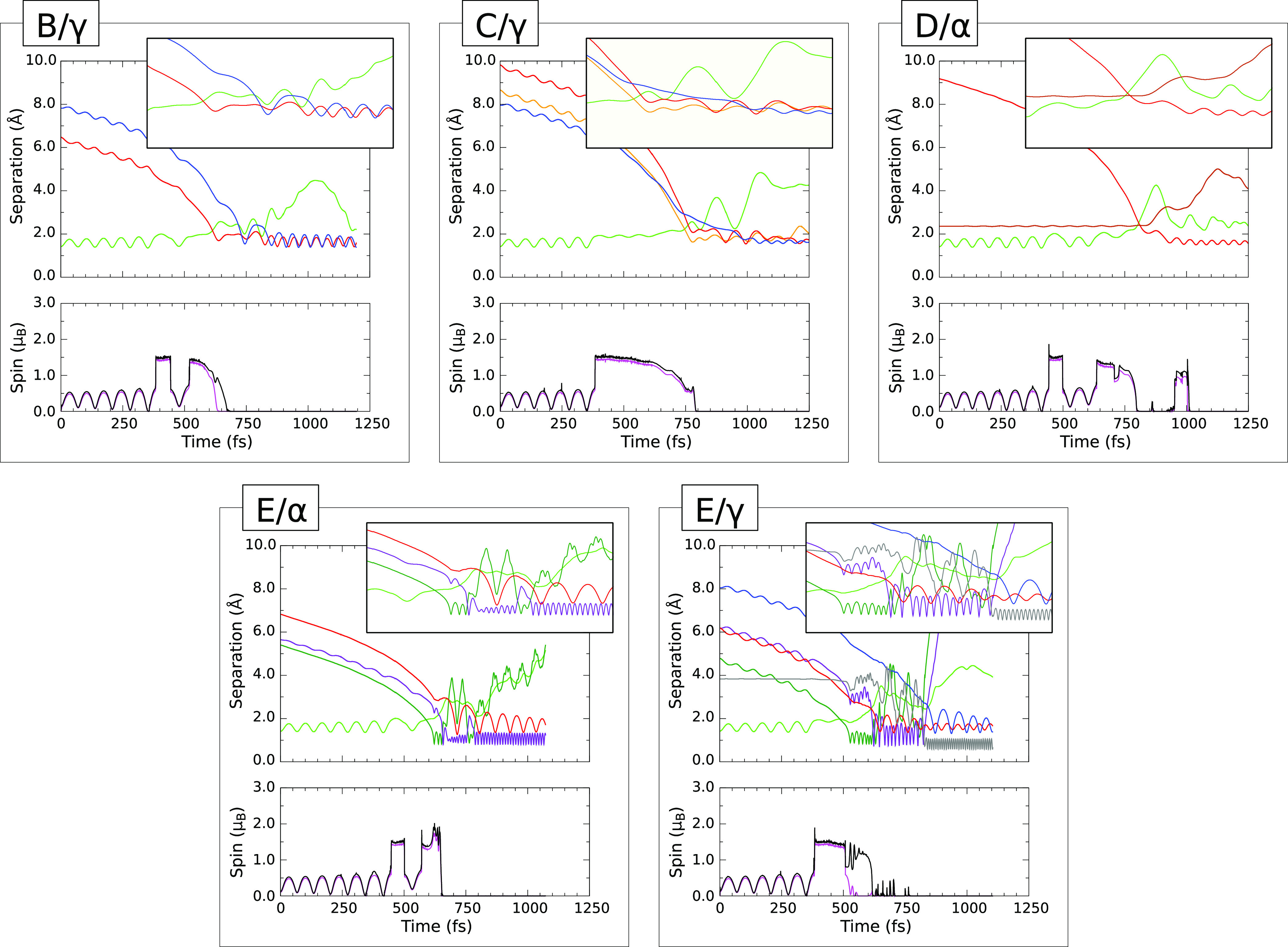
Evolution of selected trajectories on the passivated surface. Format and color schemes match those from Figure 2, with additional traces in certain of the upper panels as necessary. For D/α, we show one of two near-identical Si–Si distances in brown; for E/α and E/γ, we include an olive trace for an H–F distance involving the prompt fluorine atom and a violet trace for an H–F distance involving the tardy one; and for E/γ, we also include a single H–H distance in gray. Lower panels again show the integrated net spin in magenta and the integrated spin modulus in black.

We begin our survey of dynamics on the passivated surface with a case in which the final geometry features a broken dimer, with each of the two affected silicon atoms binding one hydrogen atom and one fluorine atom. This scenario, labeled i in Table 3, is exemplified in this instance by the B/γ trajectory (Figure 3B/γ).

In common with behavior noted above for the clean surface, the molecule initially drifts toward the surface under mild acceleration, exhibiting gentle oscillations in its bond length as it does so (green curve). The frequency of these vibrations is close to 475 cm^–1^, and this initial phase continues for the first 350 fs of the simulation, during which time the integrated net spin and integrated spin modulus (magenta and black curves) both fluctuate between about 0.1 μ_B_ and 0.6 μ_B_.

Over the following 125 fs, however, the molecule executes only a single oscillation, implying an effective frequency close to 265 cm^–1^, strongly reminiscent of a similar vibrational hiatus in the A/γ trajectory at the clean surface. The integrated net spin and integrated spin modulus (magenta and black curves) rise together to a plateau at about 1.5 μ_B_ for the duration, before falling back to just above 0.1 μ_B_ when the F–F bond reaches a local minimum at around the 475 fs mark (green curve). After this, the molecule nearly completes a second such oscillation, continuing to accelerate surface-wards and commencing a slow lateral drift in the direction of a nearby silicon dimer as it does so. Just before the 650 fs mark, the lower-lying fluorine atom (which we shall describe as prompt) passes almost perfectly through the midpoint of the dimer bond, which expands to a length of 3.90 Å (from a nominal length of 2.37 Å) to accommodate the interloper. Only a little over 100 fs later, however, the molecule has twisted into a near-horizontal attitude and the midpoint of the F–F bond lies very close to that of the dimer bond, with the latter having closed back up to a length of 2.78 Å in the meantime. In the interim, the system’s spin, which has been trending downward for some time, finally reaches zero and remains negligible thereafter.

Starting at the 800 fs mark, the molecule rapidly twists and elongates, finally allowing its constituent atoms to form individual bonds with the two dimer atoms. In time, these converge upon near-identical mean bond lengths of around 1.64 Å, oscillating in phase at a common frequency of about 730 cm^–1^ (red and blue curves). Compared with the end result of the B/γ trajectory on the clean surface, where both fluorine atoms also attached to different atoms from the same silicon dimer (albeit without coadsorbed hydrogen atoms), the Si–F bond length is here only marginally longer and the frequency is red-shifted by only around 55 cm^–1^ (7%). We might have expected more of a difference, but the presence of hydrogen and the breaking of the dimer evidently offset each other, to some degree, in their influence upon the strength of these bonds.

#### IV.B.2 C/γ Trajectory

As has been noted above, the C/γ trajectory leads to a final geometry quite unlike anything seen on the clean surface (labeled iii(b) in Table 3) in which one of the fluorine atoms is inserted into a Si–Si bond between the third and fourth surface layers (the other, more predictably, attaching to a first-layer dimer atom). Let us now examine the dynamics in more detail (Figure 3C/γ) to see just how this unusual state of affairs comes to pass.

Once again, the first 350 fs of the simulation is taken up with the gradually accelerating approach to the surface of a molecule oscillating at around 475 cm^–1^ (green curve). Shortly thereafter, the integrated net spin and integrated spin modulus of the system (magenta and black curves) rise sharply to around 1.5 μ_B_, and by the 400 fs mark the molecule’s bond length stabilizes at a little over 1.90 Å. Indeed, to this moment, the behavior seems little different from that of the B/γ trajectory, which we have only just discussed, but now the two scenarios diverge dramatically. For over 350 fs, while its bond length increases only very slightly (green curve) the molecule maintains gradual vertical acceleration down into the most open region of the surface structure. At the 775 fs mark, the lower-lying (prompt) fluorine atom skirts past one of the third-layer silicon atoms (orange curve) at a distance of 1.63 Å, which we should probably count as an impact, even though it falls marginally outside of the (somewhat arbitrary) descriptive rules we introduced in the Computational Method section. In fact, from this point onward, the motion of the prompt fluorine atom appears to be dictated not only by this particular third-layer atom but also by the fourth-layer silicon atom that the molecule was aimed at from the start of the simulation (red curve). At the 835 fs mark, the prompt fluorine atom passes through the midpoint of the Si–Si bond linking these two substrate atoms, and thereafter it settles into a somewhat erratic oscillatory pattern with stretch components for the two Si–F bonds in the ballpark of 525 cm^–1^. The corresponding mean bond lengths lie close to 1.77 Å, but are difficult to quantify precisely as they occasionally exhibit quite large deviations.

The tardy fluorine atom, meanwhile, drifts only gradually toward a nearby dimer atom, impacting at about the 1050 fs mark, and subsequently executes vibrational motion with a stretch frequency of about 690 cm^–1^ about a mean bond length of 1.63 Å.

#### IV.B.3 D/α Trajectory

One of the more surprising final geometries to emerge from our simulations is the one labeled v in Table 3, in which two fluorine atoms attach to a single silicon atom from the third layer. This behavior is seen only in the D/α trajectory (Figure 3D/α) but warrants attention nonetheless, since it comes very close to actually removing a silicon atom from the surface entirely. While we see no evidence of etching in our simulations, this particular trajectory gives some insight into how such a process might potentially occur.

Initially, the molecule approaches the surface in much the same manner seen in previously discussed trajectories, vibrating at a frequency close to 480 cm^–1^ while gradually accelerating over the first 400 fs. Shortly thereafter, however, the molecule executes a single slow oscillatory cycle, consistent with a frequency of roughly 300 cm^–1^, followed by another at an effective frequency around 430 cm^–1^ (green curve). The first of these two slower cycles is performed while the integrated net spin and integrated spin modulus (magenta and black curves) are stable at around 1.5 μ_B_, but for the second both measures fall back to values in the range 0.1–0.7 μ_B_, more consistent with behavior seen during the approach phase. After about the 650 fs mark, however, the F–F distance decisively stabilizes in the 1.85–2.05 Å range, while both spin measures increase to around 1.4 μ_B_ before commencing a gradual decline to around 1.1 μ_B_ over the next 125 fs.

By the 800 fs mark, the molecule has reached the same height as the top-layer silicon atoms, but it continues to move straight down through the gap between dimer rows. Strong interaction with the surface may nevertheless be inferred, however, from the system’s spin, which drops sharply to zero, and from the F–F distance, which rises rapidly, reaching a maximum of about 4.26 Å just after the 875 fs mark. Meanwhile, the third-layer silicon atom lying immediately beneath the molecule’s center of mass, which has been moving gently upward for some time, very nearly becomes collinear with the two fluorine atoms at this same moment, with the F–Si–F angle reaching a maximum of 167° and the two (very nearly identical) Si–F distances reaching local minima of around 2.12 Å (red curve).

At the same moment, this favored silicon atom begins to accelerate vertically upward, along with the two fluorine atoms, stretching the two Si–Si bonds that it makes with fourth-layer atoms in the process (brown curve). After a pause in its progress for the period between the 900 and 1000 fs marks, during which the spin temporarily recovers (magenta and black curves) and two well-defined Si–F bonds form (red curve), the nascent SiF_2_ group surges upward to a point, at around the 1125 fs mark, where the stretched Si–Si distances exceed 5.00 Å (brown curve). Indeed, at this moment, the escapee from the third layer is briefly the highest lying silicon atom of all, but nevertheless it always maintains two quite short Si–Si bonds that anchor it to the surface via the second layer. After this literal high point, the itinerant silicon atom sinks back toward the surface, albeit not (within the duration of our simulation) dropping back as far as its regular third-layer position. The system thus exhibits two fourth-layer dangling bonds, while the two Si–F bonds (red curve) execute stretch vibrations at a frequency close to 760 cm^–1^ and mean length of around 1.60 Å. Although in no danger of ever leaving the surface entirely during our simulation, it is clear that the SiF_2_ group is likely to be quite reactive and indeed susceptible to attack from subsequently arriving F_2_ molecules, potentially leading to etching processes that eventually liberate either SiF_3_ or SiF_4_ moieties from the surface.

#### IV.B.4 E/α Trajectory

Among those scenarios that generate one or two HF molecules, we shall focus upon the E/α trajectory (Figure 3E/α) corresponding to the final geometry labeled vi in Table 3. That is to say, the overall result of the reaction is to swap one fluorine atom from the molecule with one hydrogen atom from the surface. Just how this seemingly simple interchange takes place is, however, rather complex.

To begin with, the behavior broadly conforms to our expectations, with induced molecular oscillations at a frequency close to 485 cm^–1^ throughout an approach phase lasting a little over 400 fs, followed by a single oscillatory cycle at a much-reduced frequency, this time close to 285 cm^–1^ (green curve). The integrated net spin and integrated spin modulus both rise sharply to around 1.5 μ_B_ at the start of this final cycle and collapse to around 0.2 μ_B_ as it draws to a close at the 535 fs mark (magenta and black curves). Shortly thereafter, at about the 585 fs mark, the lower-lying (prompt) fluorine atom finds itself closer to the nearest hydrogen atom than it is to any other atom in the system, including its molecular partner, and about 35 fs later these two atoms collide (olive curve). The shortest Si–F distance at this stage is still in excess of 2.95 Å, while the relevant H–Si distance has increased from 1.48 to 1.96 Å. It is clear, therefore, that a new molecular species has been formed, meaningfully separate from the surface. Both the integrated net spin and integrated spin modulus peak briefly in the ballpark of 2.0 μ_B_ at this time, and since the values expected for an unsaturated silicon dimer atom (after abstraction of its hydrogen atom) would only be 1 μ_B_, it seems clear that the newly minted HFF moiety has radical character. Such a species is rather unstable, of course, and the sequence of events that follows is fascinatingly frenetic.

First, the impacted hydrogen atom and the prompt fluorine atom begin a stretch oscillation at a frequency that starts close to 1850 cm^–1^ but rises swiftly to around 2780 cm^–1^ (olive curve). Meanwhile, the F–F bond length (green curve) increases from around 2.00 Å to around 2.35 Å in the first 30 fs after the initial impact, perhaps impelled toward the 2.72 Å equilibrium bond length predicted for the HFF radical.^57^ After just two oscillatory cycles, however, and before the preferred radical geometry can be fully achieved, the hydrogen atom darts across from the vicinity of the prompt fluorine atom to that of the tardy one (at the 650 fs mark) before settling into a vibration with a stretch frequency around 3240 cm^–1^ that begins at the 680 fs mark and persists for the next 70 fs (violet curve). At the very beginning of this period (i.e., 650 fs), both spin measures fall abruptly to zero, at which value they will stay for the remainder of the simulation. Again, it is worth emphasizing that the shortest Si–F distance still exceeds 2.95 Å at this point, so this drop cannot reasonably be explained by the formation of a covalent adsorbate–substrate bond. Instead, we suppose that charge transfer must occur, implying that the HFF radical is probably converted to a more stable HFF^–^ anion (albeit not in its preferred hydrogen-centered geometry). Wild excursions in the distance between the abstracted hydrogen atom and the prompt fluorine atom (olive curve) span the range 1.75–3.90 Å at a frequency around 795 cm^–1^ and reflect precessional and nutational motion of the H–F bond around the F–F bond axis. The latter varies at a similar frequency over the range 2.65–2.90 Å during this period (green curve).

Despite this brief interval of stability for the (presumed anionic) molecular species, one major event does occur within the 650–750 fs window, which is that the prompt fluorine atom finally impacts the now-unsaturated silicon dimer atom at the 715 fs mark (red curve). By the end of the simulation, the corresponding Si–F bond exhibits stretch oscillations at a frequency close to 670 cm^–1^ around a mean length of about 1.67 Å (the former probably still rising and the latter probably still falling).

Wresting our attention back to the abstracted hydrogen, meanwhile, we find that it briefly transfers back to the ambit of the prompt fluorine atom (olive curve) for a period of about 25 fs centered around the 770 fs mark, but after barely more than a single oscillatory cycle at a frequency close to 2780 cm^–1^ it hops straight back to the tardy fluorine atom and executes stretch-like oscillations at a frequency rising from around 2925 cm^–1^ to around 3270 cm^–1^ over the remainder of the simulation (violet curve). The fundamental vibrational frequency of a free HF molecule ought to be 3959 cm^–1^,^56^ but our desorbing molecule is very highly excited and therefore experiences a substantial red-shift due to anharmonicity.

Averaging over five complete vibrational cycles, leading up to the 1070 fs mark in our simulation, the HF molecule’s center of mass registers a mean translational velocity of 1716 m·s^–1^ at an angle of 32° from the surface normal (see the following section for a discussion of how we extract properties from the raw molecular dynamics data). The molecule thus carries mean translational kinetic energy amounting to 0.31 eV, while its mean rotational and mean vibrational kinetic energies are 0.72 and 0.62 eV, respectively. The mean vibrational potential energy (relative to the minimum of the H–F bond’s potential well) is 0.88 eV, and the molecule’s total robvibrational energy (summing kinetic and potential contributions) of 2.23 eV significantly exceeds its translational energy (by a factor of around 7). Vibrational anharmonicity and centrifugal distortion combine to substantially stretch the internal bond of the desorbing molecule, which attains a mean length of 1.10 Å (some 16% longer than in static equilibrium).

#### IV.B.5 E/γ Trajectory

Finally, we turn to the single scenario that led to the generation of a hydrogen molecule, namely the E/γ trajectory (Figure 3.E/γ). The final geometry, labeled ix in Table 3, involves replacement by fluorine atoms of two hydrogen atoms from adjacent silicon dimers, but once again the precise sequence of events that leads to this result is quite complicated.

For the first 350 fs of the simulation, everything proceeds in accord with the familiar pattern described for all nine trajectories discussed thus far. The molecule gradually accelerates toward the surface, vibrating as it does so at a frequency of about 480 cm^–1^. Then, the F–F bond length stabilizes in the 1.80–2.00 Å range until beyond the 550 fs mark (green curve). The integrated net spin and integrated spin modulus (magenta and black curves) both rise sharply to around 1.5 μ_B_ around the 380 fs mark, and decline only gradually over the subsequent 120 fs.

At the 525 fs mark, however, things change abruptly, as the lower-lying (prompt) fluorine atom undergoes its first collision with its nearest hydrogen atom. The corresponding H–F distance then begins to oscillate, first at a frequency around 1960 cm^–1^ but gradually rising toward 2180 cm^–1^ (olive curve). The F–F distance also increases, reaching around 2.35 Å at about the 600 fs mark (green curve) while the shortest Si–F distance remains above 2.55 Å up to the same time. In several respects, therefore, the situation resembles that noted in our discussion of the E/α trajectory, where it was suggested that one might invoke the notion of a well-defined HFF radical. Here, however, the integrated spin modulus (black curve) continues to decline throughout the 525–600 fs period, and the integrated net spin (magenta curve) falls to zero. It seems, therefore, that any radical quality attached to the HFF moiety must only be partial at most.

Also reminiscent of the previously discussed E/α trajectory is the fact that the hydrogen atom jumps from the ambit of one fluorine atom to that of the other, in this instance at around the 625 fs mark. After a brief period of instability, the H–F bond length involving the tardy fluorine atom eventually settles into a fairly steady oscillation at a frequency close to 2265 cm^–1^ (violet curve) that persists until beyond the 800 fs mark. Notably, the system’s spin is extinguished entirely during this period, never to return, implying the loss of any radical character that may have existed. The prompt fluorine atom, meanwhile, collides with the now unsaturated silicon atom at the 650 fs mark, and the resulting bond undergoes stretch oscillations thereafter, settling over time toward a frequency of roughly 770 cm^–1^ and a mean bond length of around 1.62 Å (red curve). The F–F distance (green curve) rises to 3.5 Å at the 650 fs mark, suggesting strongly that adsorption of the prompt fluorine atom leaves the tardy fluorine atom isolated apart from its newly acquired hydrogen atom. Compared with the expected stretch frequency for a gas-phase HF molecule, experimentally measured at 3959 cm^–1^, the value noted above is red-shifted by 1696 cm^–1^ (43%) but there are likely to be strong anharmonic effects in play, due to the vibrationally excited nature of nascent species.

In the course of its highly excited motion within the HF moiety, the abstracted hydrogen atom approaches a nearby adsorbed hydrogen atom quite closely on a number of occasions, finally culminating, at around the 825 fs mark, in an actual collision and the subsequent formation of a bond (gray curve). The H–H distance oscillates at a frequency close to 3931 cm^–1^ as the molecule desorbs. This is red-shifted by 228 cm^–1^ (6%) relative to the expected value of 4159 cm^–1^ for the gas-phase molecule,^56^ but again much of this discrepancy is probably explained by the highly excited state of the molecule in our simulation. The tardy fluorine atom, now bereft of its bonding partner, collides rather promptly with the silicon atom left unsaturated by abstraction of the second hydrogen atom (blue curve) forming a bond that vibrates at around 605 cm^–1^ with a mean length of about 1.72 Å (albeit these are likely still changing at the end of our simulation).

The newly created H_2_ molecule, on the other hand, departs from the surface with a mean center-of-mass translational velocity (calculated over five complete vibrational cycles up to the 925 fs mark, well after desorption but before collision with the back surface of the next slab) of 9223 m·s^–1^ at an angle of 41° from the surface normal. Its mean translational kinetic energy is therefore 0.90 eV, while its mean rotational and mean vibrational kinetic energies are 0.09 and 0.39 eV respectively (once again, see the following section for details of these calculations). The mean vibrational potential energy (relative to the minimum of the H–H bond’s potential well) is 0.45 eV, meaning that the molecule’s total rovibrational energy amounts to 0.94 eV, quite comparable with its translational energy. The internal bond of the desorbing molecule is stretched (compared with static equilibrium) rather less than was the case for the HF molecule that desorbed in the preceding trajectory, attaining a mean length of 0.85 Å (a 12% increase). This reflects the somewhat smaller rovibrational energy in the present case, implying correspondingly less anharmonic/centrifugal distortion.

## V Analysis of Desorbing Molecules

To analyze the motion of desorbing diatomic molecules, we first calculated a mass-weighted mean velocity at each time step, which will be synonymous with the velocity of the molecule’s center of mass in the surface frame. That is, we have
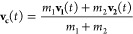
3for the center-of-mass velocity of a molecule comprising atoms of masses *m*_1_ and *m*_2_ with individual velocities **v**_**1**_(*t*) and **v**_**2**_(*t*). The translational kinetic energy of the molecule (in the surface frame) then emerges straightforwardly from
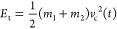
4where *v*_c_(*t*) = |**v**_**c**_(*t*)| is the center-of-mass speed. For the two cases we study here (HF and H_2_ desorption in the E/α and E/γ trajectories of the monohydrogenated surface), we confirm that this property is essentially constant during the period analyzed (standard deviation less than 1% of the mean value), indicating a lack of linear force exerted by the surface upon the desorbing molecule.

If we now transform to the molecule’s center-of-mass frame, the individual atomic velocities become
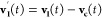
5and
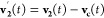
6so that the total kinetic energy of the molecule (in this frame) is given simply by
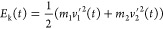
7with *v*_1_^′^(*t*) = |**v**_**1**_^′^(*t*)| and *v*_2_^′^(*t*) = |**v**_**2**_^′^(*t*)| at any given time-step. This kinetic energy includes both rotational and vibrational contributions but clearly no translational component.

By resolving the velocities, **v**_1_^′^(*t*) and **v**_2_^′^(*t*), perpendicular to the instantaneous orientation of the molecular bond, we extract the angular velocity of the molecule for rotation around its center of mass. The position of that center of mass is, of course, given by
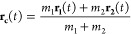
8where **r**_**1**_(*t*) and **r**_**2**_(*t*) are the instantaneous positions of the two atoms in the surface frame.

In the center-of-mass frame, meanwhile, the positions of those atoms may be written as
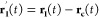
9and
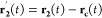
10so that we may obtain the molecule’s moment of inertia as
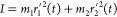
11at any given time-step.

Armed with the molecule’s angular velocity and moment of inertia, we readily calculate its angular momentum from their product. Once again, we have confirmed that this is essentially a conserved quantity over the period analyzed (standard deviation in magnitude less than 1% of the mean value) indicating a lack of significant torque exerted by the surface upon the desorbing molecule.

**Figure 4:**
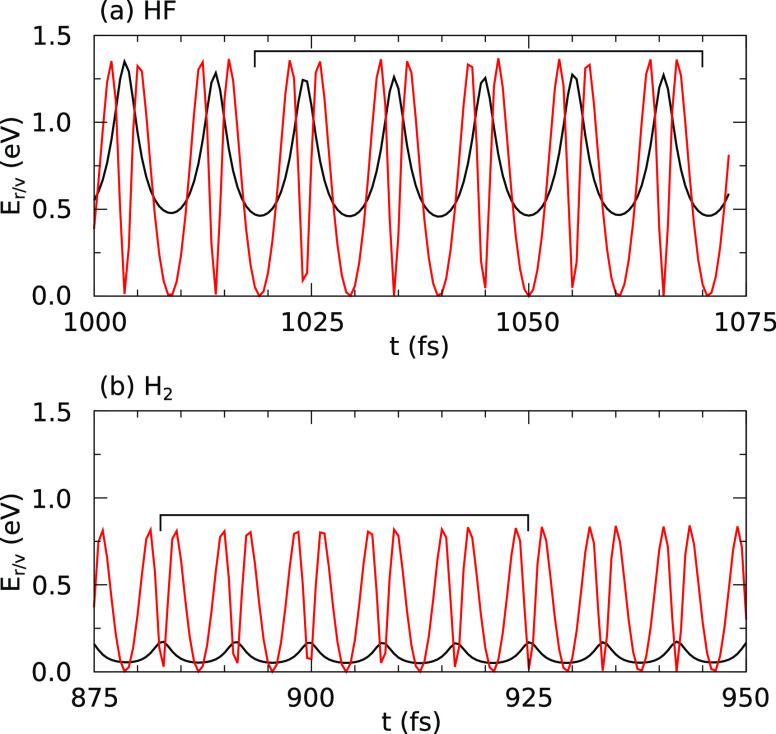
Molecular kinetic energies for (a) HF in the E/α trajectory and (b) H_2_ in the E/γ trajectory, separated into rotational (*E*_r_) and vibrational (*E*_v_) components in black and red, respectively. Time ranges over which mean values have been evaluated (five full cycles in each case) are marked with horizontal brackets.

The rotational kinetic energy is given simply by
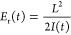
12where *L* = |**L**| is the magnitude of the angular momentum. Clearly, since *L* remains constant while *I*(*t*) continually varies as the bond length oscillates, we ought not to expect the rotational kinetic energy to be constant. Instead, energy will periodically shuttle back and forth between rotational and vibrational forms. Furthermore, since we know both the rotational kinetic energy *and* the total kinetic energy (still working in the center-of-mass frame), we may easily calculate the vibrational kinetic energy as
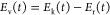
13at any given moment in time. Representative data for the two systems studied are presented in Figure 4. Although rotational and vibrational kinetic energies clearly oscillate, it is nevertheless evident that a rolling mean calculated over an integer multiple of vibrational cycles will be meaningful. Where mean rotational and vibrational kinetic energies are quoted in the main text, they should be understood to imply values averaged over five complete cycles (see Table 4).

**Table 4:** Mean Angular Momentum, Together with Mean Translational, Rotational, and Vibrational Energies^a^

	*L̅* (*ℏ*)	(eV)	(eV)	(eV)	
HF	36.639	0.308	0.723	0.620	0.884
H_2_	7.047	0.895	0.091	0.394	0.452

a Also given is the mean deviation in potential energy relative to the potential minimum of the appropriate potential curve from Figure 5 and Table 5. For both molecules, data was taken from five complete vibrational cycles, ending for HF at the 1070 fs mark of the E/α trajectory and for H_2_ at the 925 fs mark of the E/γ trajectory.

Now, it is worth emphasizing here that the kinetic energy, *E*_k_(*t*), is also not a conserved quantity, even though we should expect the total energy of a sufficiently isolated molecule to be fixed. The reason for this variation in kinetic energy is, of course, that the potential energy of the molecule will also be varying, ideally in precise antiphase with the kinetic. Indeed, we can predict that the potential energy of the molecule should behave as
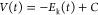
14where *C* is an arbitrary constant reference potential. Plotting the potential energy as a function of time would, therefore, not be a particularly fruitful exercise, merely replicating information already implicit in Figure 4. On the other hand, plotting the potential obtained from eq 14 against the instantaneous bond length, *d*(*t*), will actually sketch out the potential energy curve experienced by the molecule during its oscillations. For both molecules, the curve is well approximated by a Morse potential, which is to say
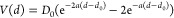
15where *d*_0_ is the equilibrium bond length, *D*_0_ is the bond dissociation energy, and *a* controls the width of the well (for a given value of *D*_0_). Fitted values are given in Table 5, and the goodness of fit should be evident from Figure 5.

**Figure 5:**
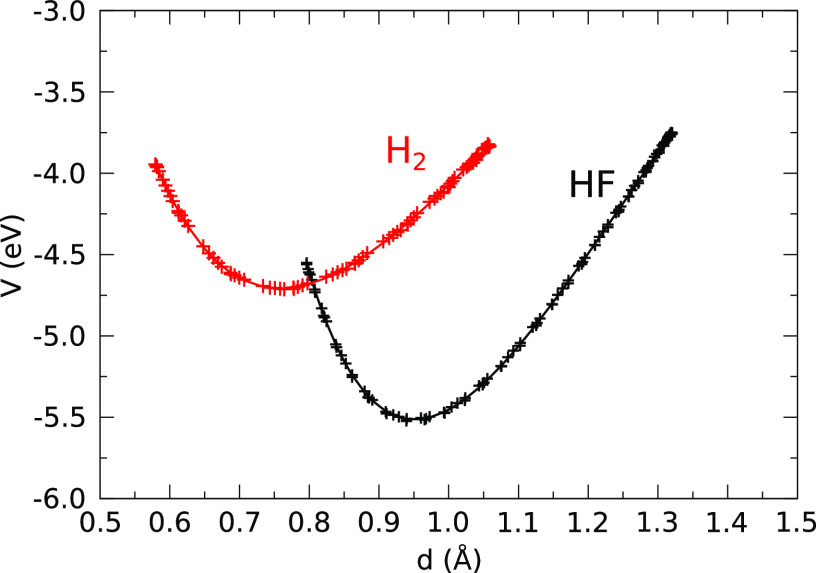
Molecular potential energy, *V*(*d*), inferred from eq 14, plotted against interatomic separation, *d*, for HF (black) and H_2_ (red). Data points were taken from five complete vibrational cycles, ending for HF at the 1070 fs mark of the E/α trajectory and for H_2_ at the 925 fs mark of the E/γ trajectory. Solid curves are fits to the data based on the Morse potential (eq 15). In each case, the parameter *C* in eq 14 has been adjusted so that the fitted curve would asymptotically approach zero at infinite separation.

**Table 5:** Fitted Parameters for Morse Potentials of the Form Given in Eq 15^a^

	*D*_0_ (eV)	*d*_0_ (Å)	*a* (Å^–1^)	(Å)
H–F	5.514	0.951	2.257	0.152
H–H	4.708	0.758	1.895	0.089

a Also noted is the mean deviation in bond length relative to the potential minimum, evaluated over the data used in the fit. For both molecules, data was taken from five complete vibrational cycles, ending for HF at the 1070 fs mark of the E/α trajectory, and for H_2_ at the 925 fs mark of the E/γ trajectory.

It is interesting to note that, for both molecules, the potential energy rises to a rather greater value on the stretched side of the minimum than on the compressed side. This is a consequence of the centrifugal force that would be present in the noninertial (translating *and* rotating) frame that one would need to adopt in order to render the molecule’s motion purely vibrational in character. As a result, the mean bond length (averaged over an integer multiple of vibrational cycles) exceeds the equilibrium value that would pertain for a nonrotating molecule. Furthermore, the mean potential energy does not equal the mean vibrational kinetic energy, as would be the case for a nonrotating harmonic oscillator.

## VI Conclusions

In summary, our results for F_2_ adsorption at the clean surface show that the ejection of a single fluorine atom from the surface, while not impossible, is likely to be a rather rare event, requiring a somewhat particular conjunction of initial conditions (as discussed above, at the end of the section on clean-surface adsorption). In this respect, agreement with experiment is rather better than was achieved with empirical potentials,^1−4^ underlining the importance of first-principles methods whenever bonds are made or broken. Furthermore, our focus upon a small number of highly symmetric trajectories, and indeed neglect of initial thermal energy, permits us to analyze both the geometric and electronic structure of individual trajectories to identify key features that dictate the eventual outcome in each case. We have presented detailed descriptions of the dynamic behavior leading to five distinctly different surface configurations, noting three in which abstractive adsorption (where the prompt fluorine atom adsorbs immediately, and the tardy fluorine atom only after a period of effective isolation) can reasonably be said to occur. Clearly, these results should be interpreted cautiously from a quantitative perspective, since the relatively small number of rather symmetric trajectories studied does not lend itself to achieving statistical significance in a formal sense. Nevertheless, the qualitative difference between the present first-principles approach and previous empirical calculations should be abundantly clear. It ought also to be equally clear that molecular dynamics simulations based on forces derived from first-principles should be considered inherently superior to those based on empirical potentials that typically are fitted only to the structures, energies, and vibrations of intact molecules.

On the monohydrogenated surface, our calculations reveal a variety of different behaviors, including several in which the surface hydrogen atoms play only a spectatorial role but others in which they are key participants in the events that transpire. In particular, we identify three trajectories in which HF desorbs and one in which H_2_ is generated. In all of these, it is arguable that a short-lived HFF intermediate, which may possess some degree of radical character, is crucially important in mediating the adsorption and desorption processes. We note some similarity to our previous study of ozone adsorption at the same monohydrogenated surface, where a short-lived HO_3_ radical was likewise found to be of central importance. Desorbing molecules are found to be highly rovibrationally excited, particularly in the case of HF in comparison with H_2_.

The wider conclusion that we wish to emphasize is, however, that first-principles molecular dynamics provides a critical benchmark against which to judge results obtained from calculations performed with empirical potentials. In recent years, the advent of reactive force fields, such as ReaxFF,^58^ holds out the promise of modeling bond making and breaking processes with fidelity approaching that of DFT while permitting much larger systems to be simulated for much longer durations or from many more initial points in phase space. Nevertheless, it is generally true that reactive force fields are not so transferable between systems as one might wish and that bespoke modifications are often necessary. In light of this, benchmarking against accurate simulations for challenging reference systems becomes all-important in validating the method. Similarly, it would be tempting to employ approximate DFT methods, for example, the tight-binding approach embodied in the DFTB+ code,^59^ but here too it would be wise to evaluate performance against reference calculations carried out using more traditional DFT methods. Once convinced of the fidelity of high-throughput approximate calculations, the focus of attention must necessarily then shift to the automation of analysis for such large data sets as would then become feasible to accumulate. Here again, insight from calculations of the type reported in the present work will be invaluable in guiding the development of such data analysis techniques.

## Supporting Information Available

Graphs summarizing the variation of bond lengths and spin values and illustrations of final geometries (PDF)

## Notes

The trajectories and spin data analyzed in this study are openly available in the University of Cambridge Data Repository at DOI: 10.17863/CAM.78699.
