# Supplementary material for: First-Principles Dynamics of Fluorine Adsorption on Clean and Monohydrogenated Si{001}
Source: Langmuir. 2022 Jun 1;38(23):7256–71. doi: 10.1021/acs.langmuir.2c00740 (PMC9202358; doi:10.1021/acs.langmuir.2c00740)
Supplement: Supplementary file 1 — la2c00740_si_001.pdf [file la2c00740_si_001.pdf]

Supplementary Information relating to: First-Principles Dynamics of Fluorine Adsorption on Clean and Monohydrogenated Si{001}

Ian Y. H. Wu and Stephen J Jenkins

Yusuf Hamied Department of Chemistry, University of Cambridge, Lensfield Road, Cambridge, CB2 1EW, UK

Geometries, Bond Lengths, and Spin

Figs. 1-33 represent geometries following adsorption of F_2 at the clean or monohydrogenated Si{001} surface, with fluorine atoms shown as green spheres, silicon as beige spheres, and hydrogen as white spheres. Each image shows a top-down orthographic view of a single c(4×4) unit cell, and the two fluorine atoms derive from a single molecule. Snapshots have been taken close to the end of the simulation period, with the precise moment chosen to provide greatest clarity in the relative positions of the atoms. It should be understood that the atoms are, of course, in motion and might obscure one another from time to time, so a snapshot from the literal end of the simulation may not always be as informative as might have been hoped.

Figs. 34-66 show selected interatomic distances and spin characteristics, with colour coding following the pattern established in the main text. In all cases, the F–F distance is plotted as a green trace. A red trace indicates the Si–F distance between the prompt fluorine atom and the silicon atom to which it will eventually bond, supplemented with an orange trace in cases where the distance to a different silicon atom may also be of interest. Blue and cyan traces play the same roles for the tardy fluorine atom. Where red and blue traces would be indistinguishable, only the red is shown. In cases when it is desirable to show H–F distances, any relating to the prompt fluorine atom are indicated with an olive trace, and any relating to the tardy fluorine atom with a violet trace. A grey trace is used to indicate an H–H distance in one case only, while in another a brown trace is used to indicate one particular Si–Si distance. Magenta and black traces show integrated net spin and integrated net spin modulus, respectively.

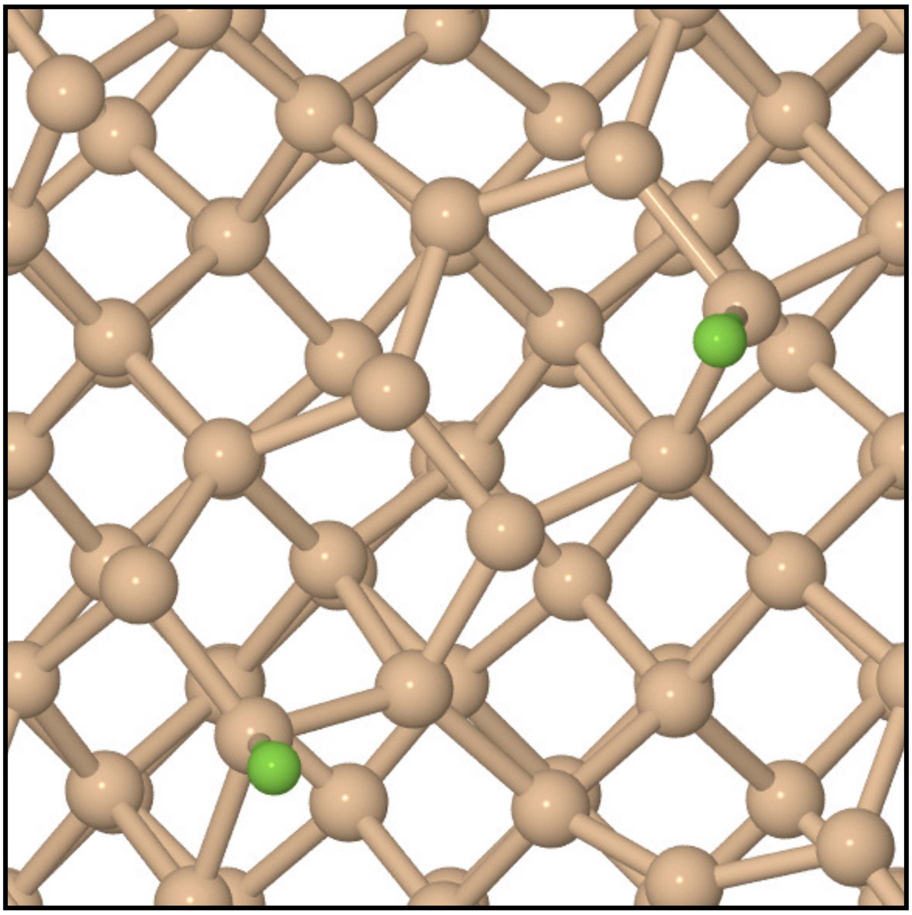

Figure S1: Geometry following adsorption on the clean Si{001} surface via the A/α trajectory.

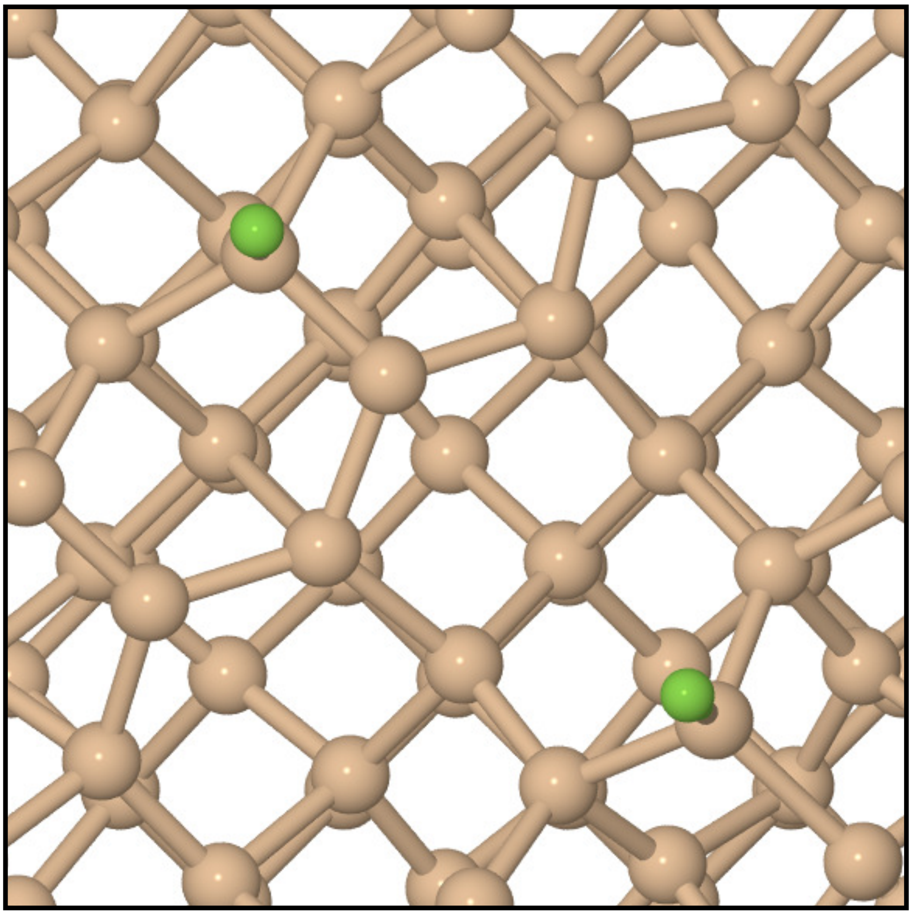

Figure S2: Geometry following adsorption on the clean Si{001} surface via the A/β trajectory.

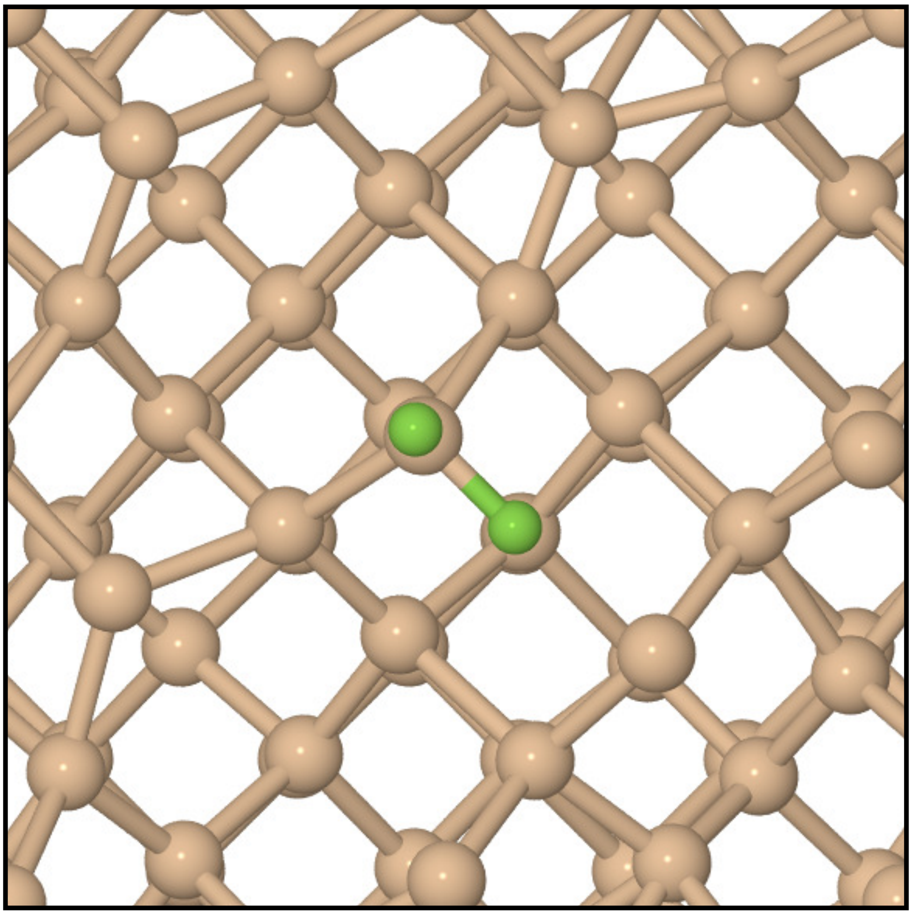

Figure S3: Geometry following adsorption on the clean Si{001} surface via the A/γ trajectory.

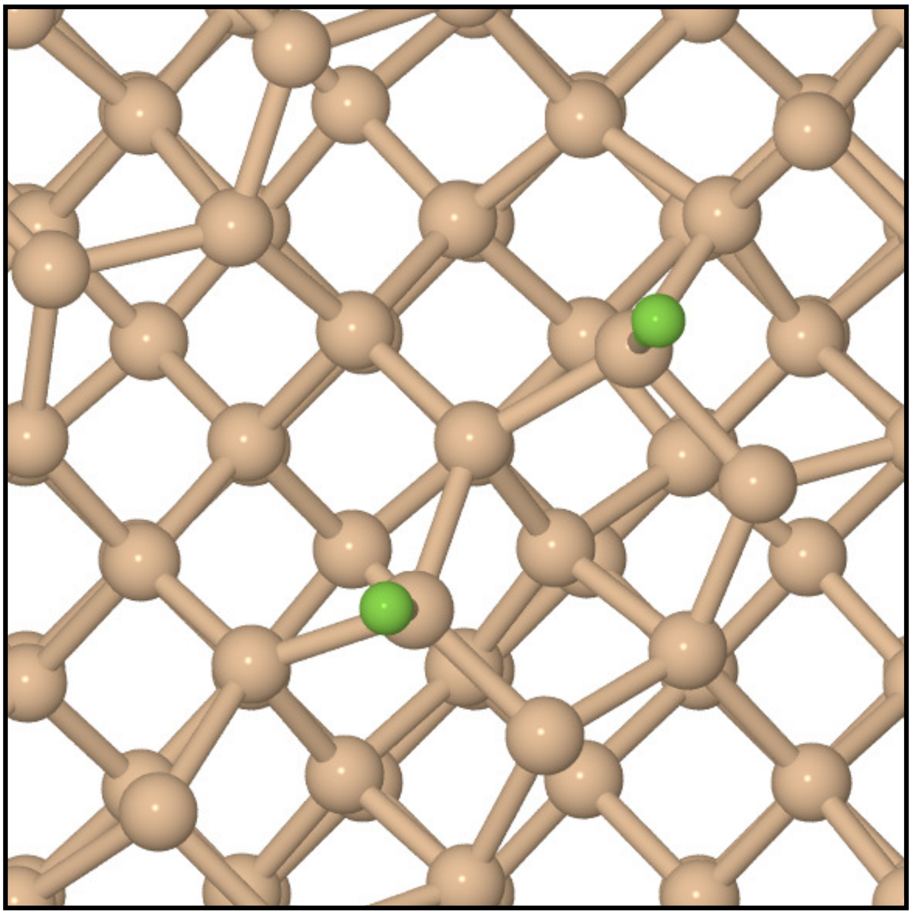

Figure S4: Geometry following adsorption on the clean Si{001} surface via the B/α trajectory.

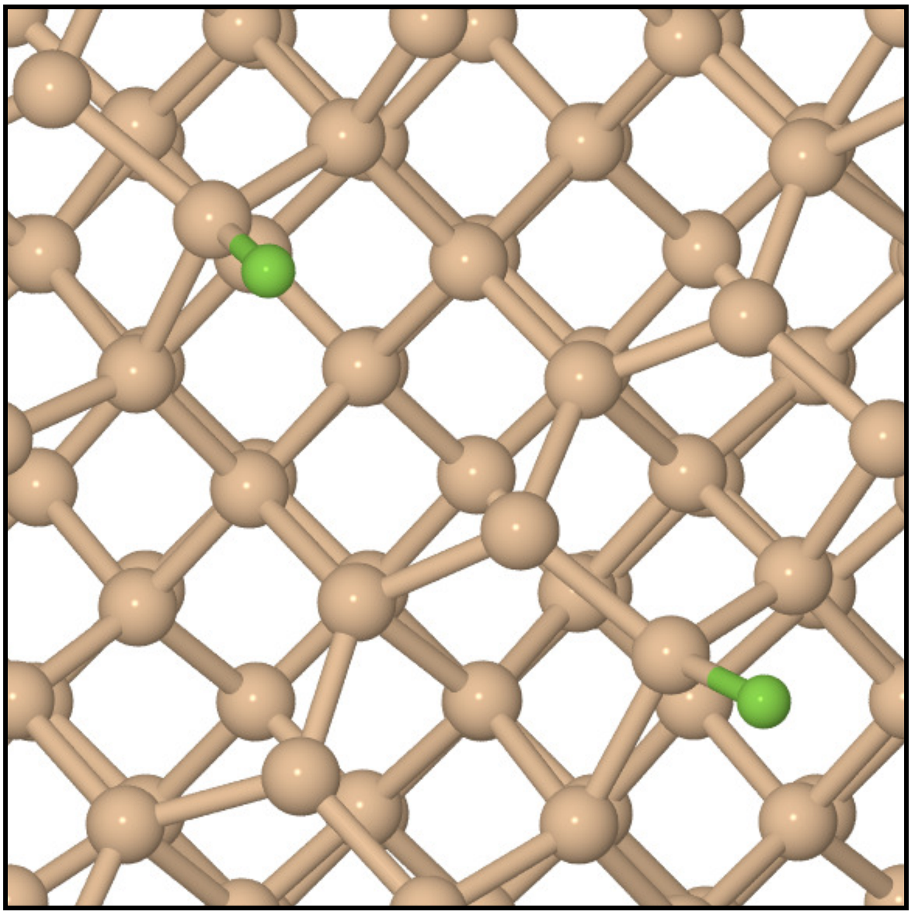

Figure S5: Geometry following adsorption on the clean Si{001} surface via the B/β trajectory.

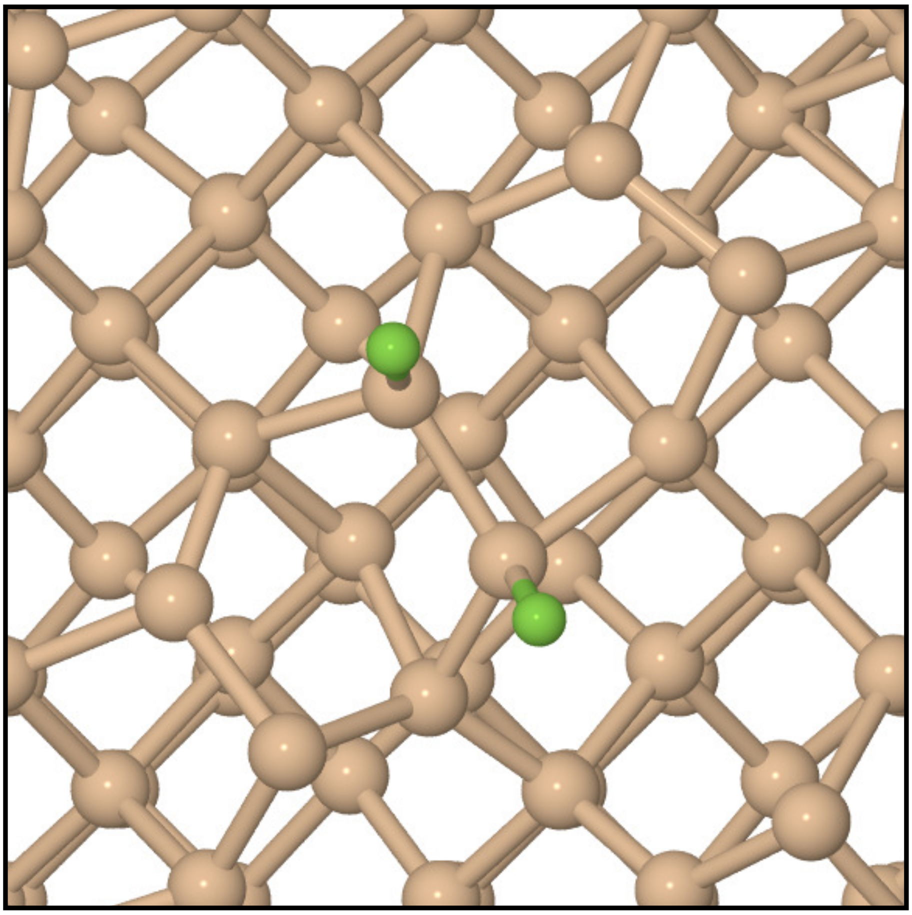

Figure S6: Geometry following adsorption on the clean Si{001} surface via the B/γ trajectory.

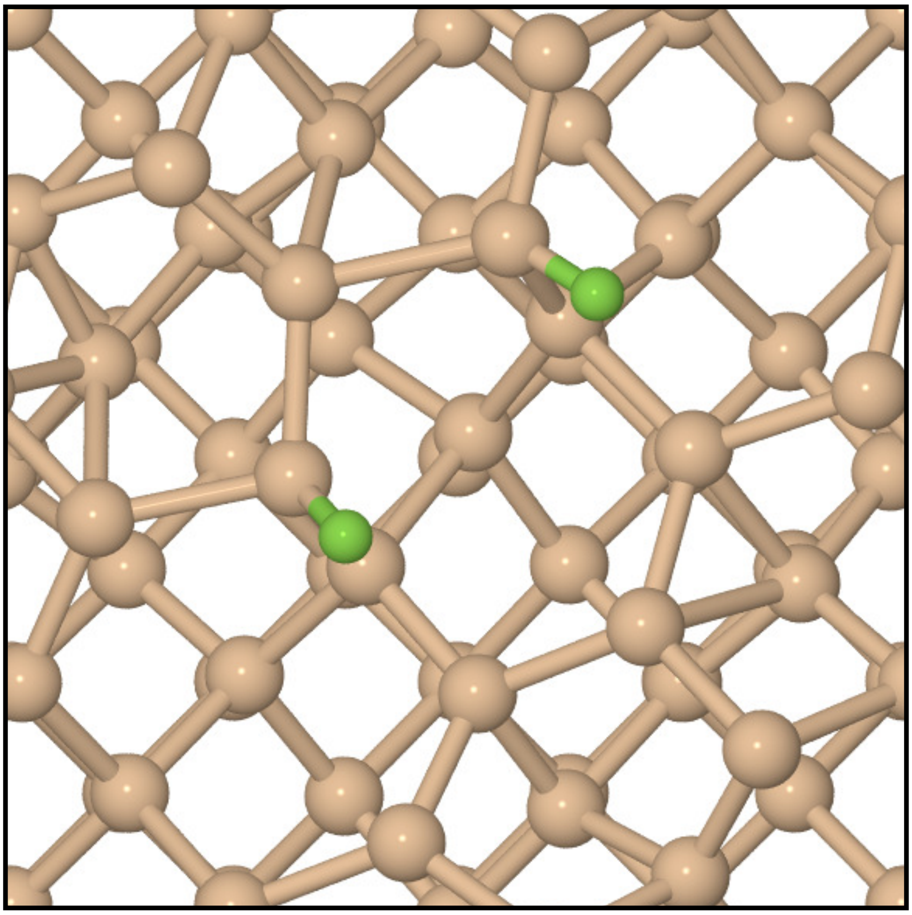

Figure S7: Geometry following adsorption on the clean Si{001} surface via the C/α trajectory.

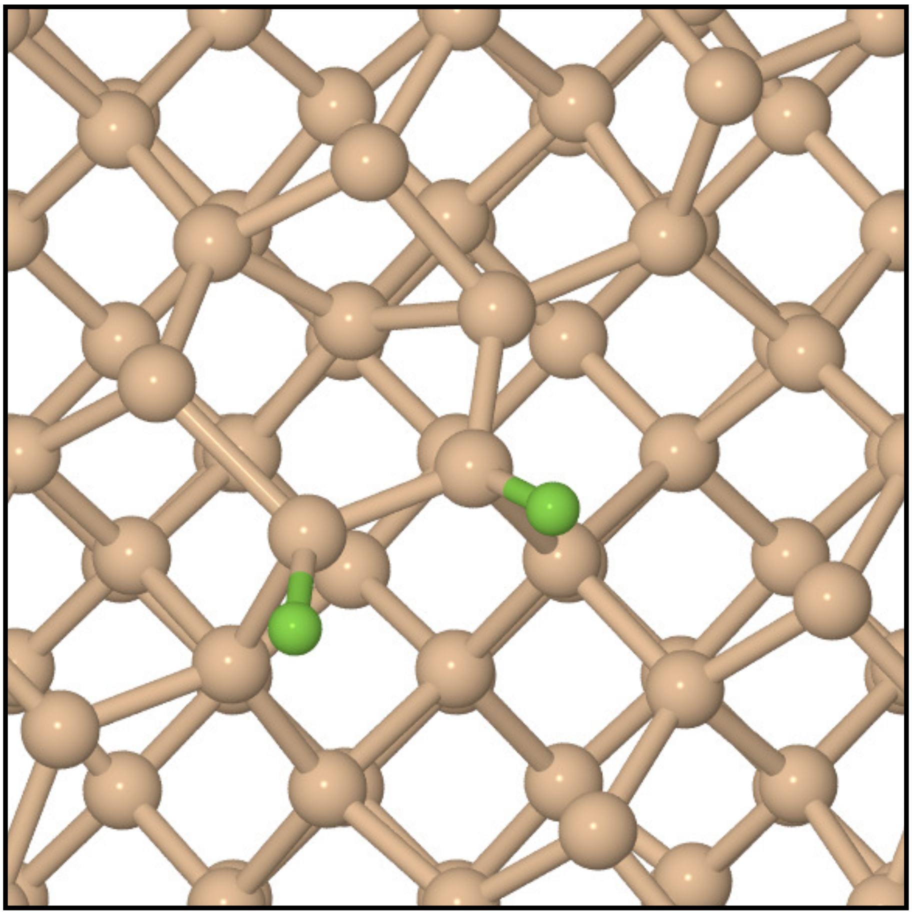

Figure S8: Geometry following adsorption on the clean Si{001} surface via the C/β trajectory.

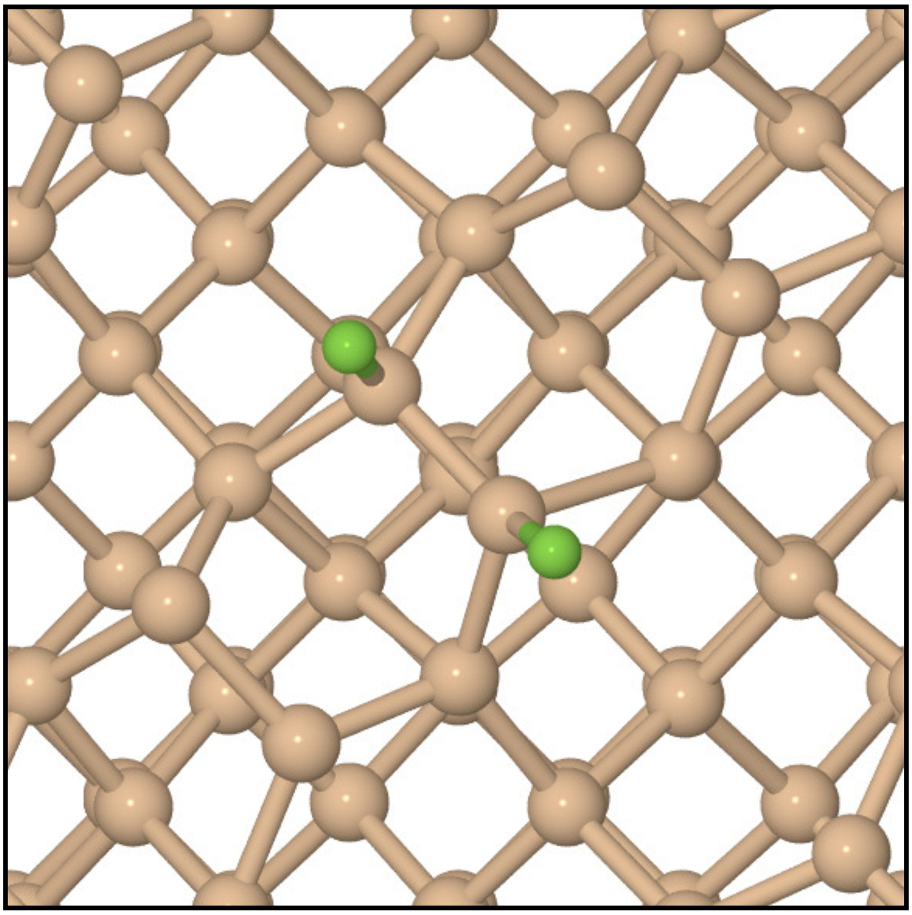

Figure S9: Geometry following adsorption on the clean Si{001} surface via the C/γ trajectory.

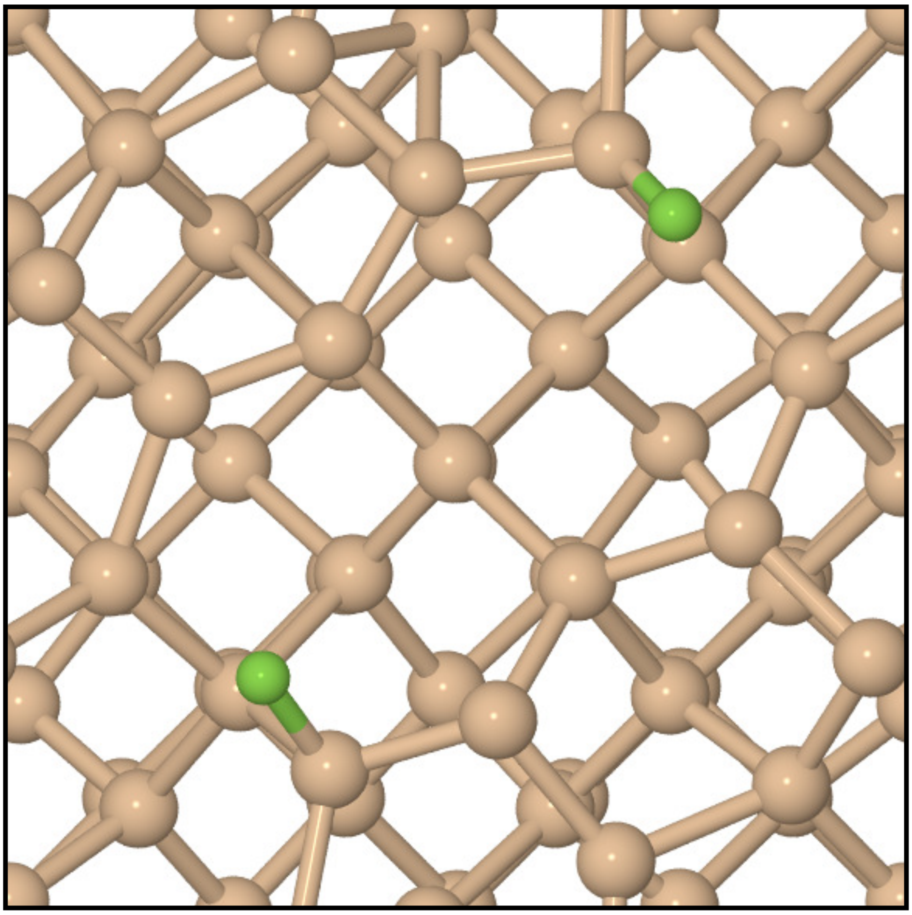

Figure S10: Geometry following adsorption on the clean Si{001} surface via the D/α trajectory.

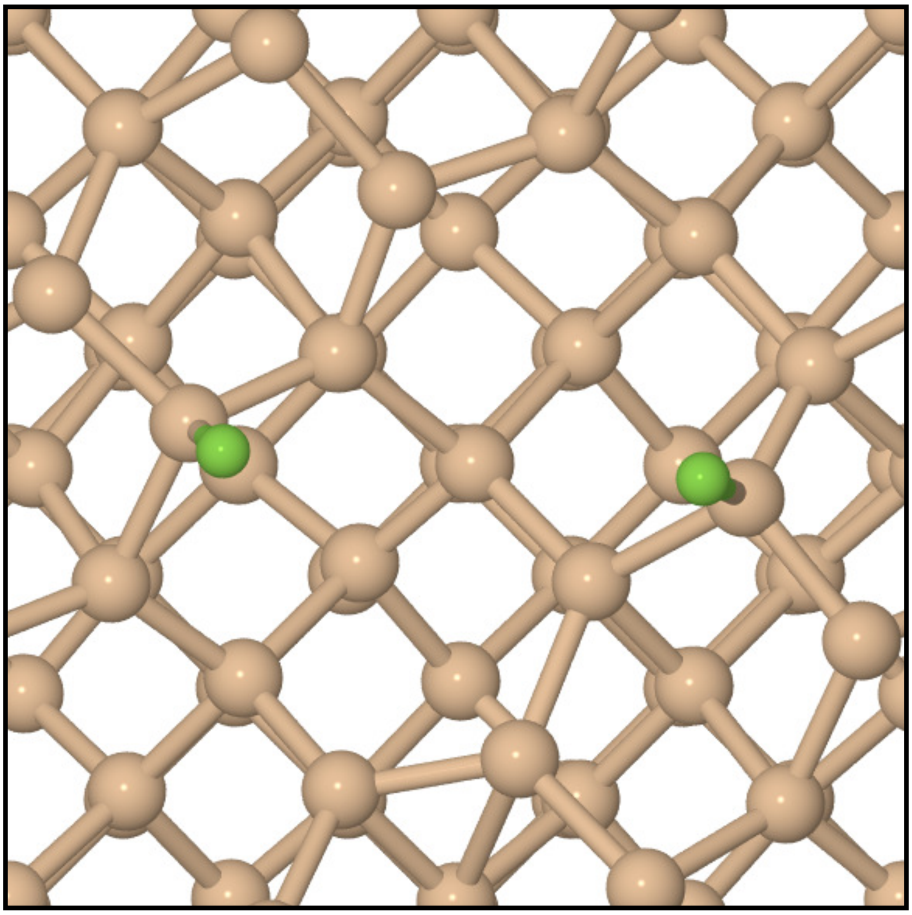

Figure S11: Geometry following adsorption on the clean Si{001} surface via the D/β trajectory.

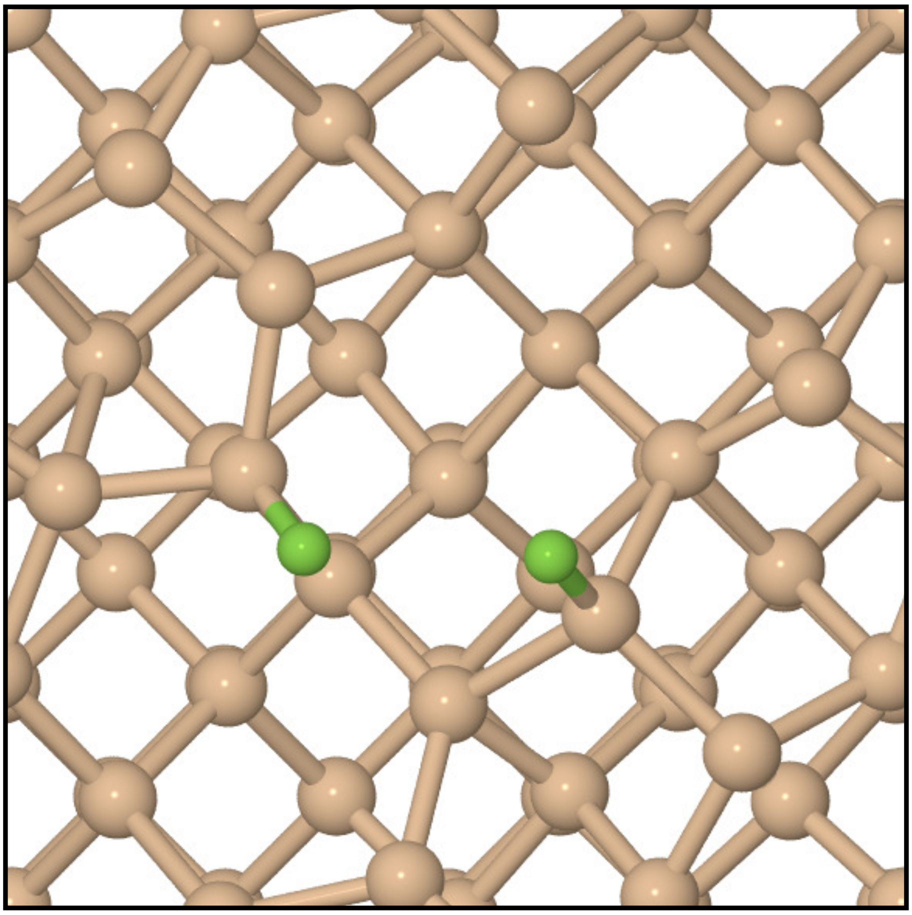

Figure S12: Geometry following adsorption on the clean Si{001} surface via the D/γ trajectory.

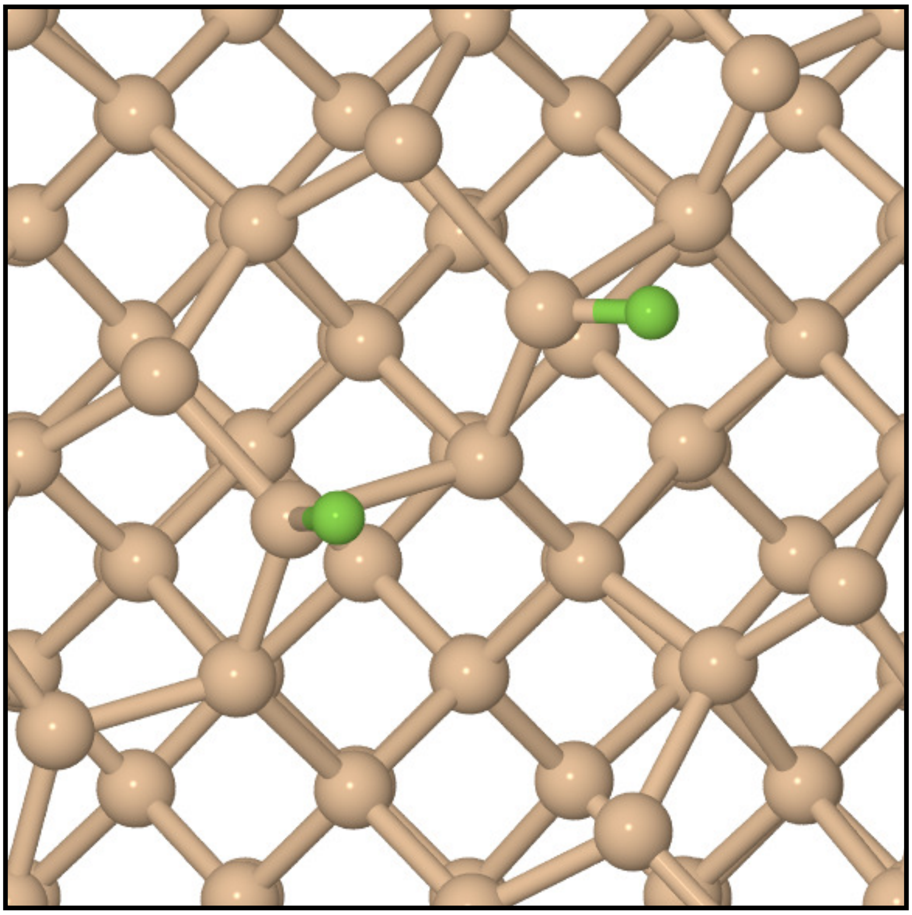

Figure S13: Geometry following adsorption on the clean Si{001} surface via the E/α trajectory.

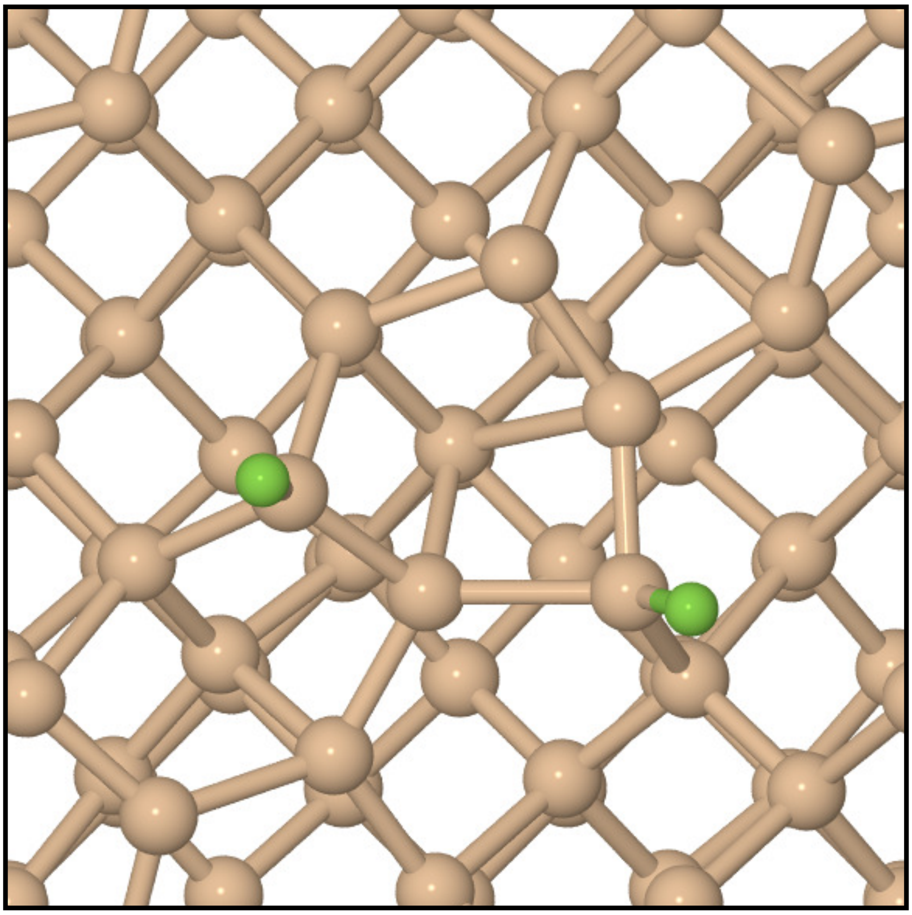

Figure S14: Geometry following adsorption on the clean Si{001} surface via the E/β trajectory.

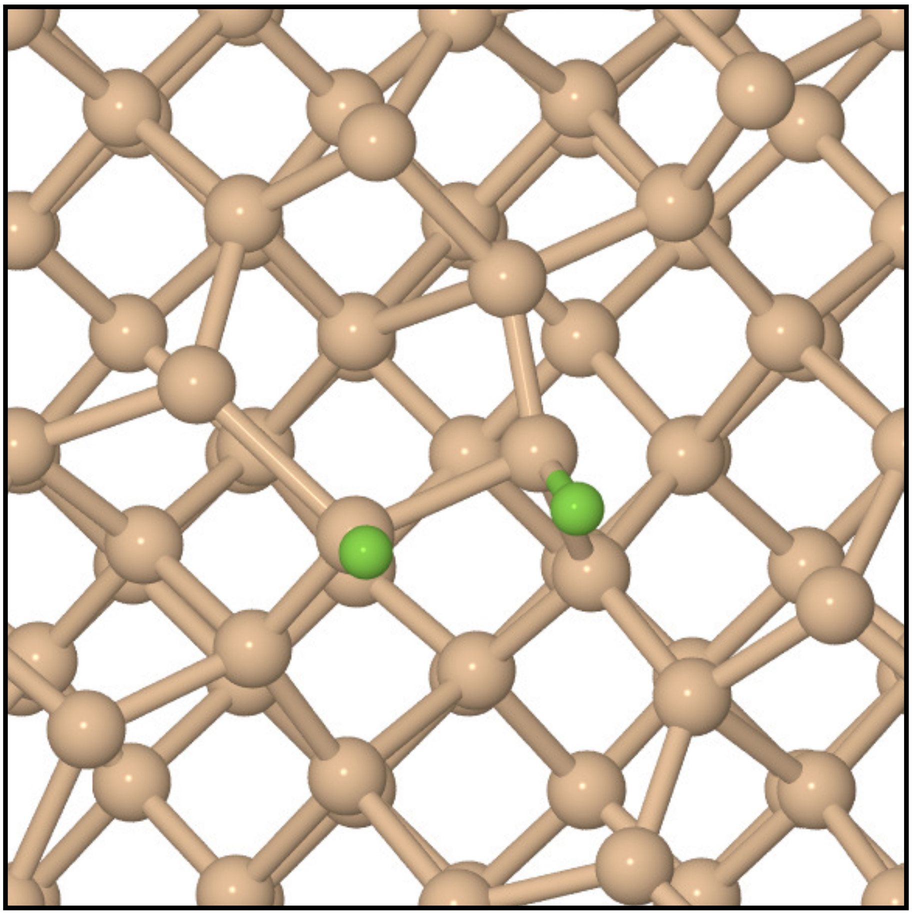

Figure S15: Geometry following adsorption on the clean Si{001} surface via the E/γ trajectory.

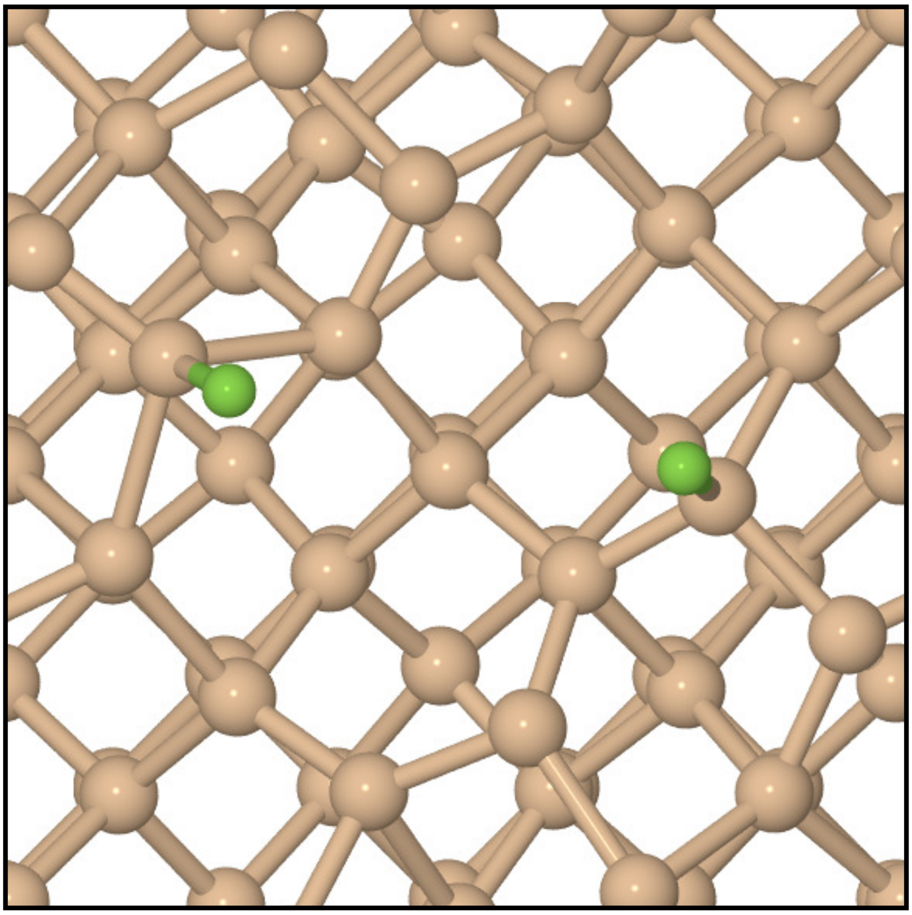

Figure S16: Geometry following adsorption on the clean Si{001} surface via the F/α trajectory.

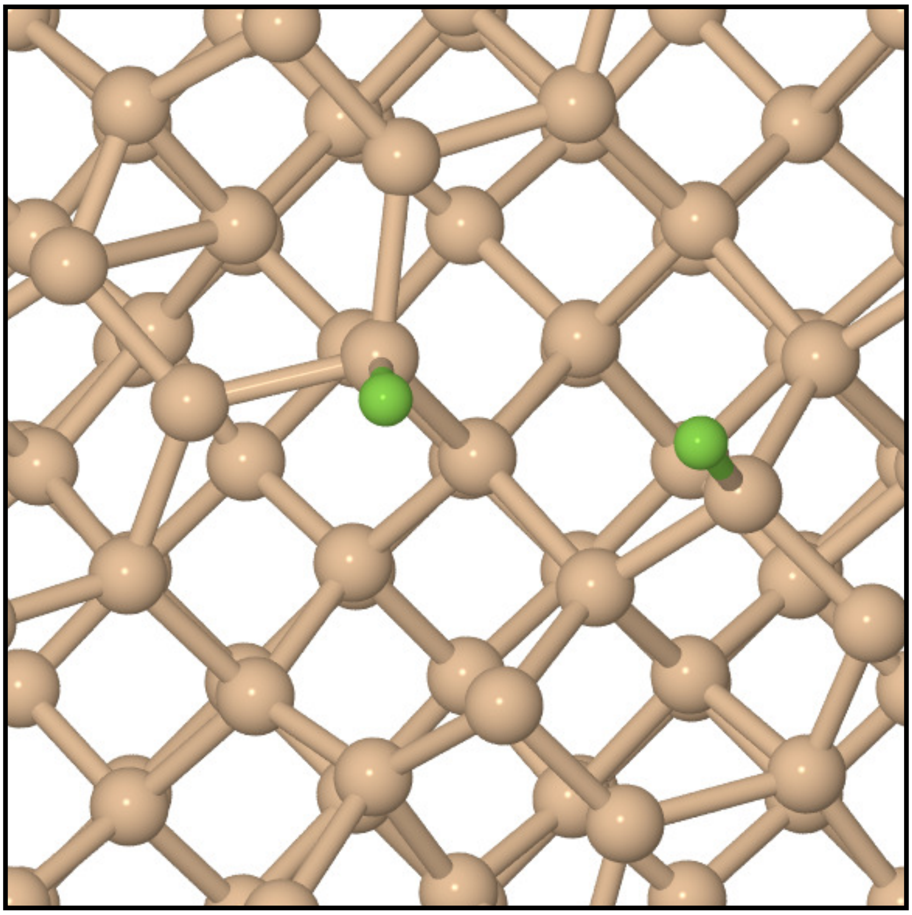

Figure S17: Geometry following adsorption on the clean Si{001} surface via the F/β trajectory.

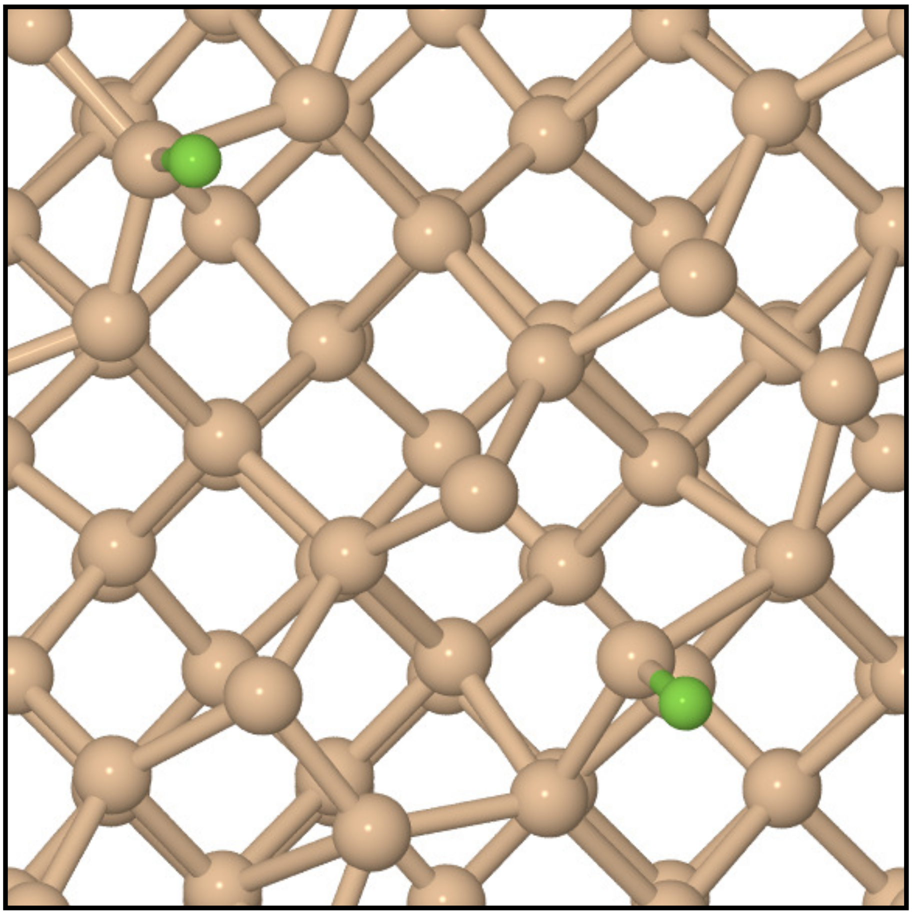

Figure S18: Geometry following adsorption on the clean Si{001} surface via the F/γ trajectory.

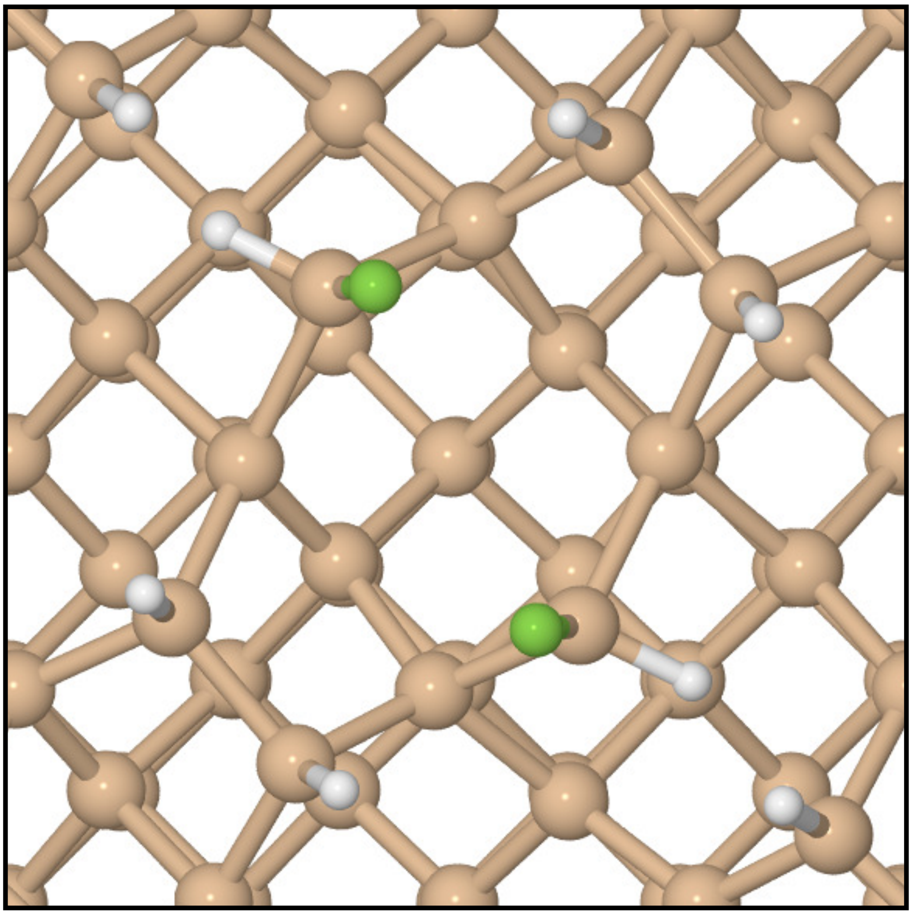

Figure S19: Geometry following adsorption on the hydrogen-passivated Si{001} surface via the A/α trajectory.

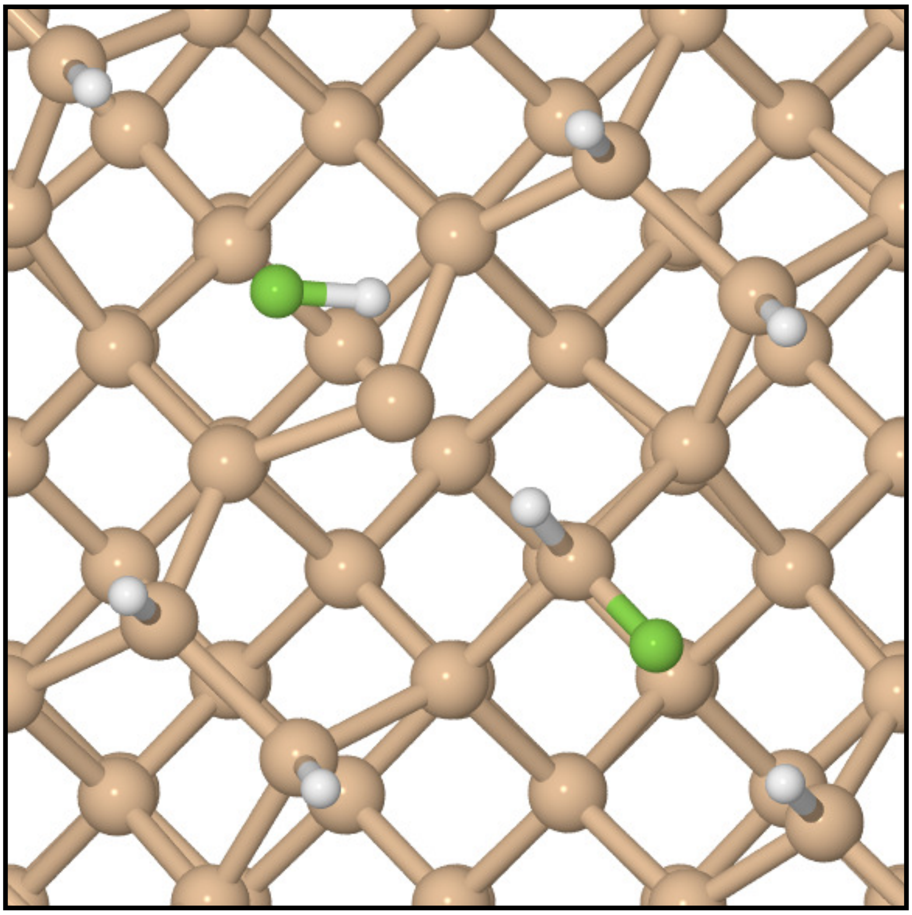

Figure S20: Geometry following adsorption on the hydrogen-passivated Si{001} surface via the A/β trajectory.

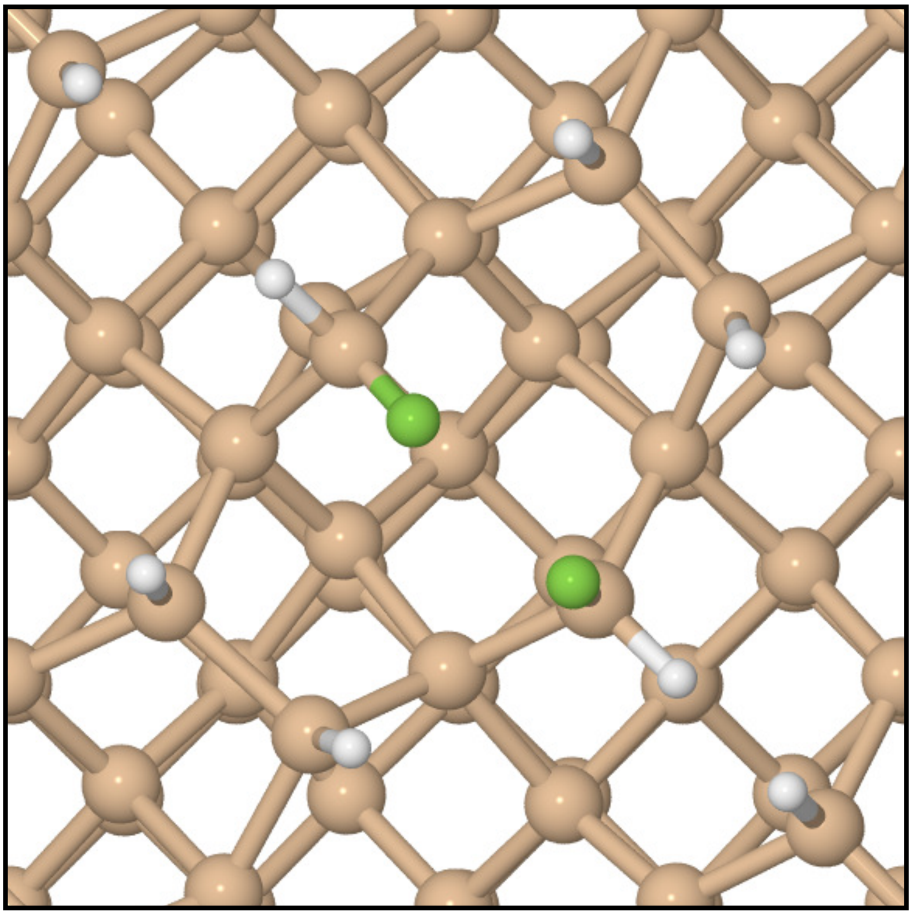

Figure S21: Geometry following adsorption on the hydrogen-passivated Si{001} surface via the A/γ trajectory.

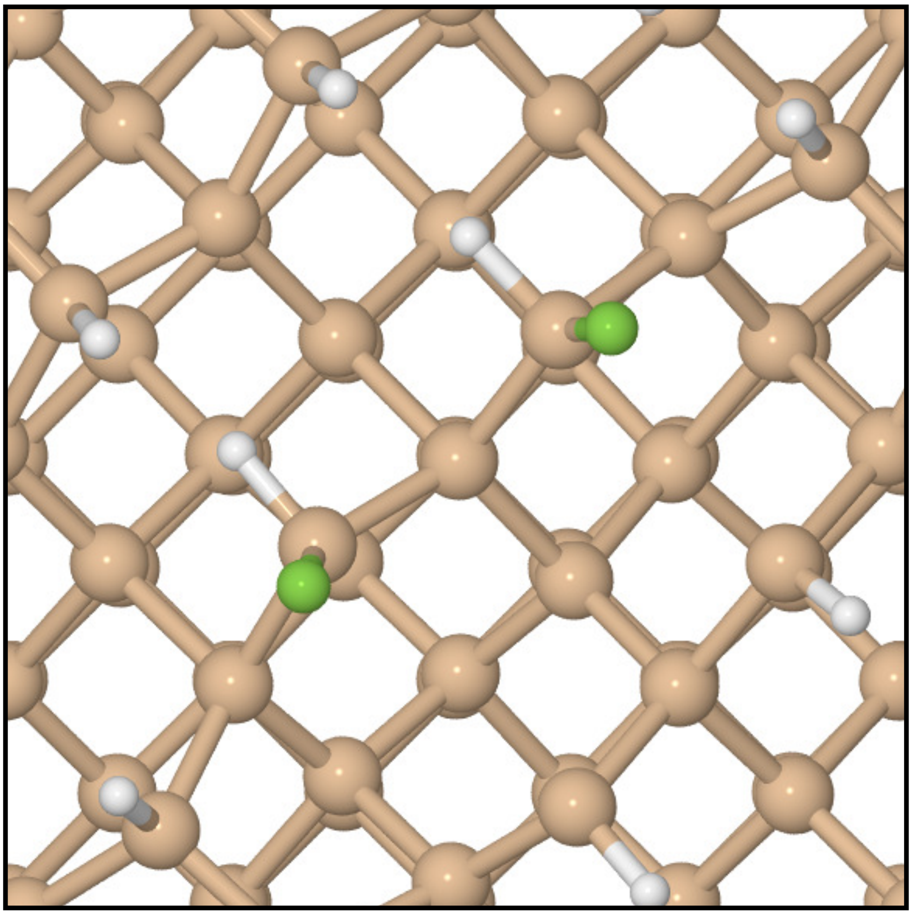

Figure S22: Geometry following adsorption on the hydrogen-passivated Si{001} surface via the B/α trajectory.

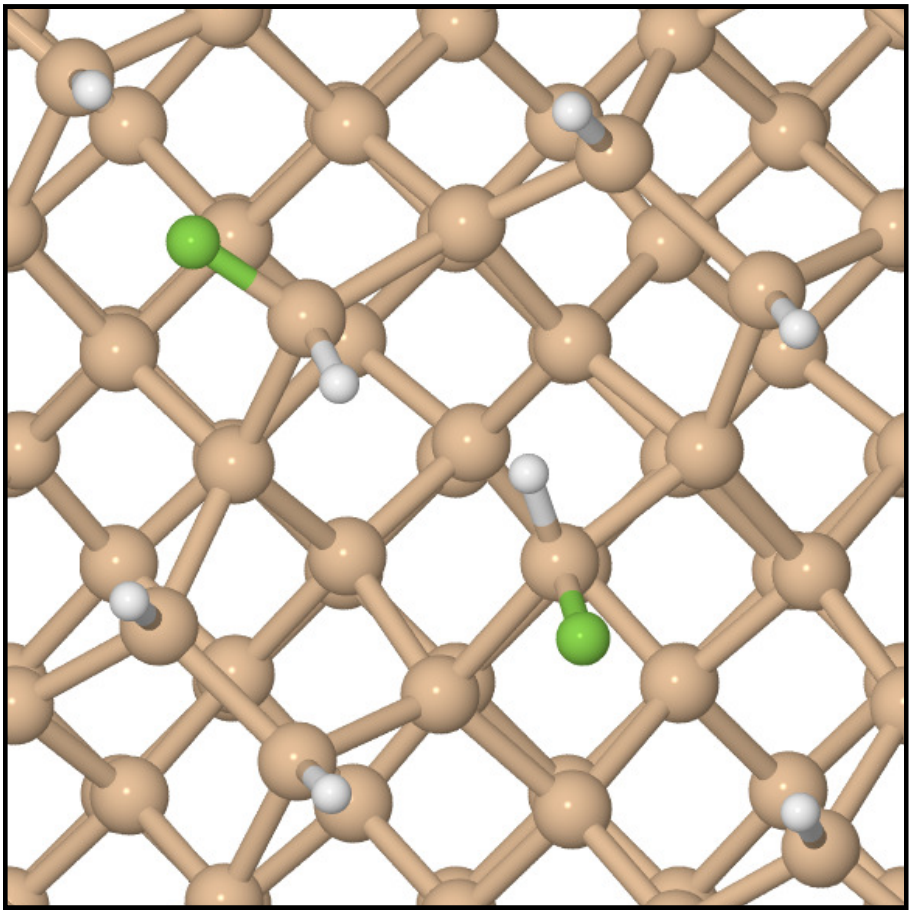

Figure S23: Geometry following adsorption on the hydrogen-passivated Si{001} surface via the B/β trajectory.

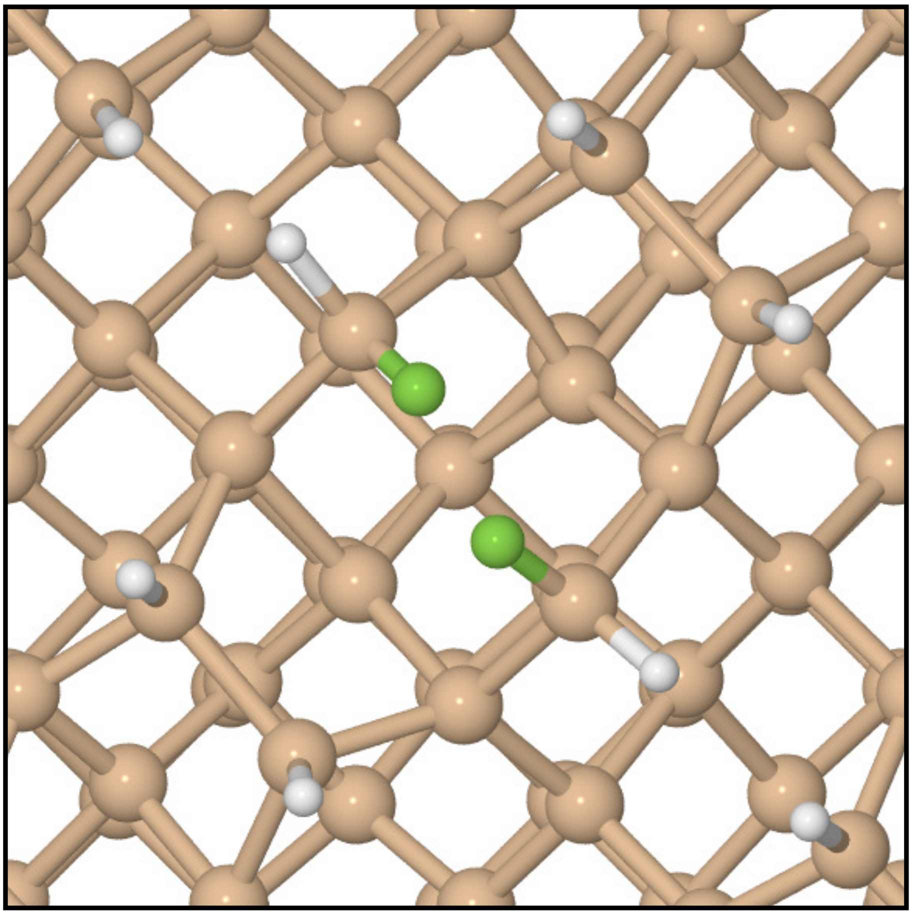

Figure S24: Geometry following adsorption on the hydrogen-passivated Si{001} surface via the B/γ trajectory.

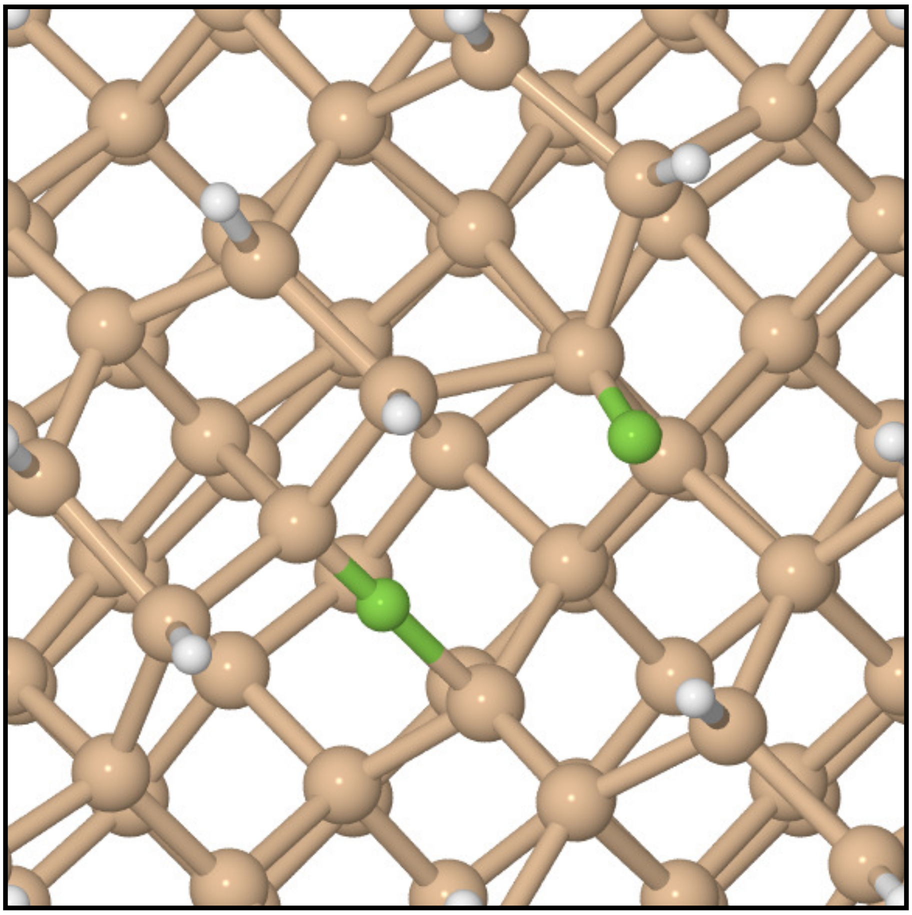

Figure S25: Geometry following adsorption on the hydrogen-passivated Si{001} surface via the C/α trajectory.

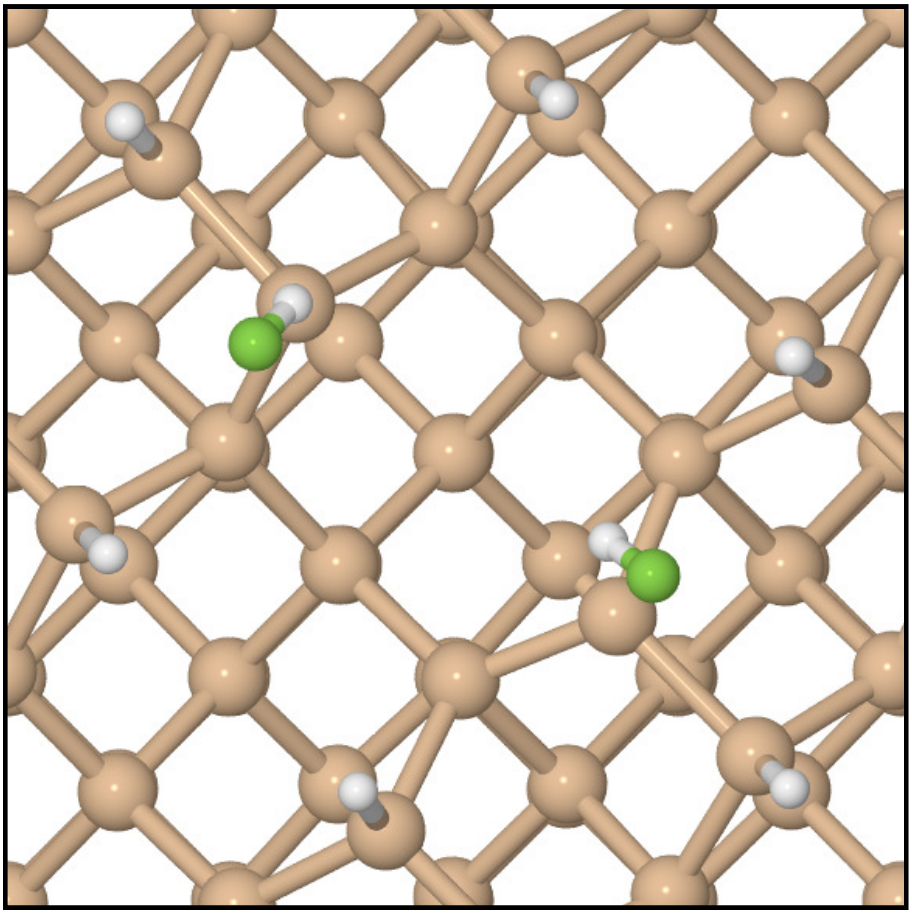

Figure S26: Geometry following adsorption on the hydrogen-passivated Si{001} surface via the C/β trajectory.

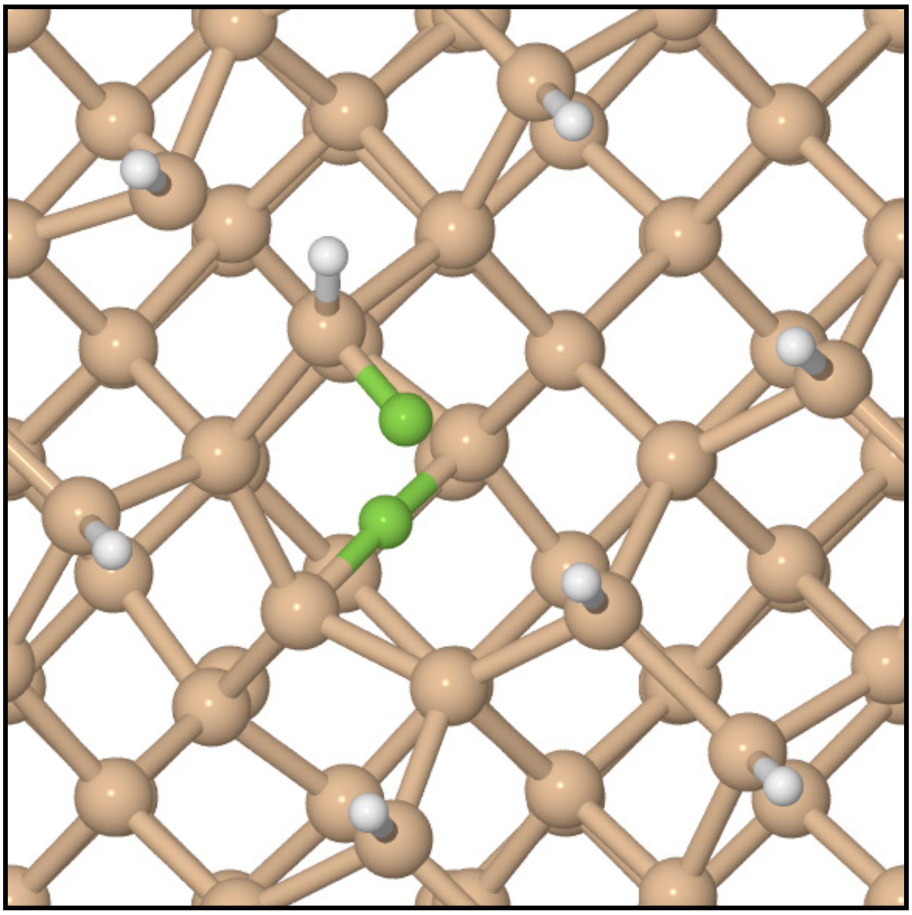

Figure S27: Geometry following adsorption on the hydrogen-passivated Si{001} surface via the C/γ trajectory.

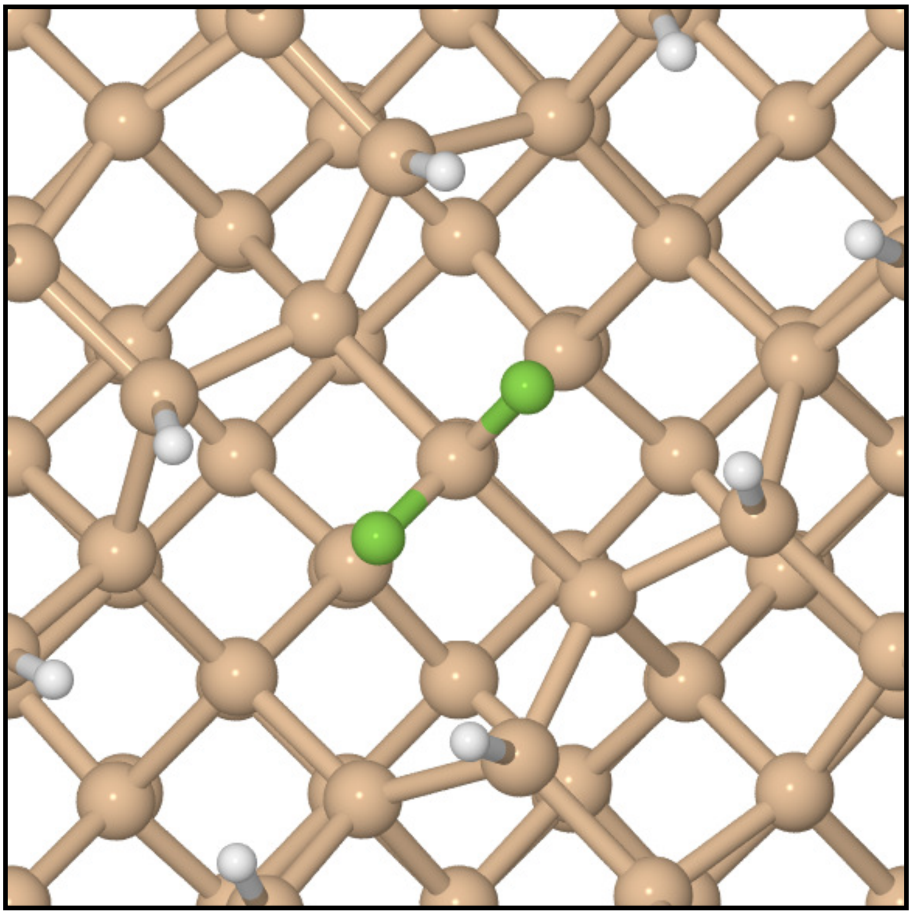

Figure S28: Geometry following adsorption on the hydrogen-passivated Si{001} surface via the D/α trajectory.

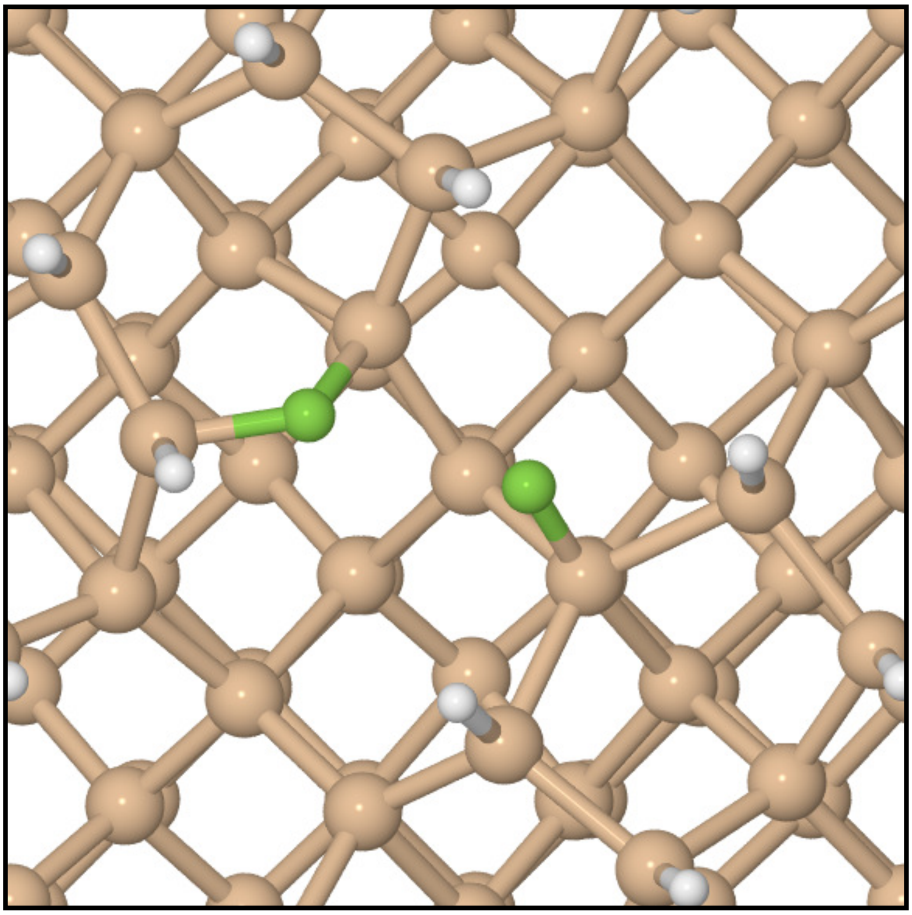

Figure S29: Geometry following adsorption on the hydrogen-passivated Si{001} surface via the D/β trajectory.

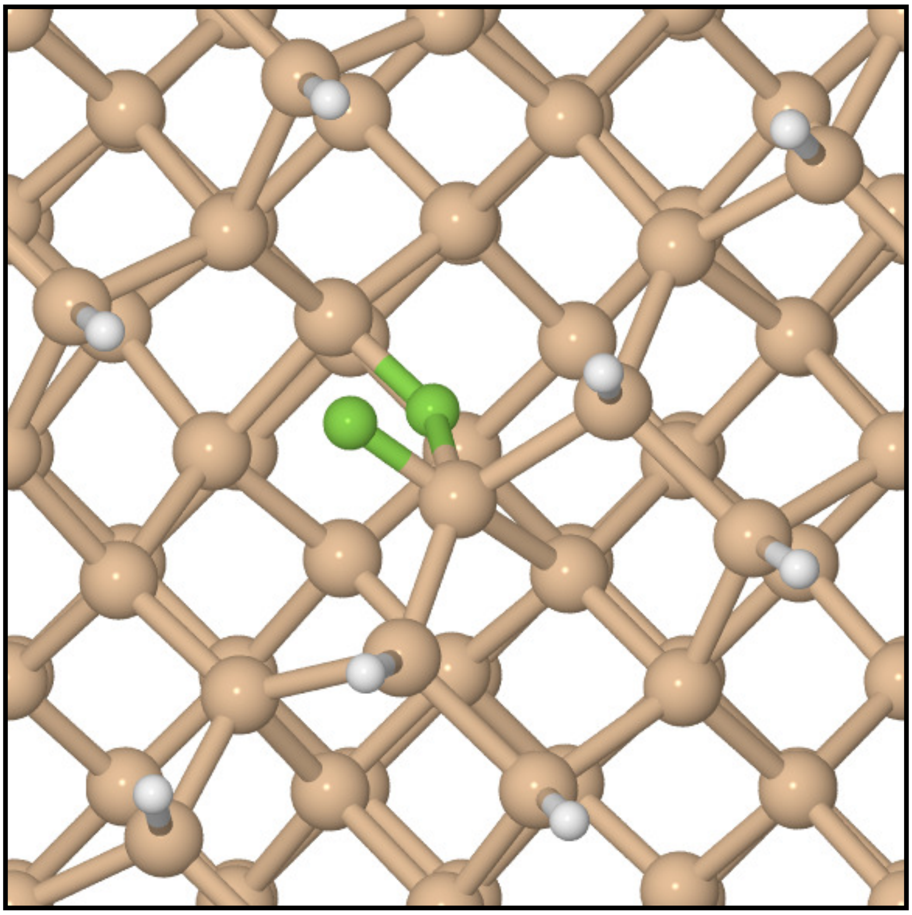

Figure S30: Geometry following adsorption on the hydrogen-passivated Si{001} surface via the D/γ trajectory.

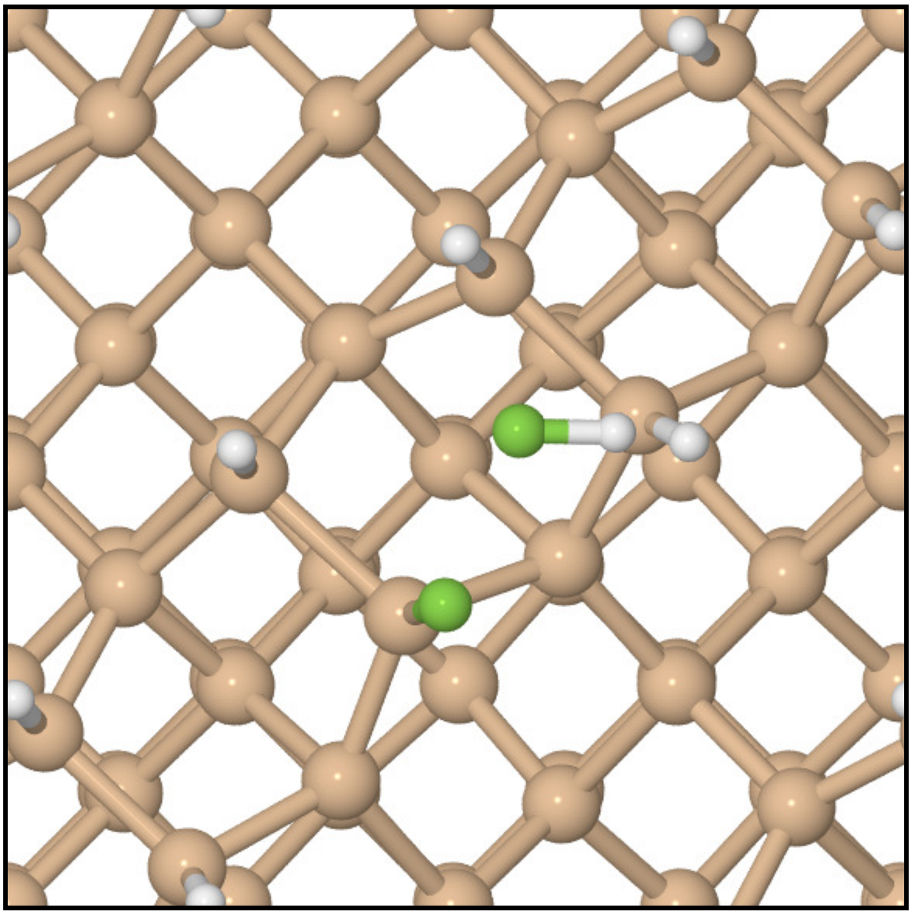

Figure S31: Geometry following adsorption on the hydrogen-passivated Si{001} surface via the E/α trajectory.

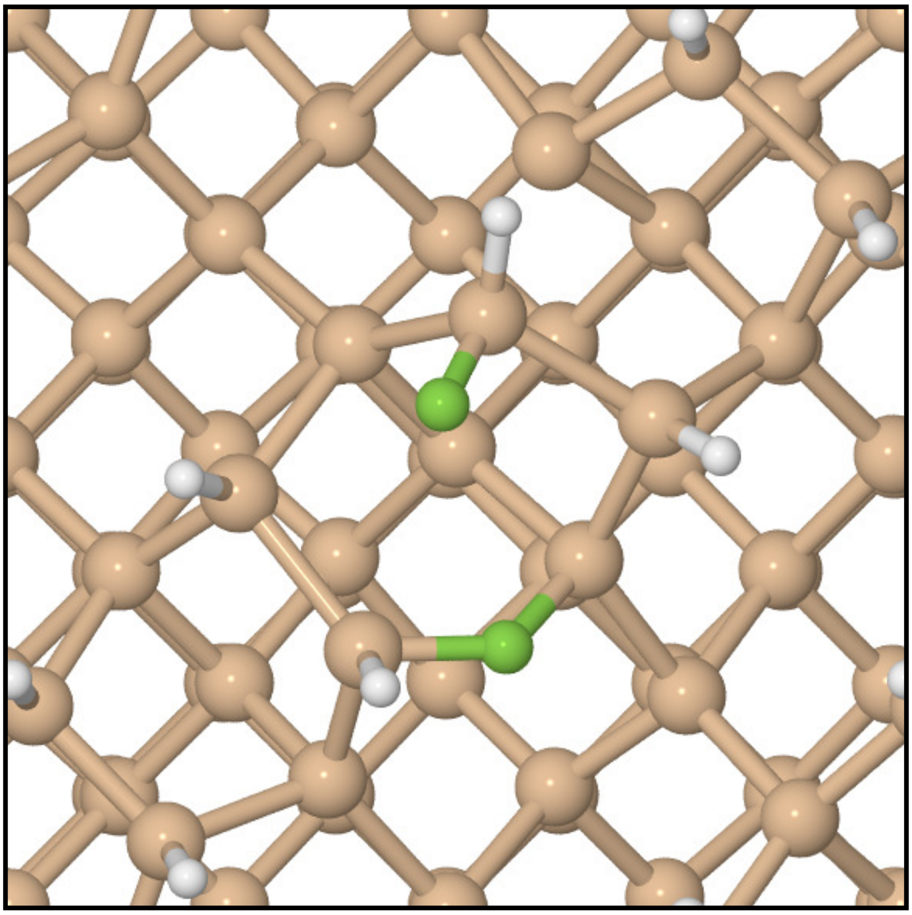

Figure S32: Geometry following adsorption on the hydrogen-passivated Si{001} surface via the E/β trajectory.

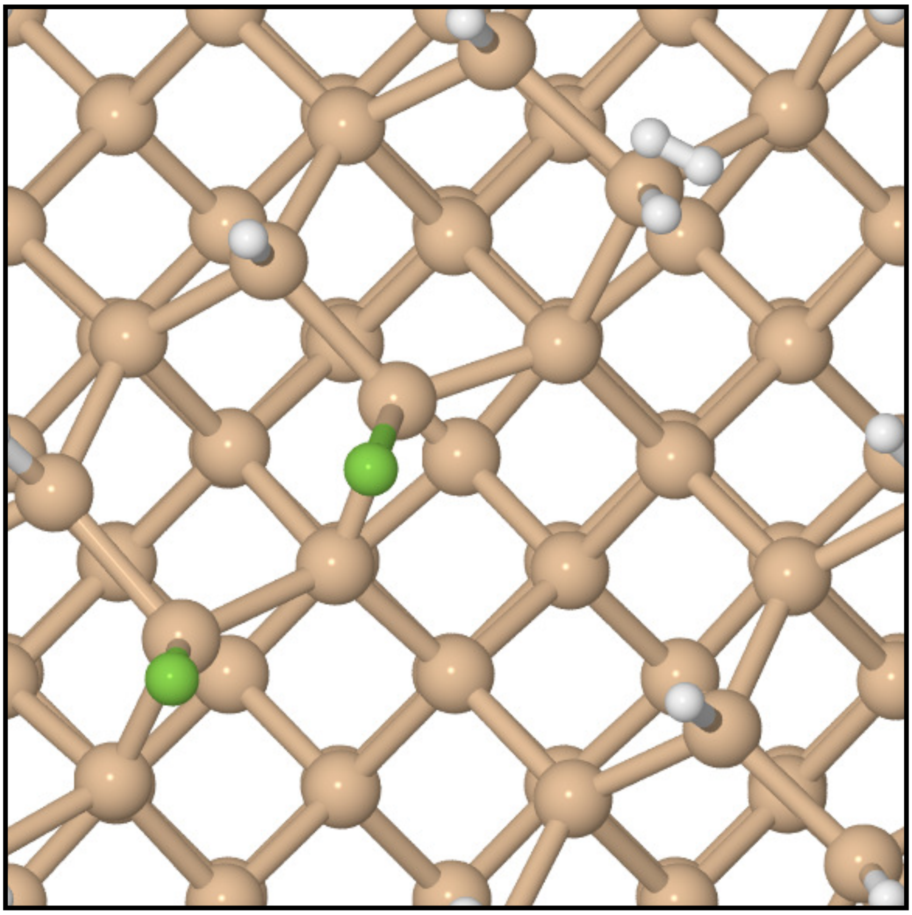

Figure S33: Geometry following adsorption on the hydrogen-passivated Si{001} surface via the E/γ trajectory.

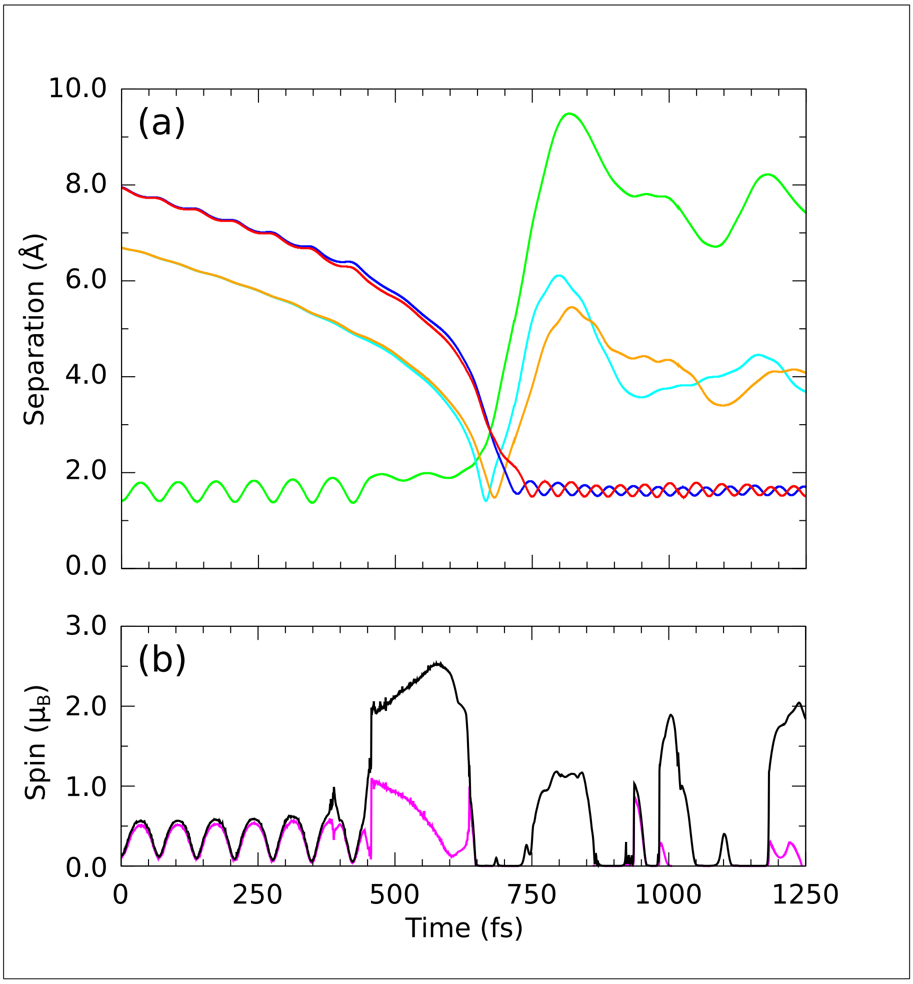

Figure S34: Bond lengths and spin traces during adsorption on the clean Si{001} surface via the A/α trajectory.

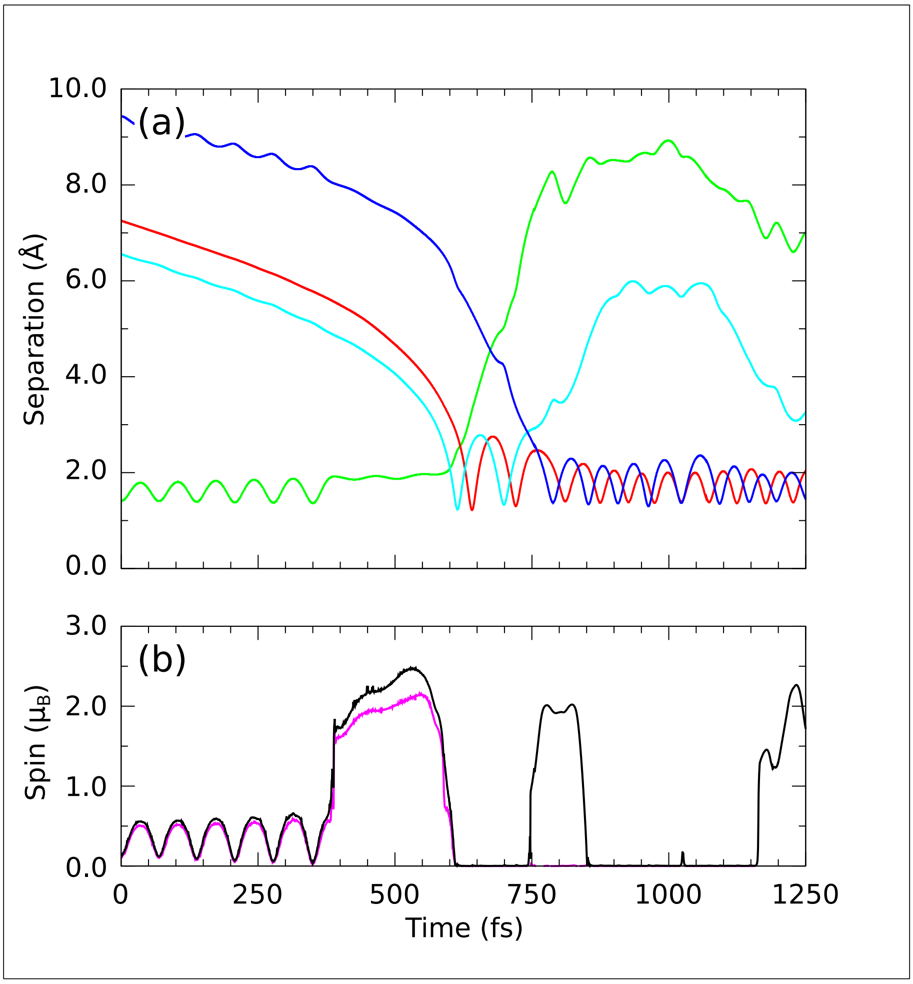

Figure S35: Bond lengths and spin traces during adsorption on the clean Si{001} surface via the A/β trajectory.

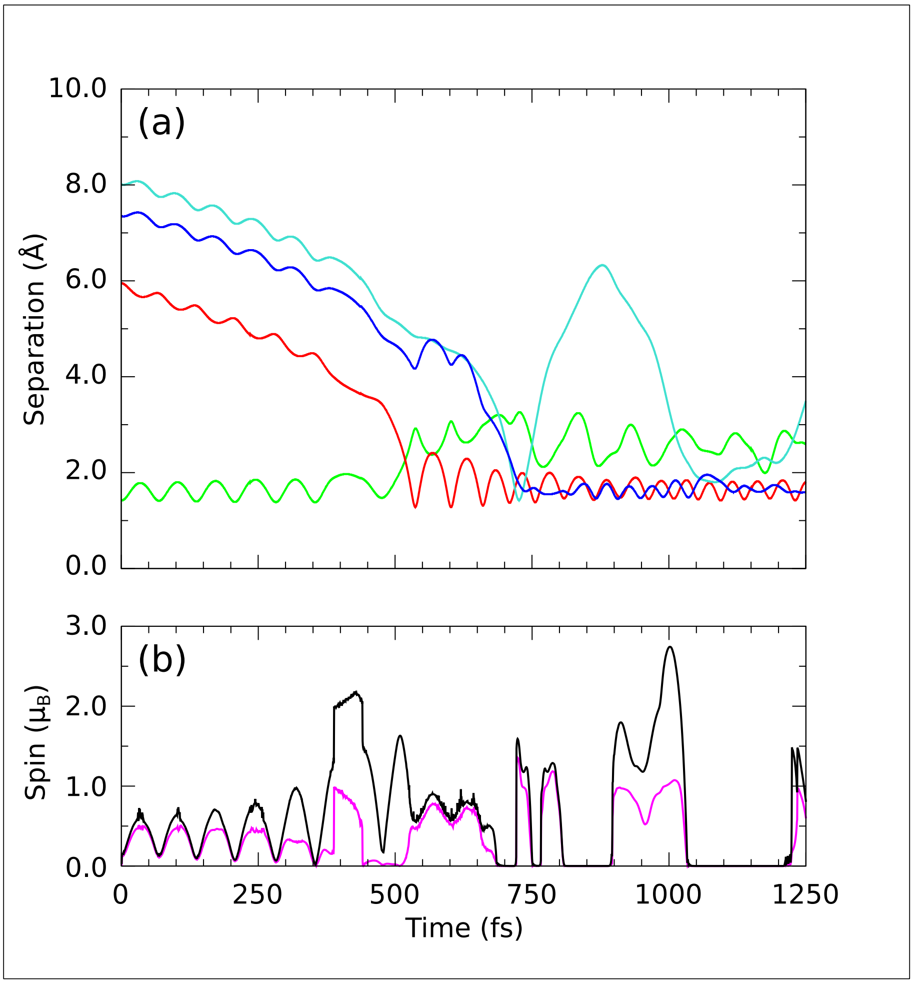

Figure S36: Bond lengths and spin traces during adsorption on the clean Si{001} surface via the A/γ trajectory.

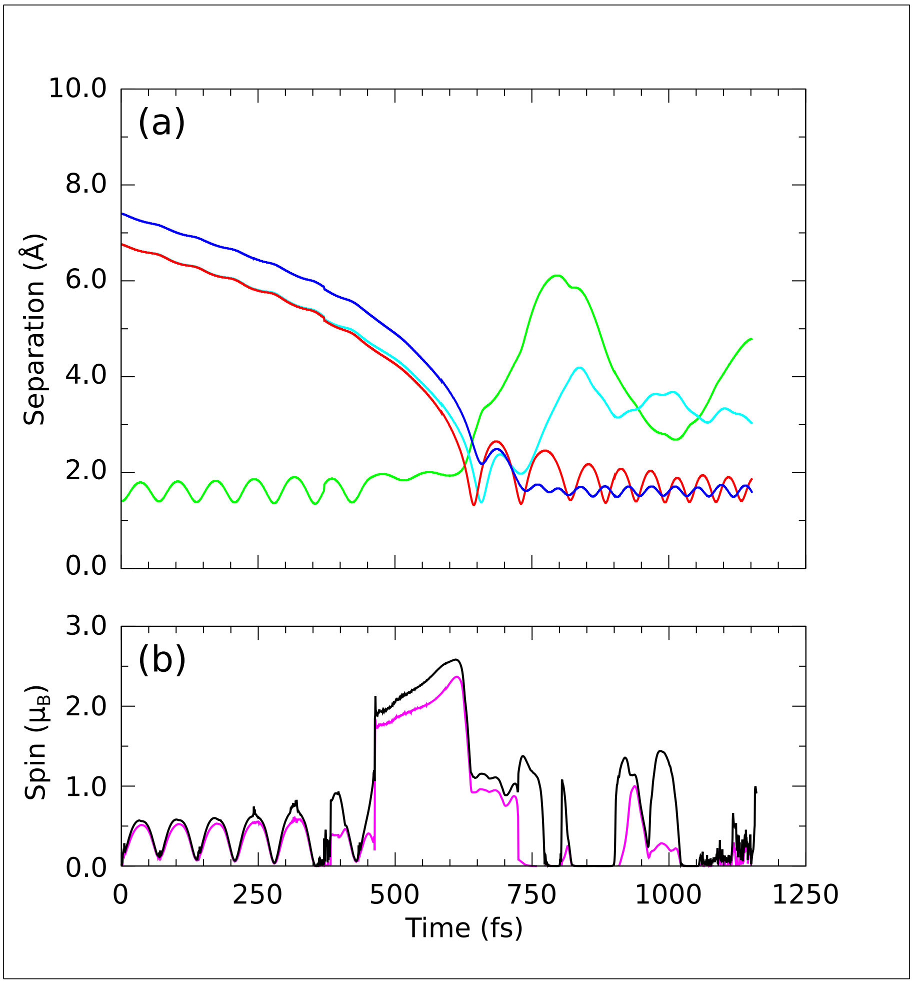

Figure S37: Bond lengths and spin traces during adsorption on the clean Si{001} surface via the B/α trajectory.

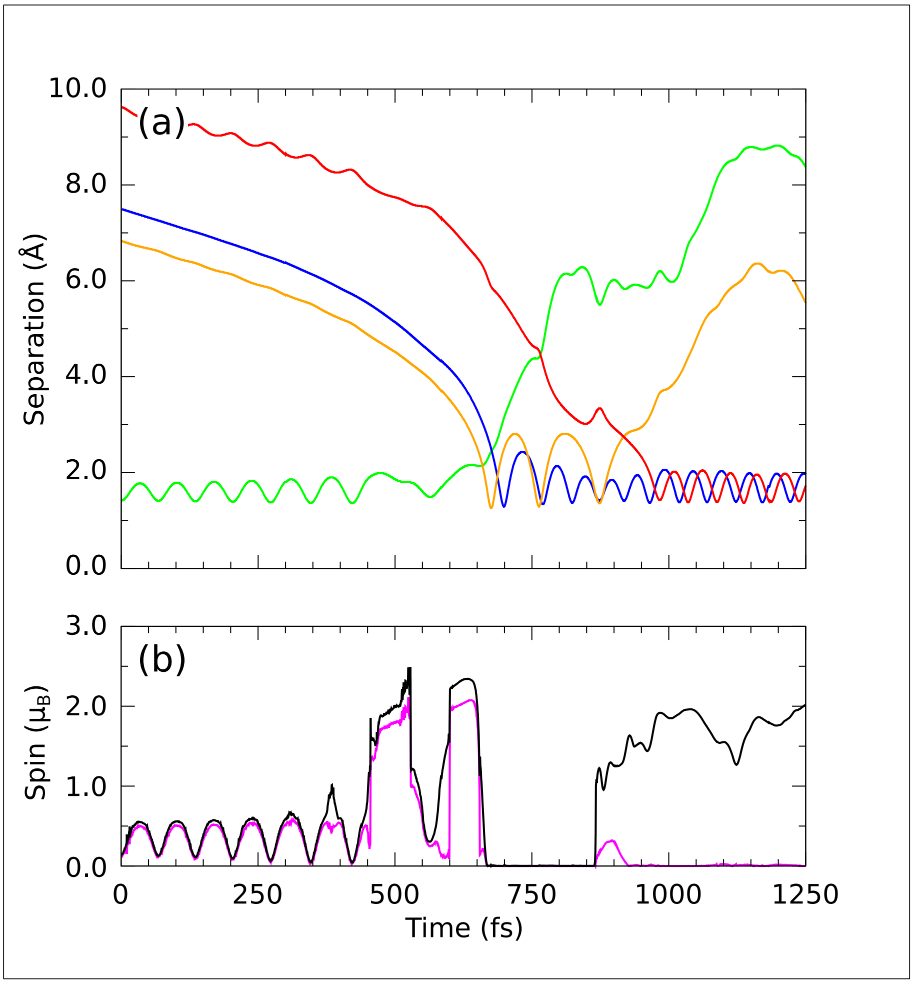

Figure S38: Bond lengths and spin traces during adsorption on the clean Si{001} surface via the B/β trajectory.

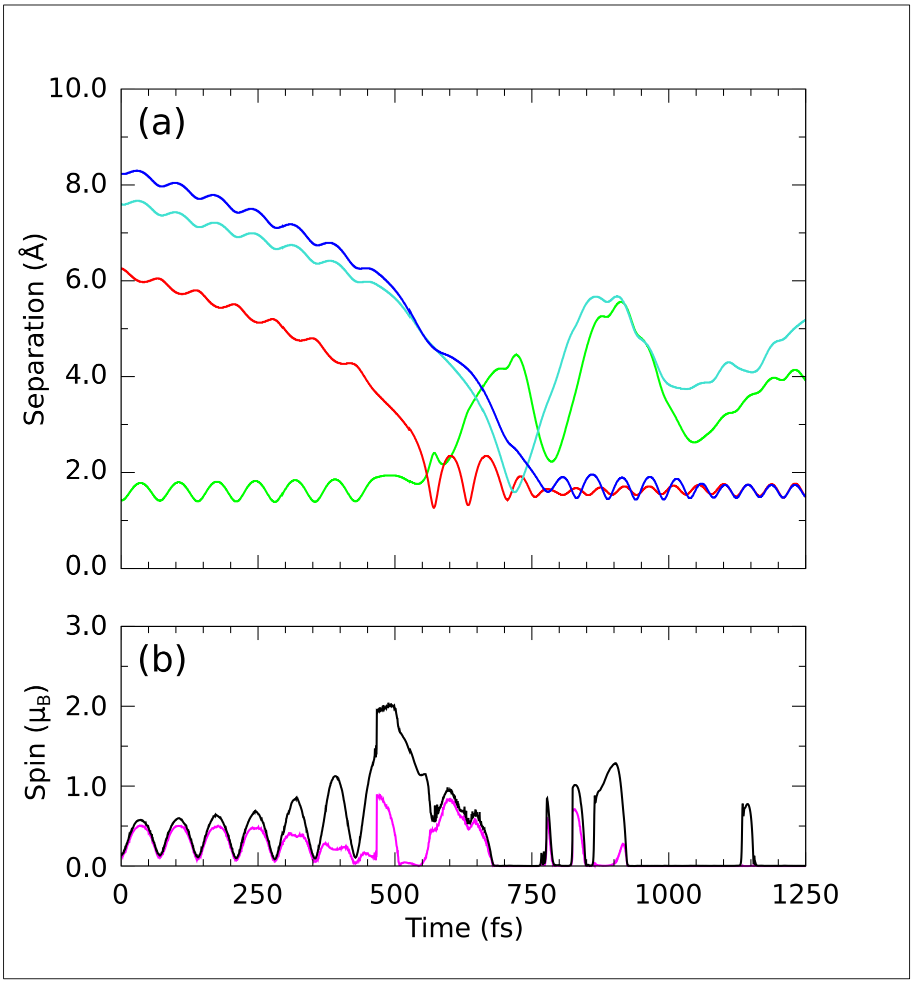

Figure S39: Bond lengths and spin traces during adsorption on the clean Si{001} surface via the B/γ trajectory.

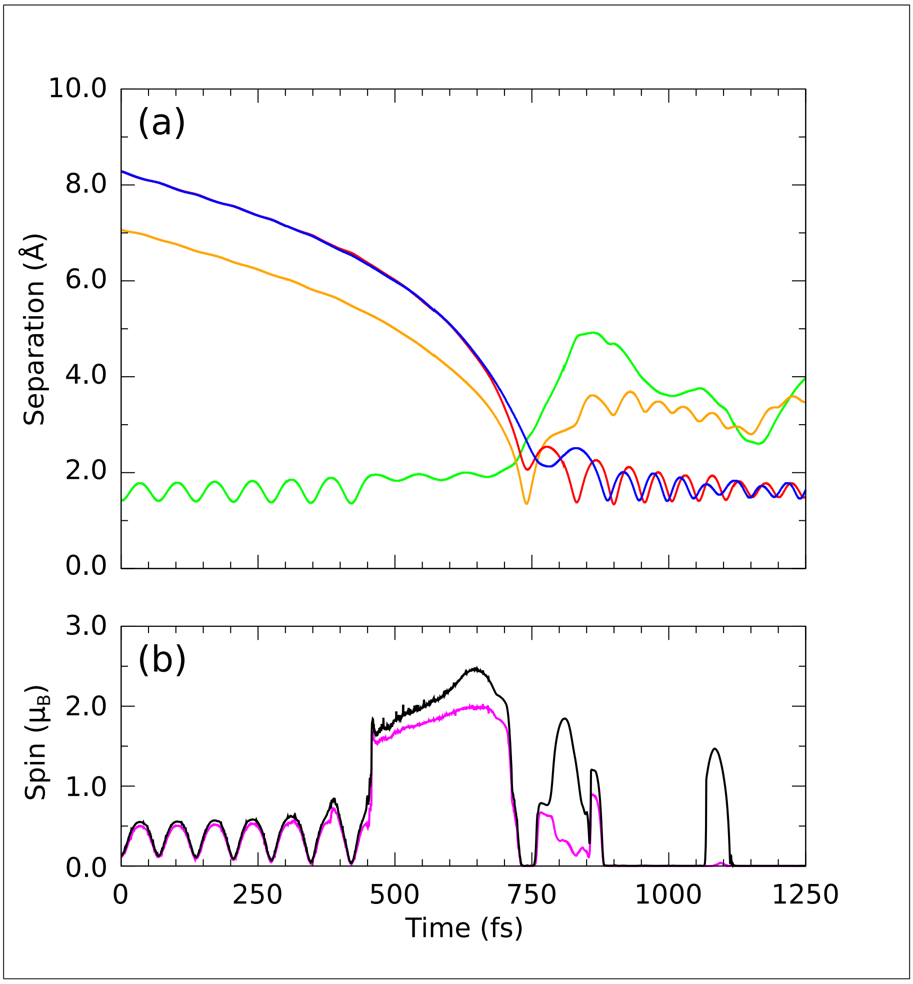

Figure S40: Bond lengths and spin traces during adsorption on the clean Si{001} surface via the C/α trajectory.

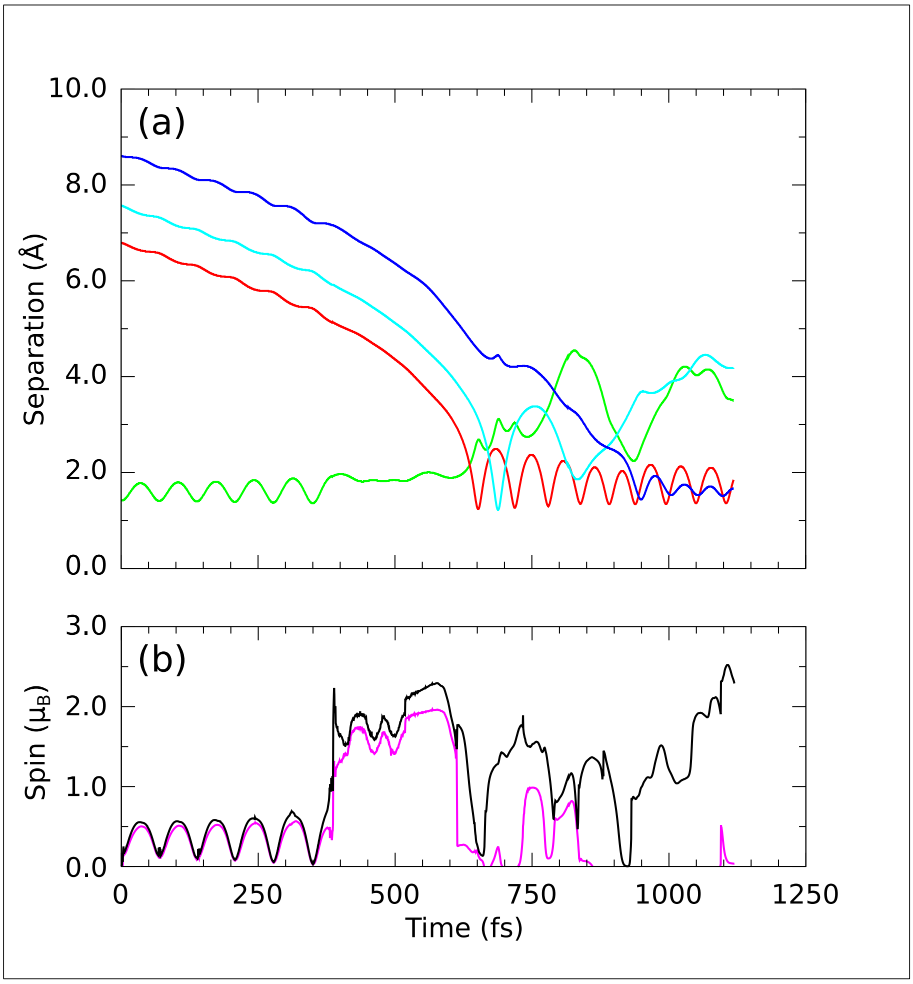

Figure S41: Bond lengths and spin traces during adsorption on the clean Si{001} surface via the C/β trajectory.

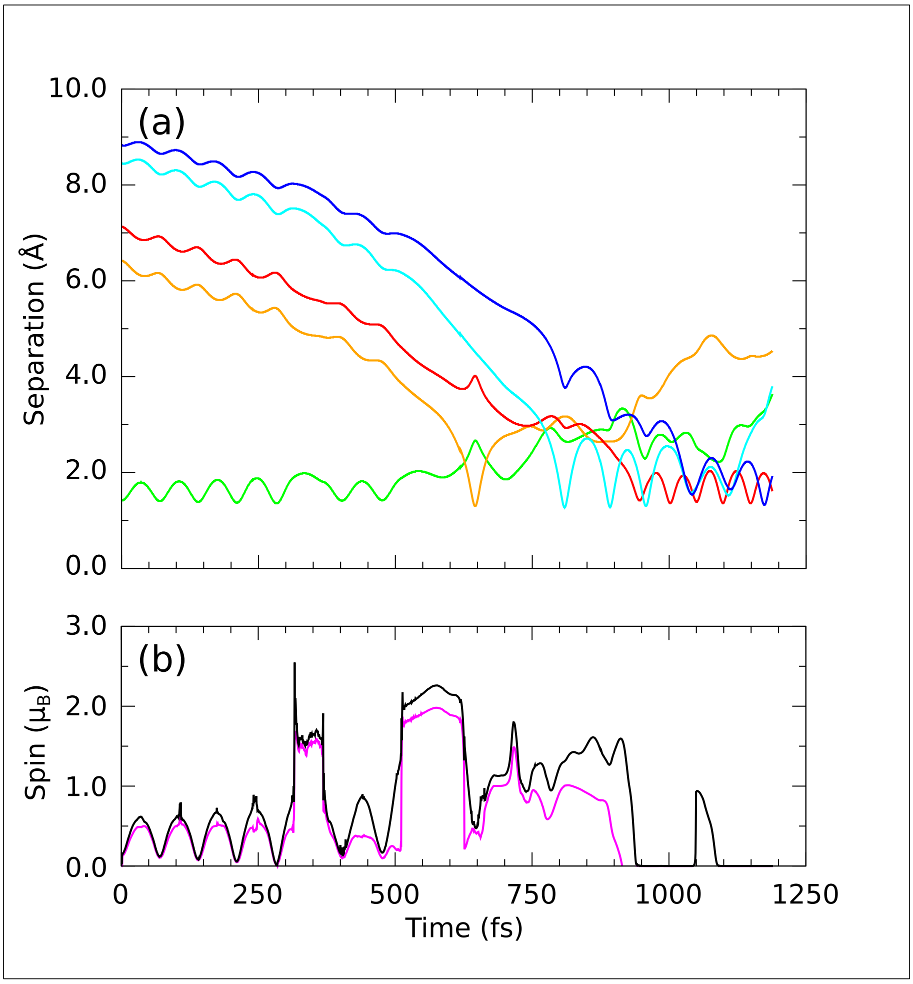

Figure S42: Bond lengths and spin traces during adsorption on the clean Si{001} surface via the C/γ trajectory.

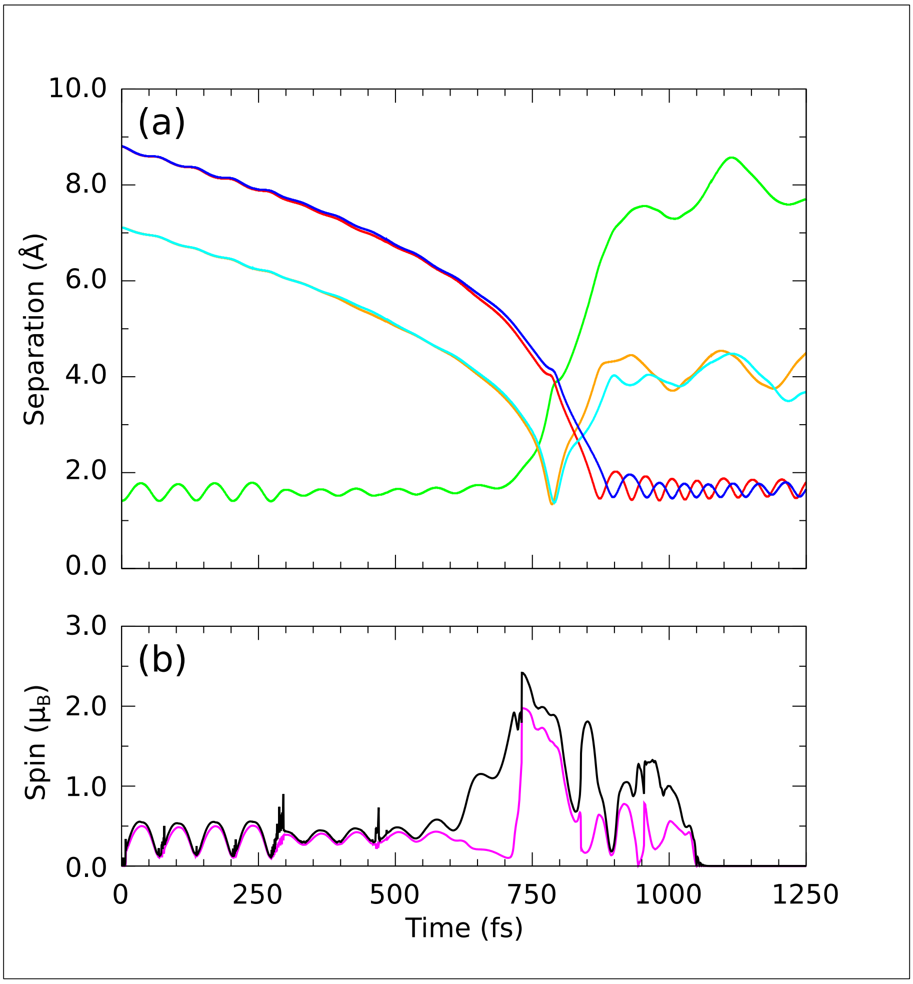

Figure S43: Bond lengths and spin traces during adsorption on the clean Si{001} surface via the D/α trajectory.

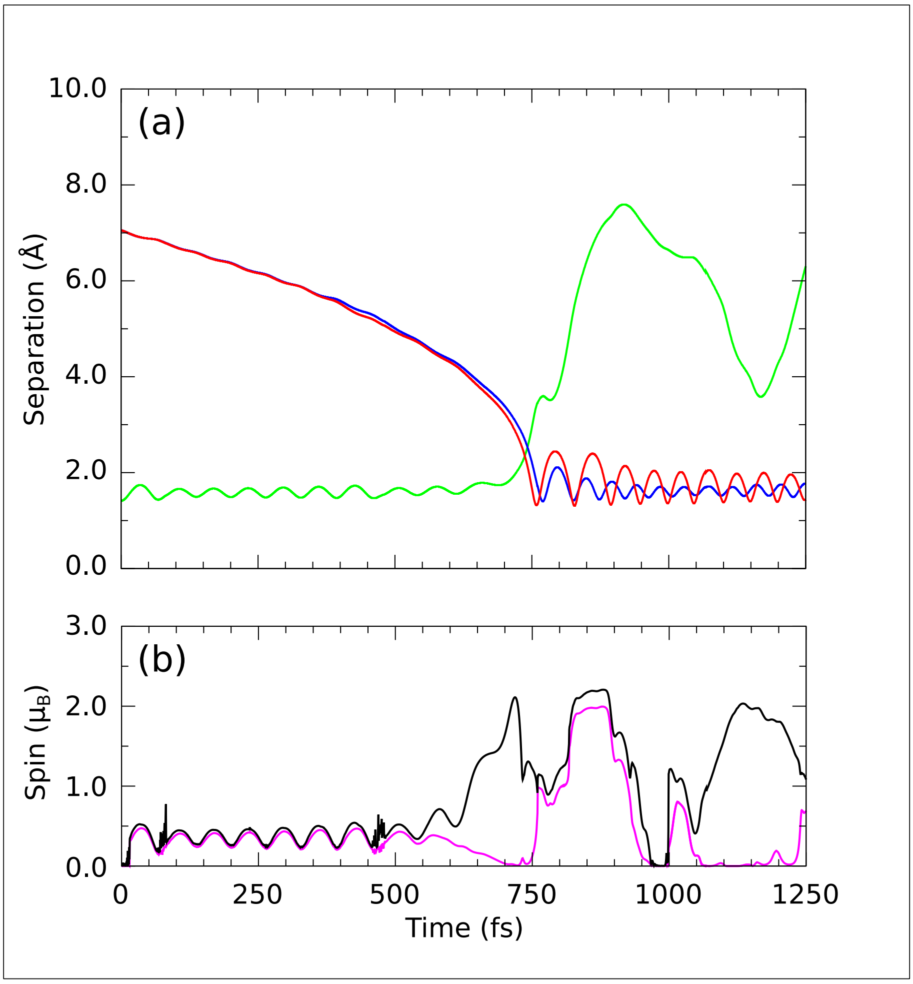

Figure S44: Bond lengths and spin traces during adsorption on the clean Si{001} surface via the D/β trajectory.

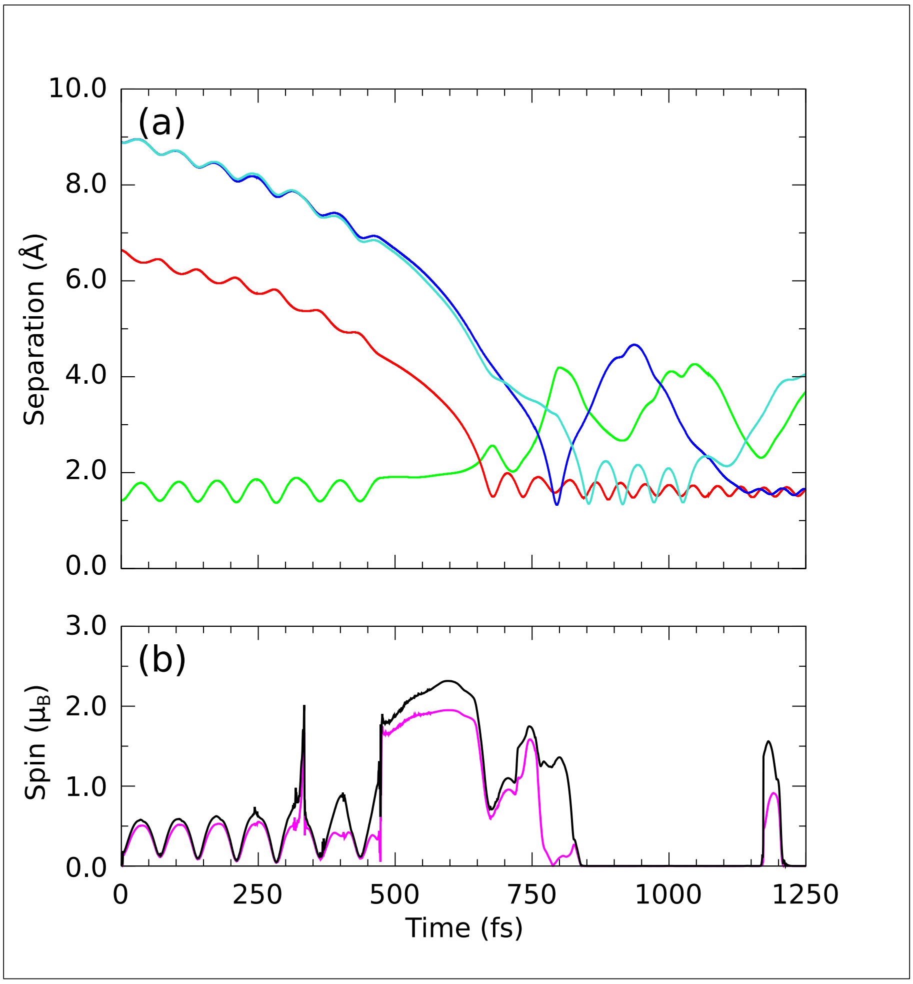

Figure S45: Bond lengths and spin traces during adsorption on the clean Si{001} surface via the D/γ trajectory.

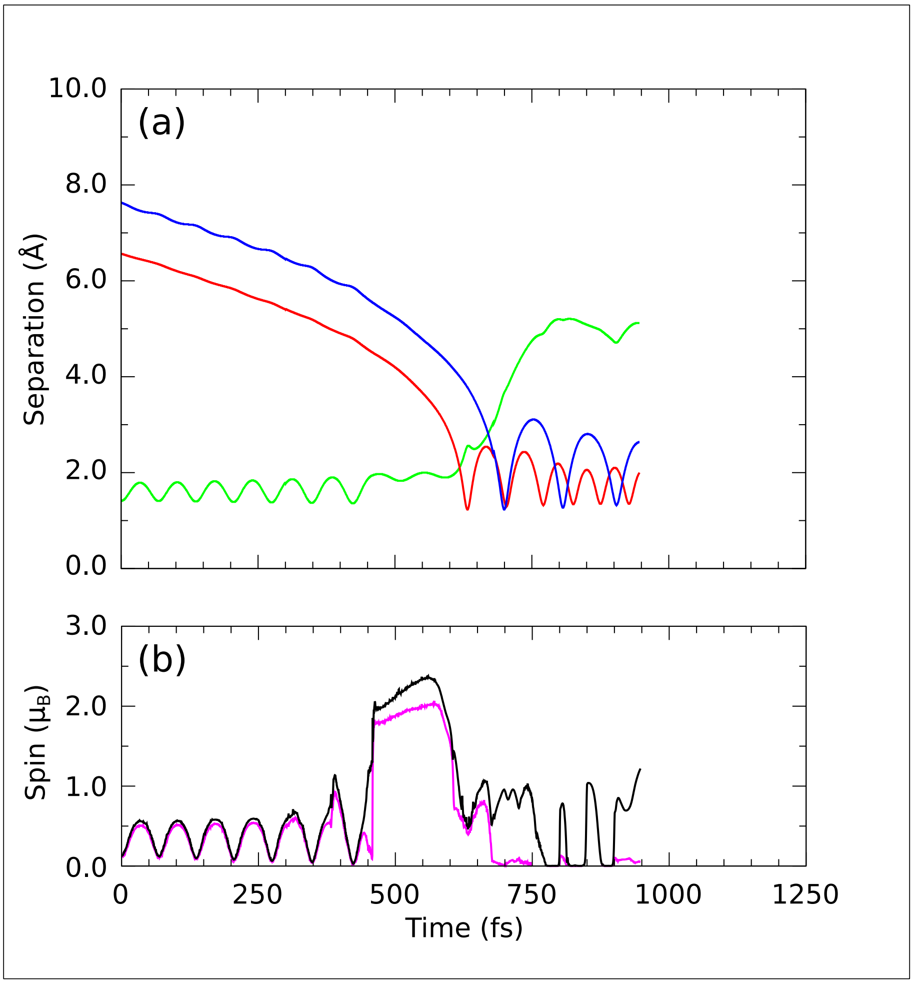

Figure S46: Bond lengths and spin traces during adsorption on the clean Si{001} surface via the E/α trajectory.

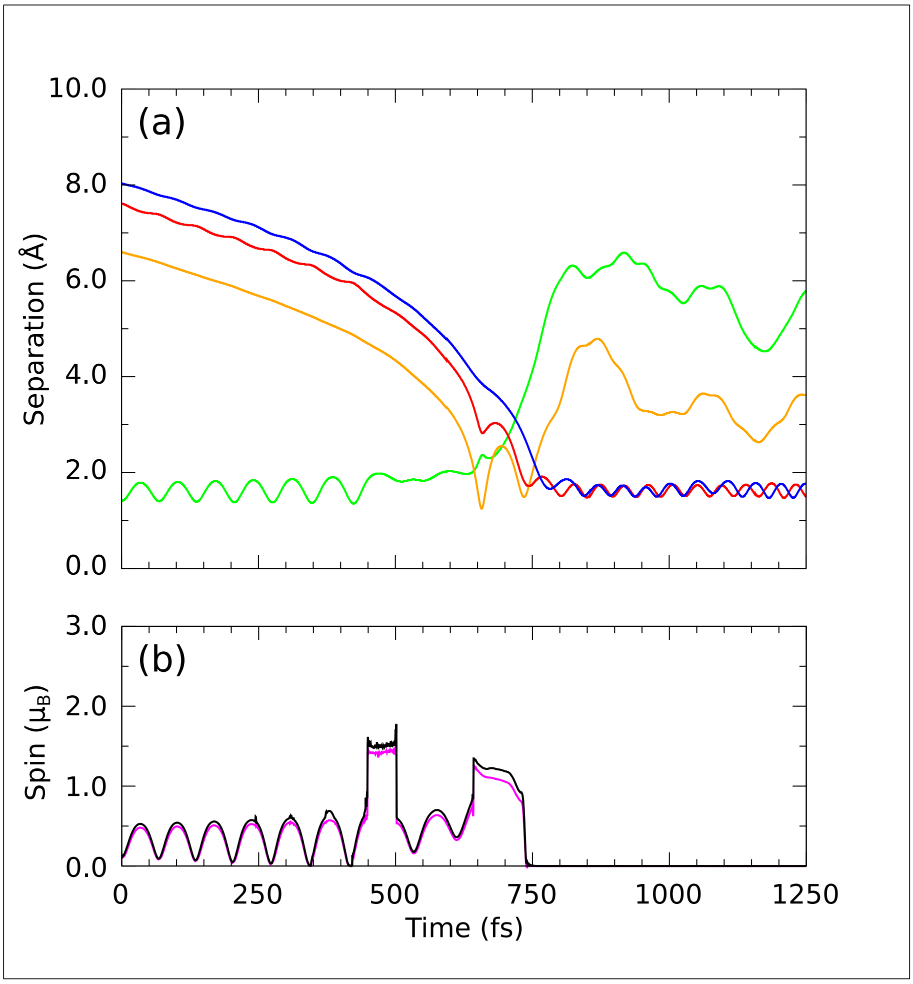

Figure S47: Bond lengths and spin traces during adsorption on the clean Si{001} surface via the E/β trajectory.

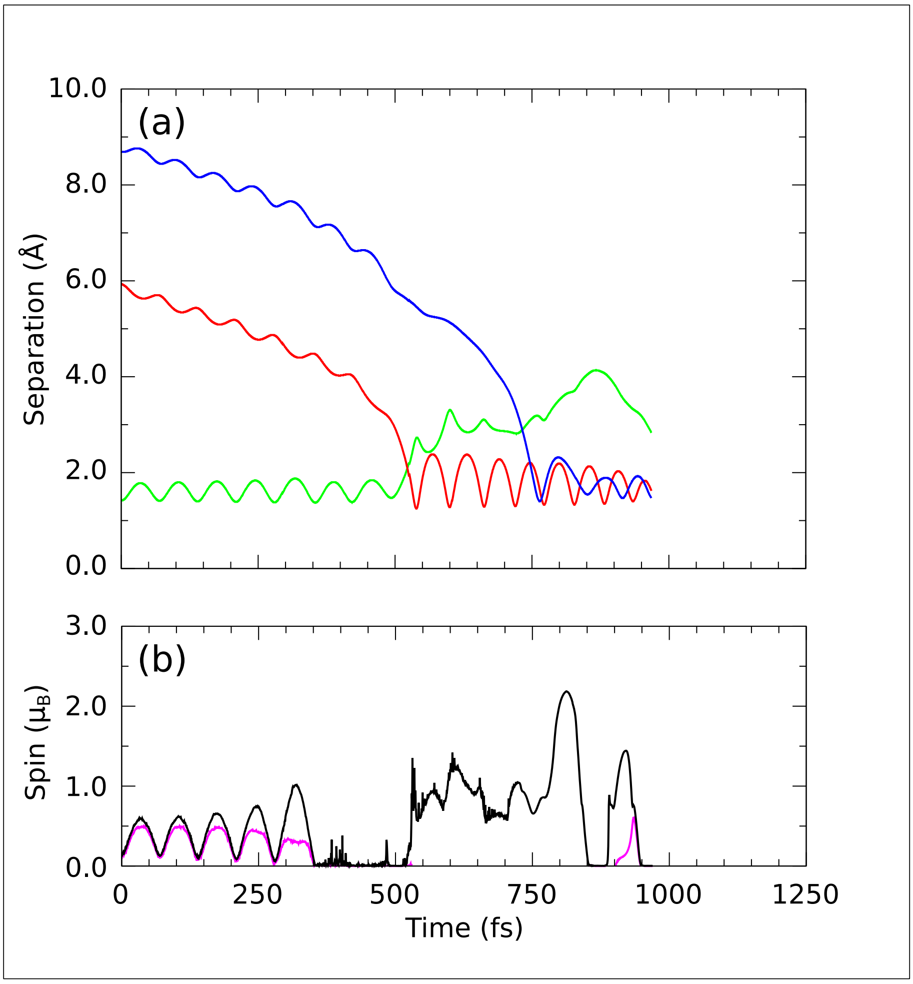

Figure S48: Bond lengths and spin traces during adsorption on the clean Si{001} surface via the E/γ trajectory.

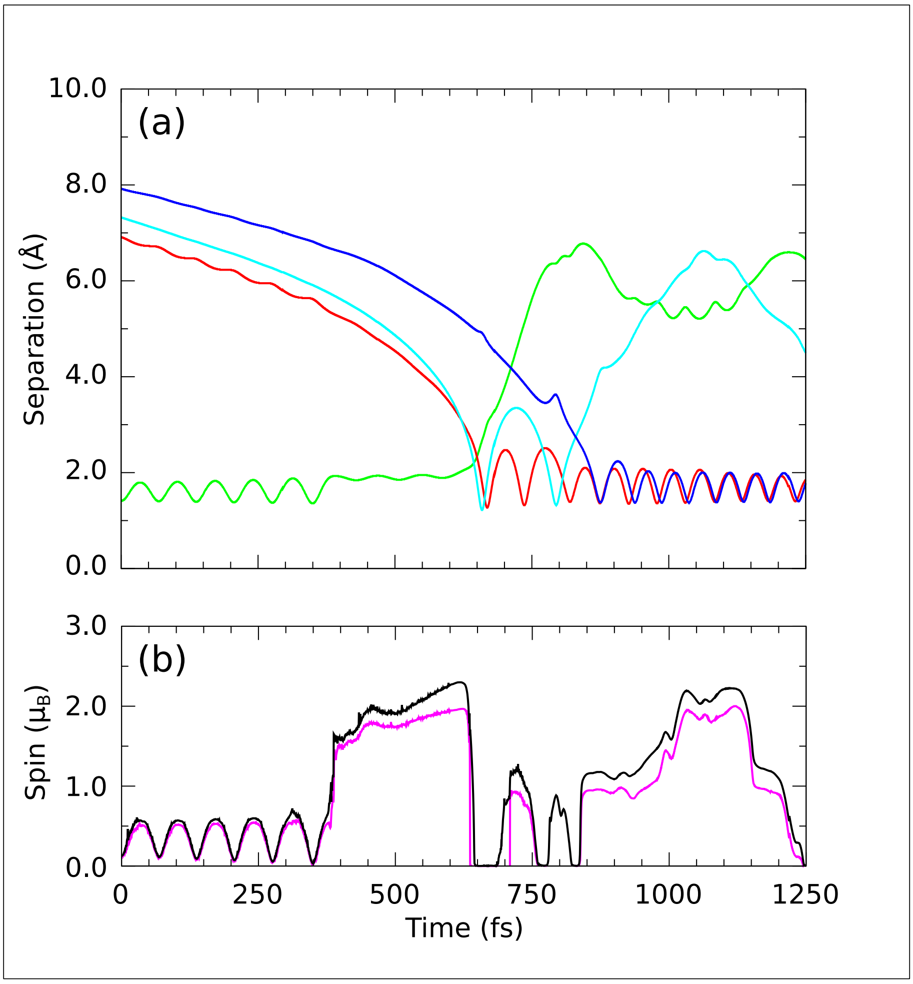

Figure S49: Bond lengths and spin traces during adsorption on the clean Si{001} surface via the F/α trajectory.

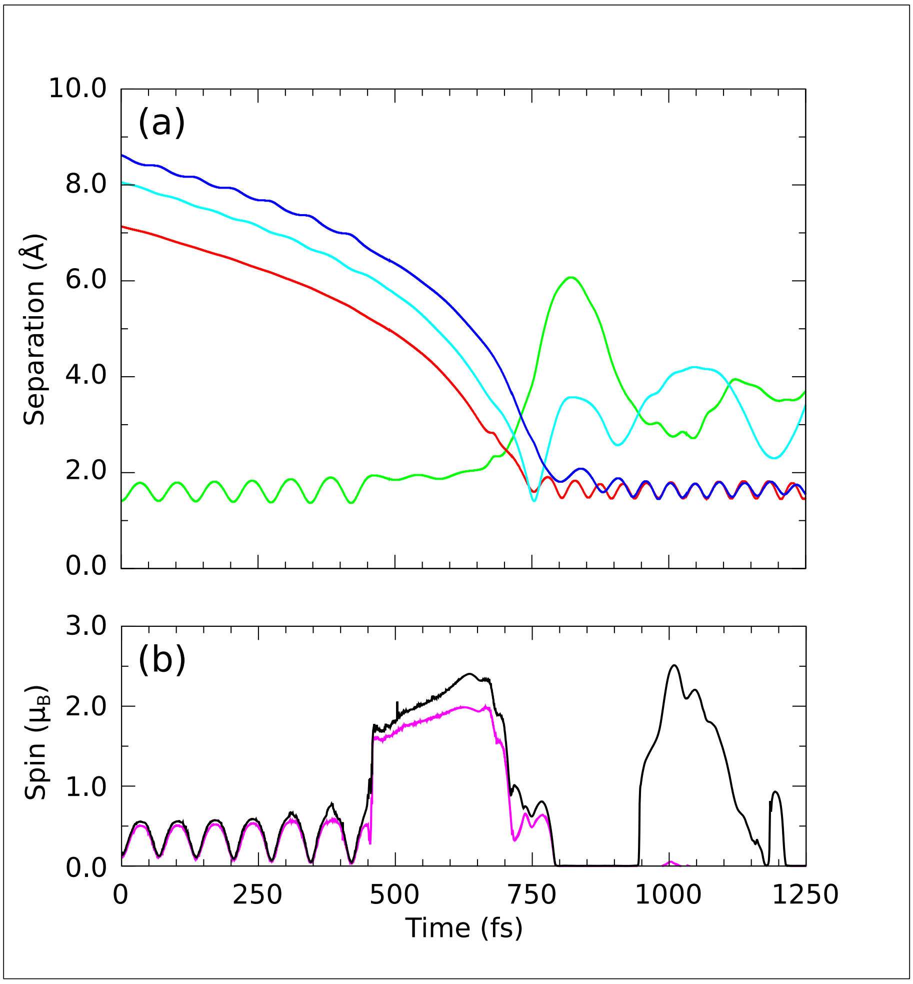

Figure S50: Bond lengths and spin traces during adsorption on the clean Si{001} surface via the F/β trajectory.

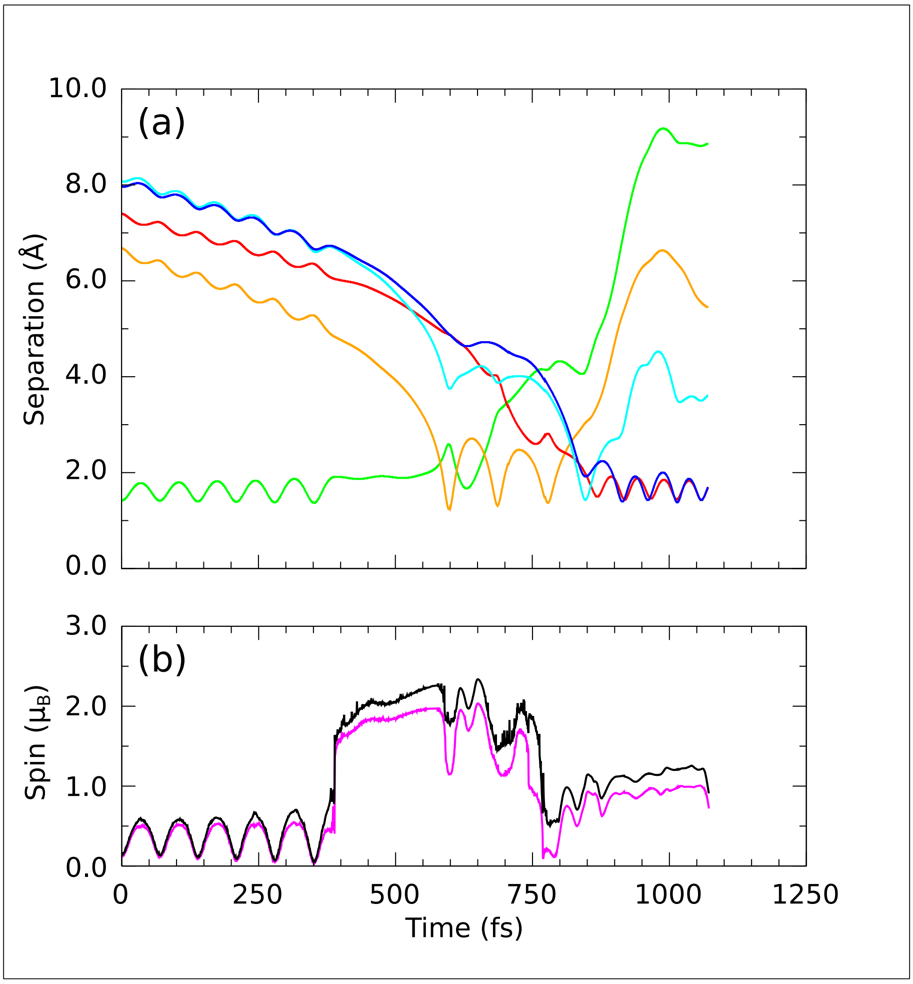

Figure S51: Bond lengths and spin traces during adsorption on the clean Si{001} surface via the F/γ trajectory.

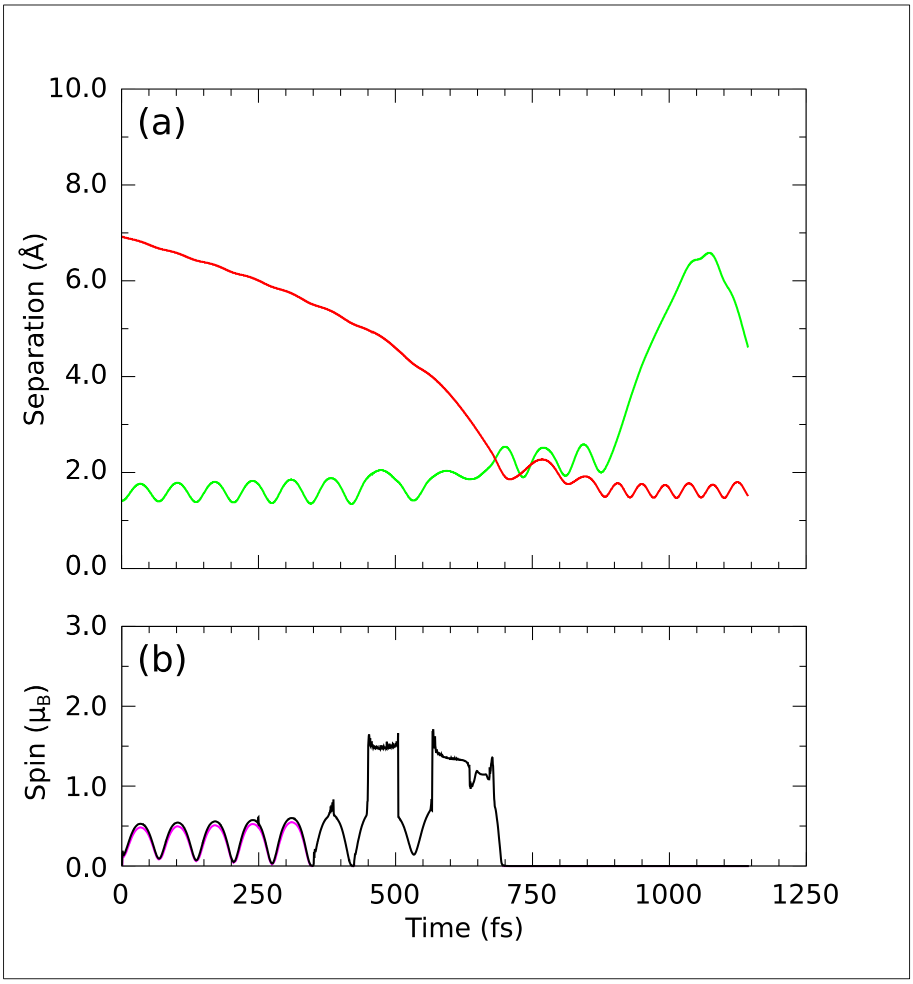

Figure S52: Bond lengths and spin traces during adsorption on the hydrogen-passivated Si{001} surface via the A/α trajectory.

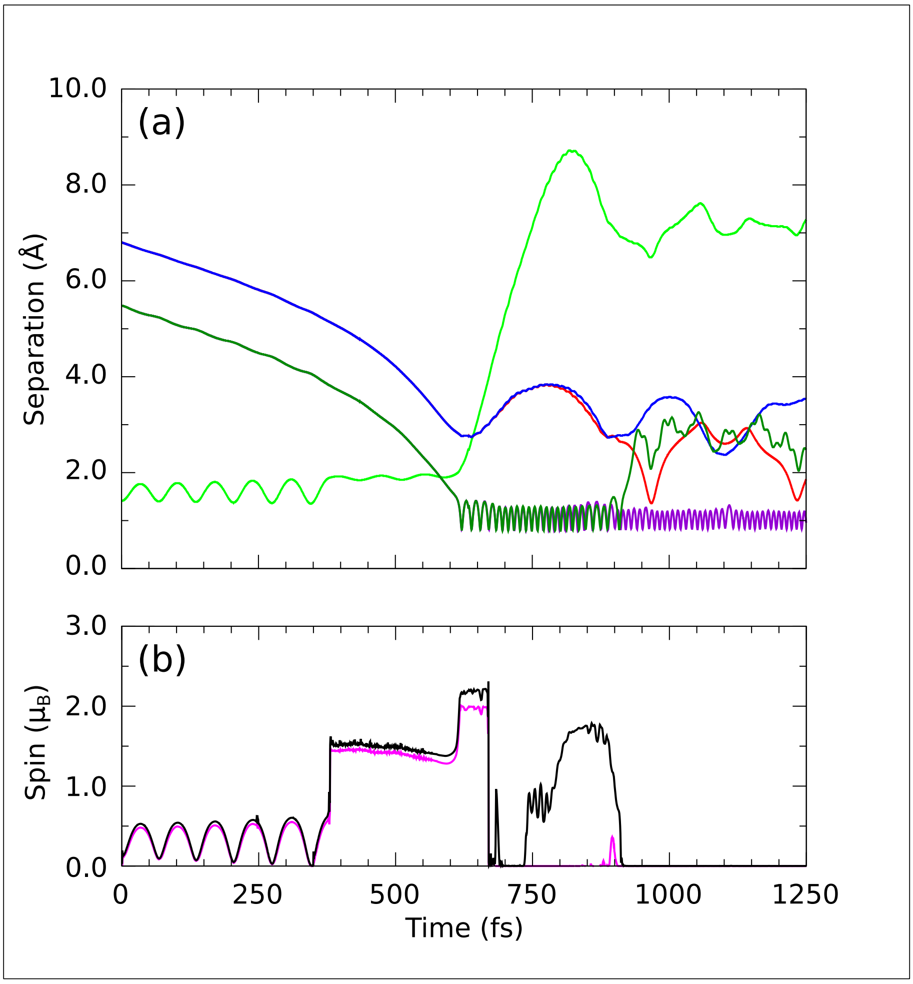

Figure S53: Bond lengths and spin traces during adsorption on the hydrogen-passivated Si{001} surface via the A/β trajectory.

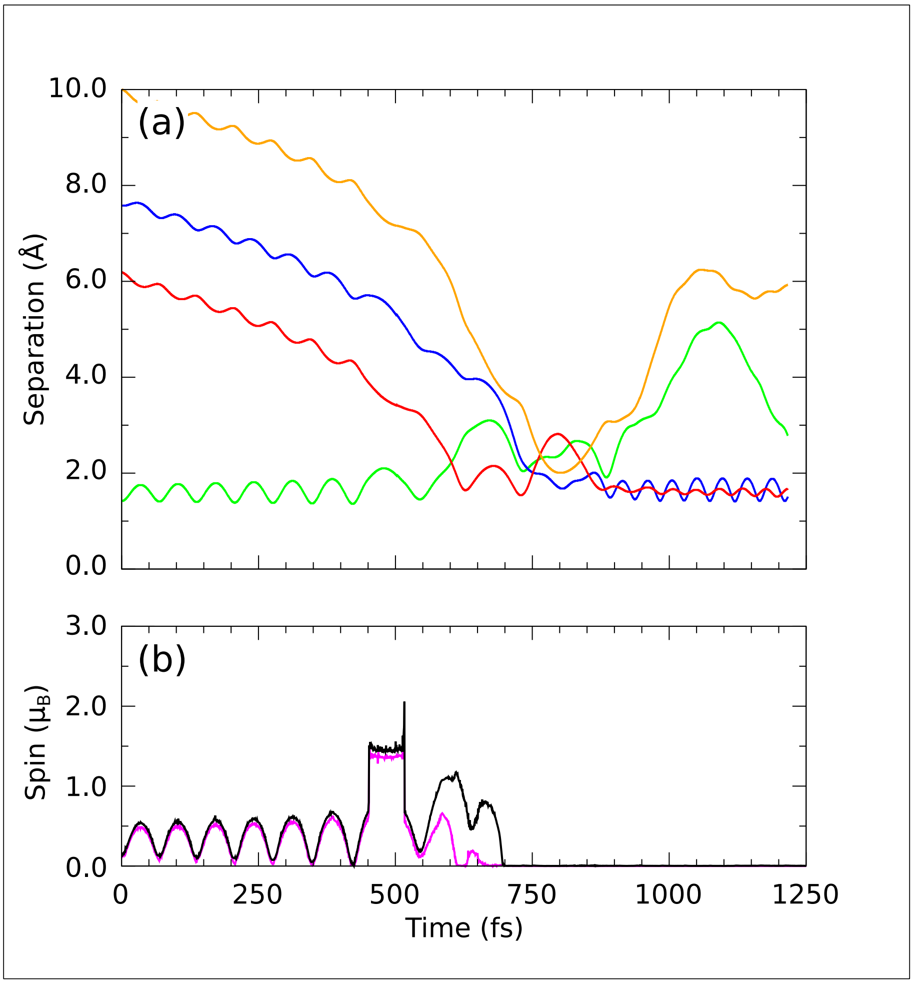

Figure S54: Bond lengths and spin traces during adsorption on the hydrogen-passivated Si{001} surface via the A/γ trajectory.

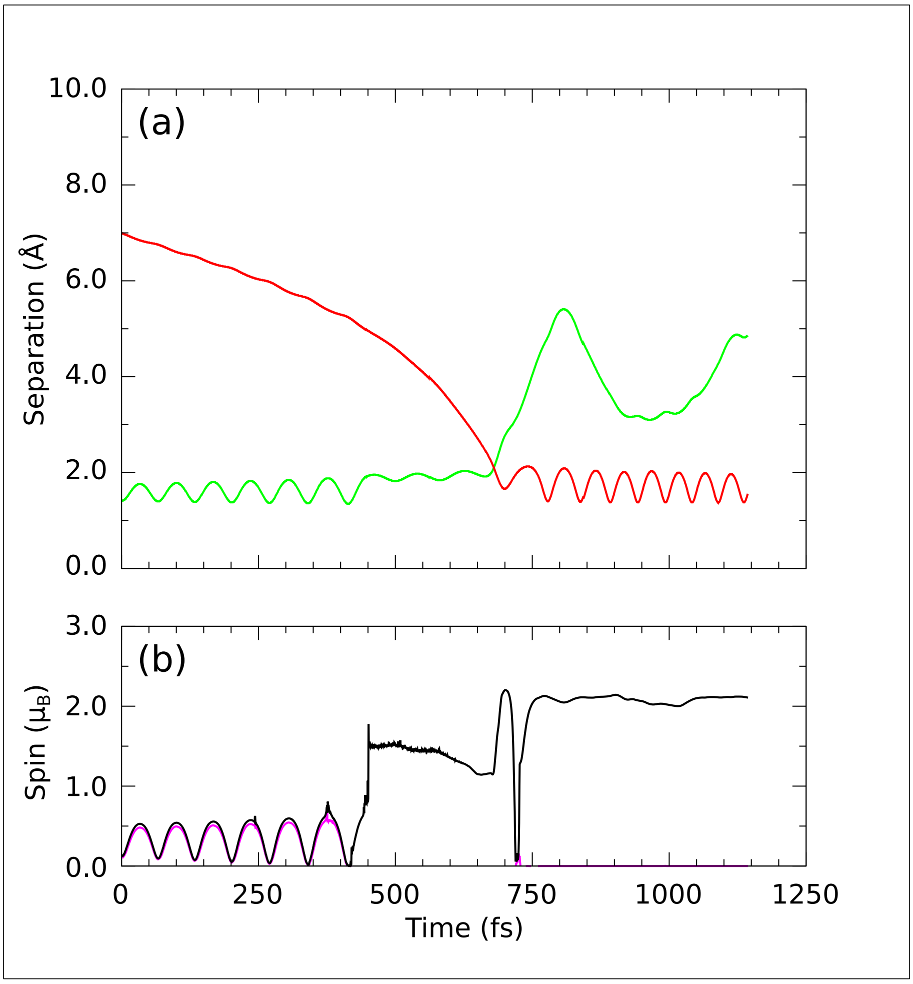

Figure S55: Bond lengths and spin traces during adsorption on the hydrogen-passivated Si{001} surface via the B/α trajectory.

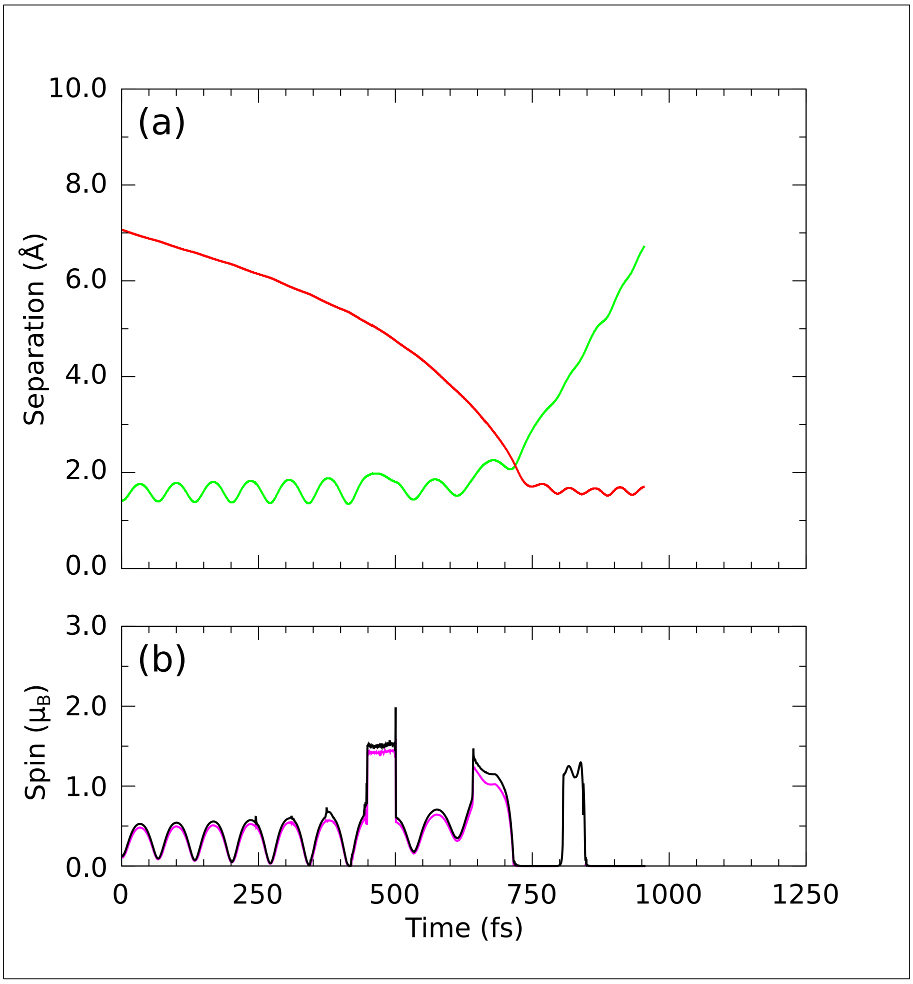

Figure S56: Bond lengths and spin traces during adsorption on the hydrogen-passivated Si{001} surface via the B/β trajectory.

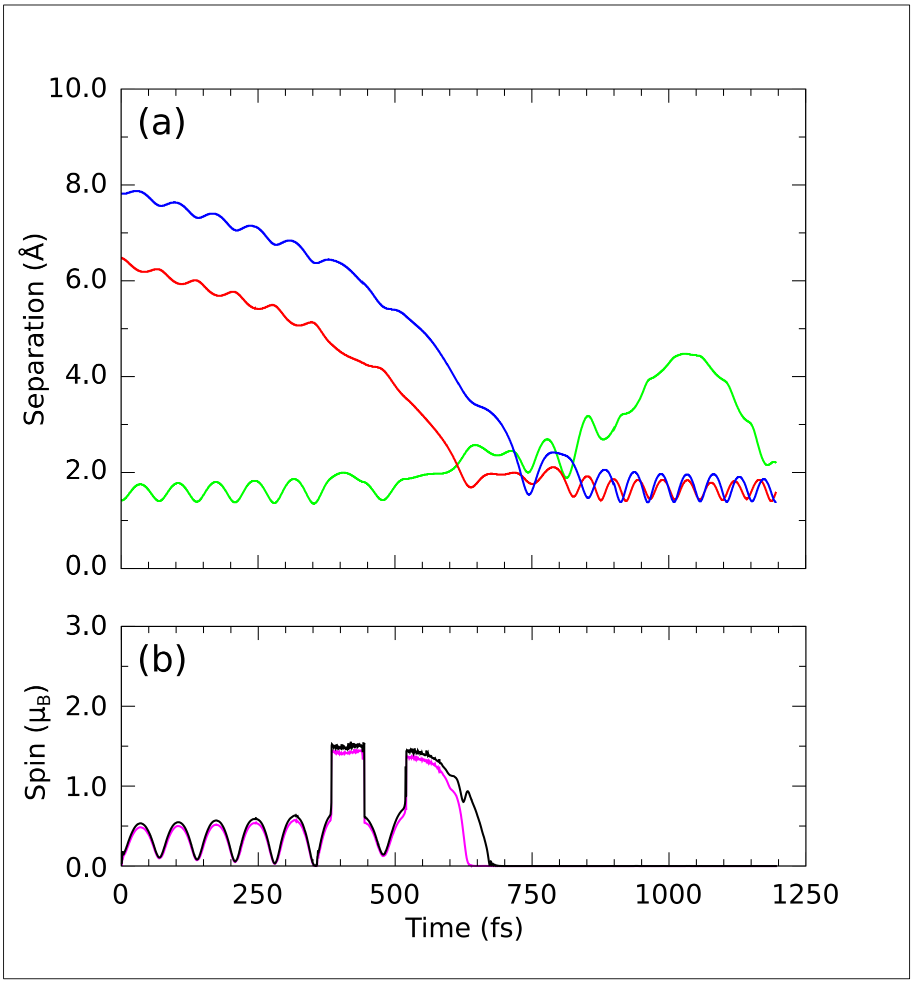

Figure S57: Bond lengths and spin traces during adsorption on the hydrogen-passivated Si{001} surface via the B/γ trajectory.

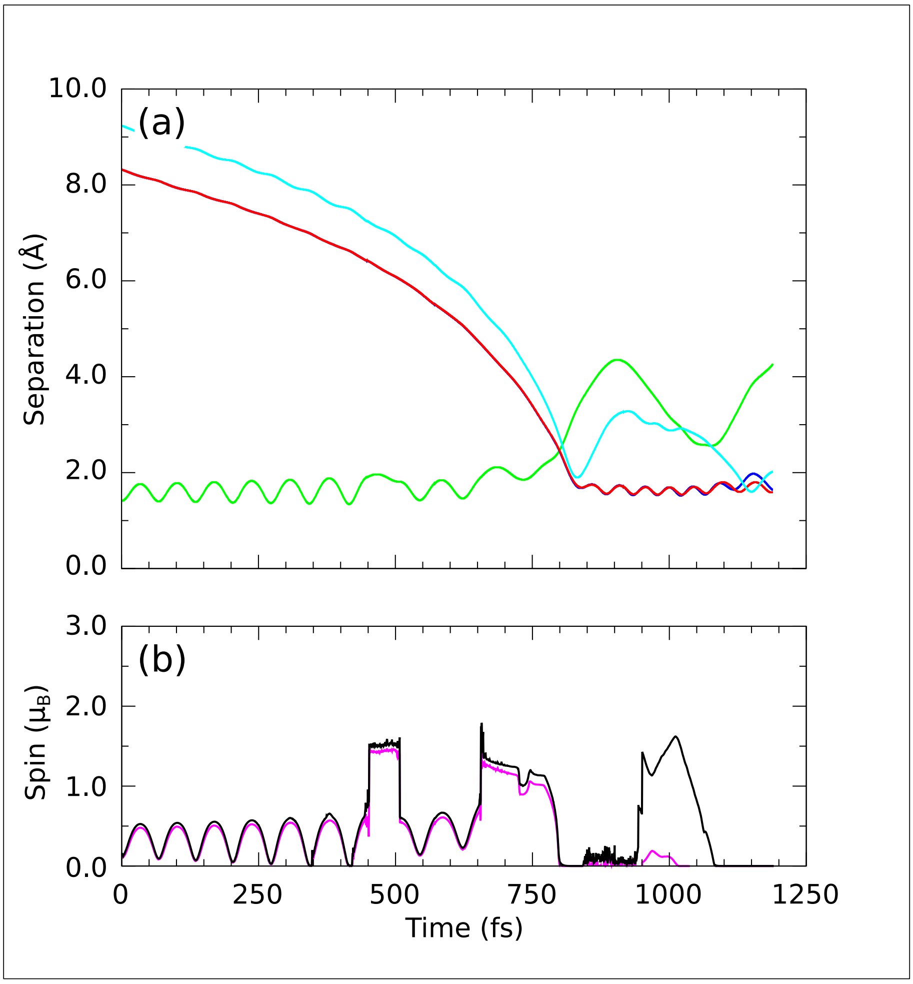

Figure S58: Bond lengths and spin traces during adsorption on the hydrogen-passivated Si{001} surface via the C/α trajectory.

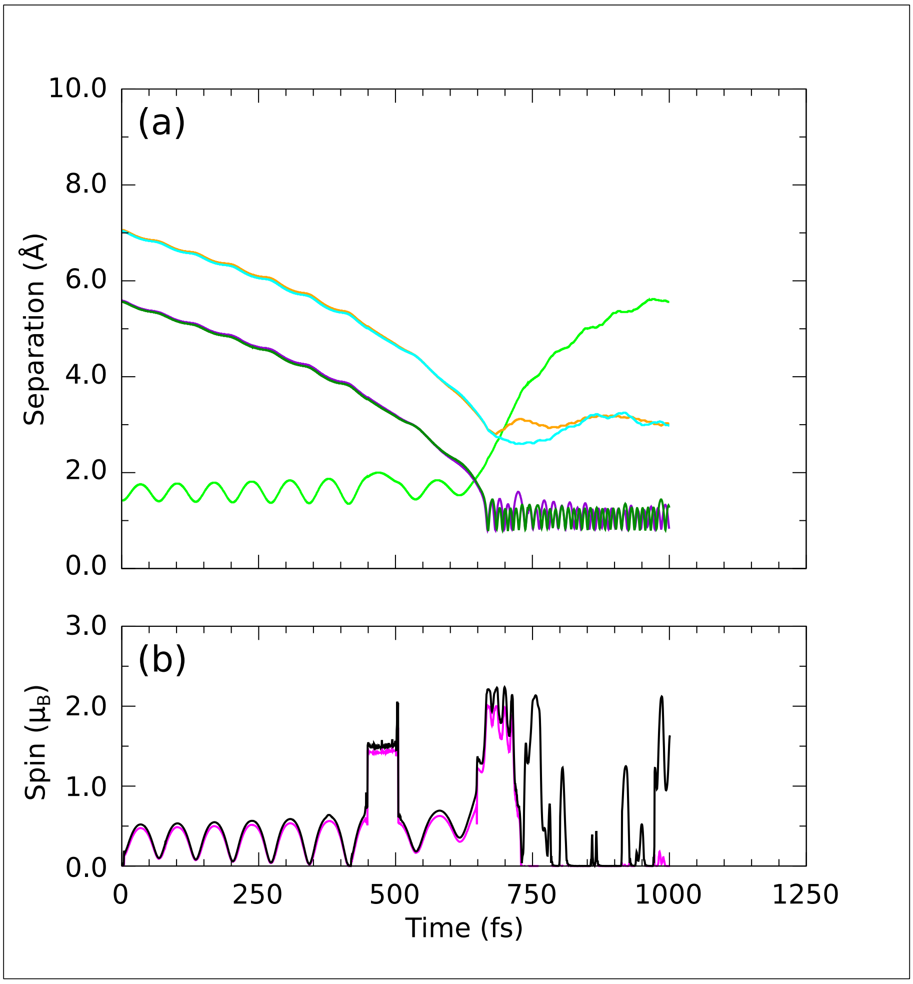

Figure S59: Bond lengths and spin traces during adsorption on the hydrogen-passivated Si{001} surface via the C/β trajectory.

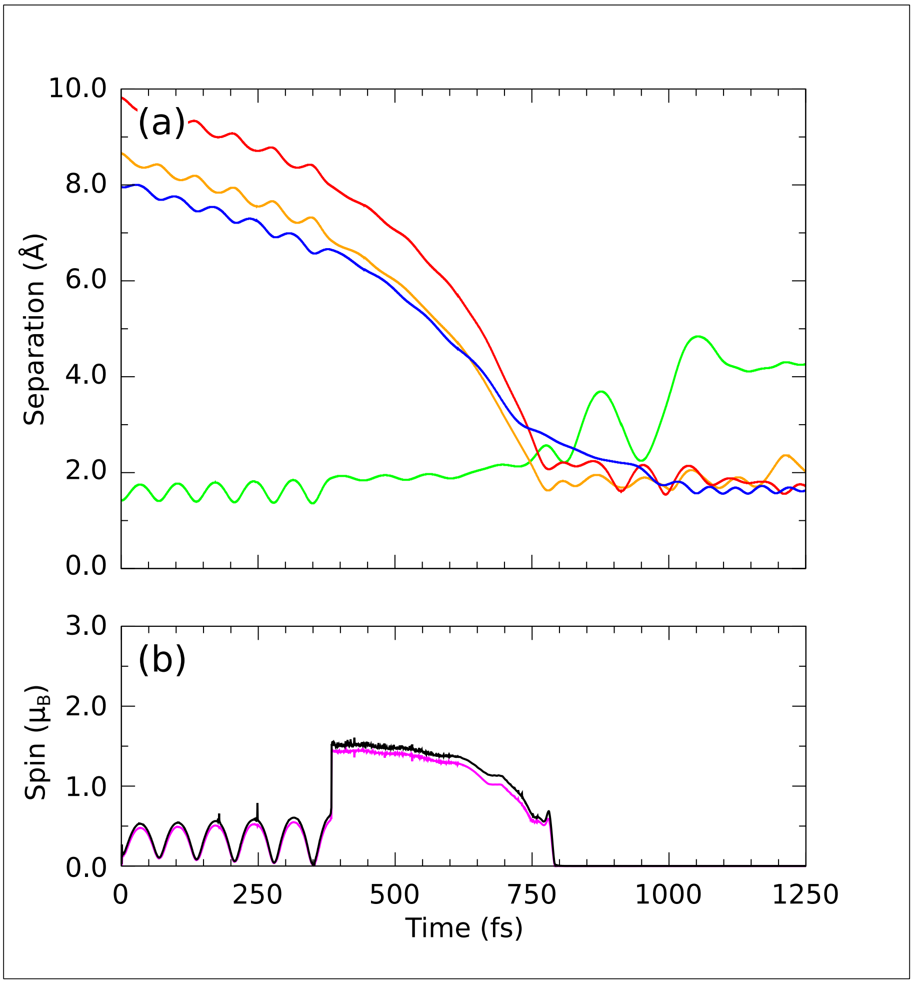

Figure S60: Bond lengths and spin traces during adsorption on the hydrogen-passivated Si{001} surface via the C/γ trajectory.

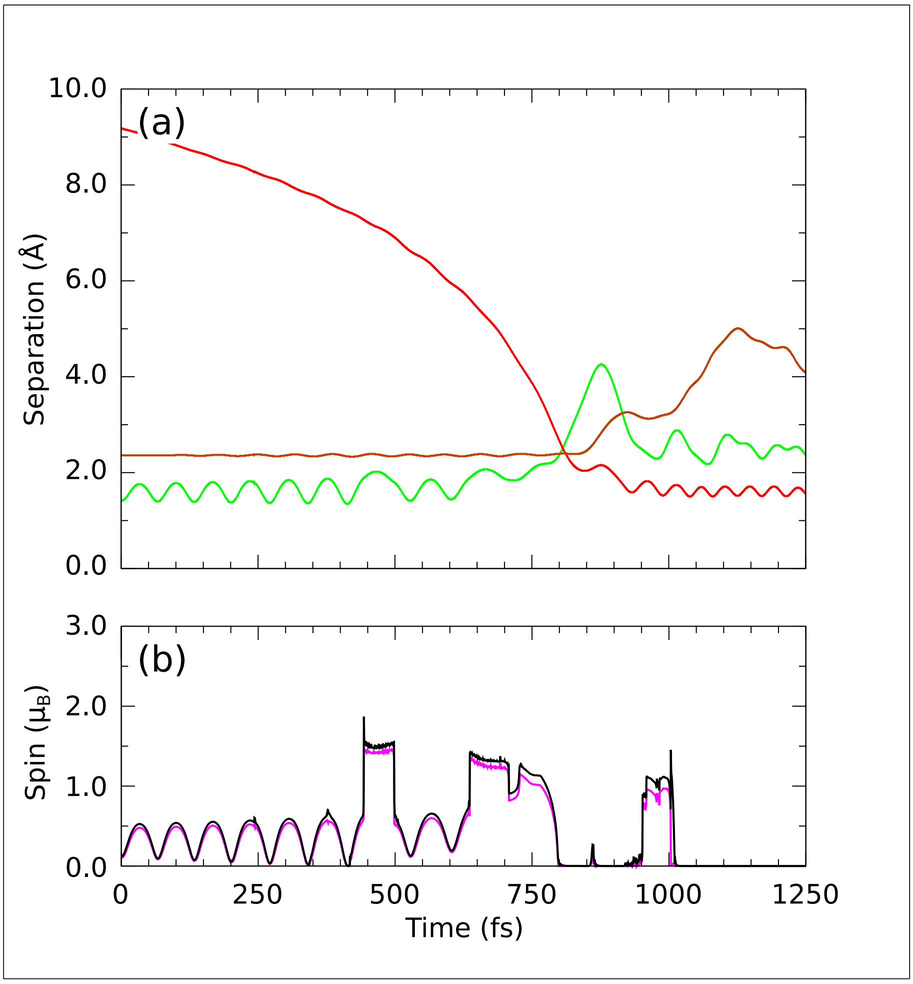

Figure S61: Bond lengths and spin traces during adsorption on the hydrogen-passivated Si{001} surface via the D/α trajectory.

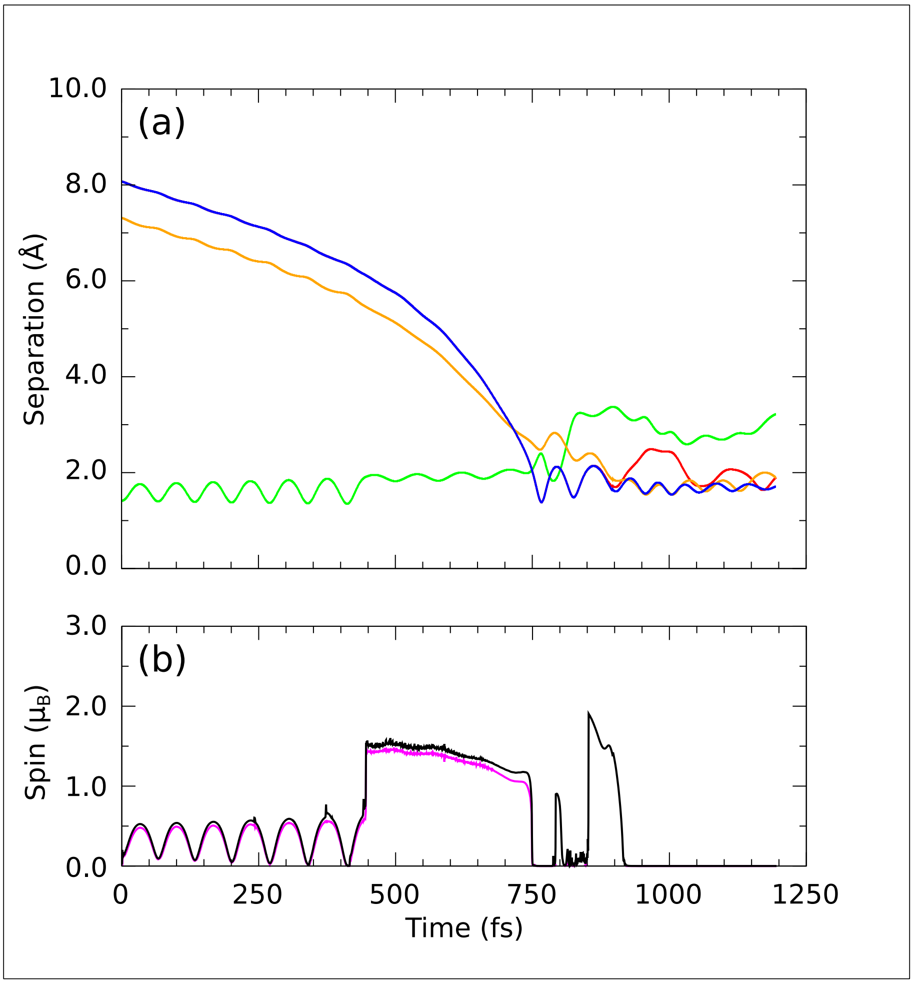

Figure S62: Bond lengths and spin traces during adsorption on the hydrogen-passivated Si{001} surface via the D/β trajectory.

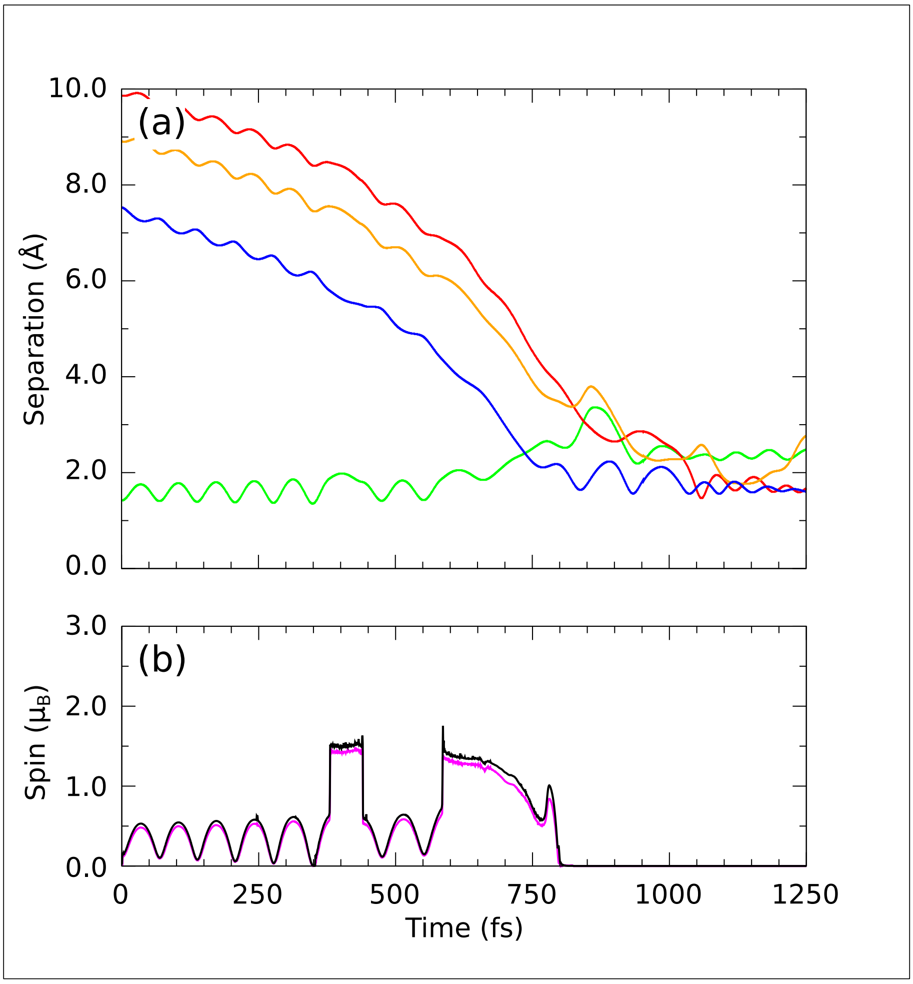

Figure S63: Bond lengths and spin traces during adsorption on the hydrogen-passivated Si{001} surface via the D/γ trajectory.

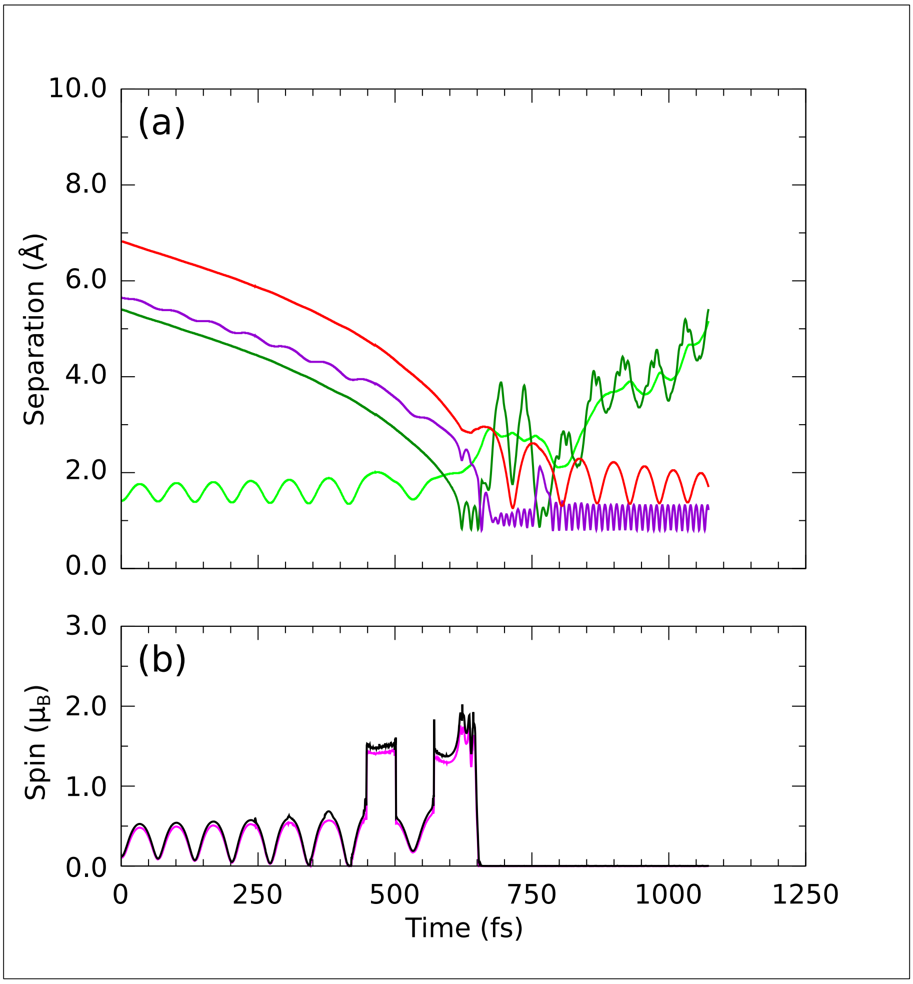

Figure S64: Bond lengths and spin traces during adsorption on the hydrogen-passivated Si{001} surface via the E/α trajectory.

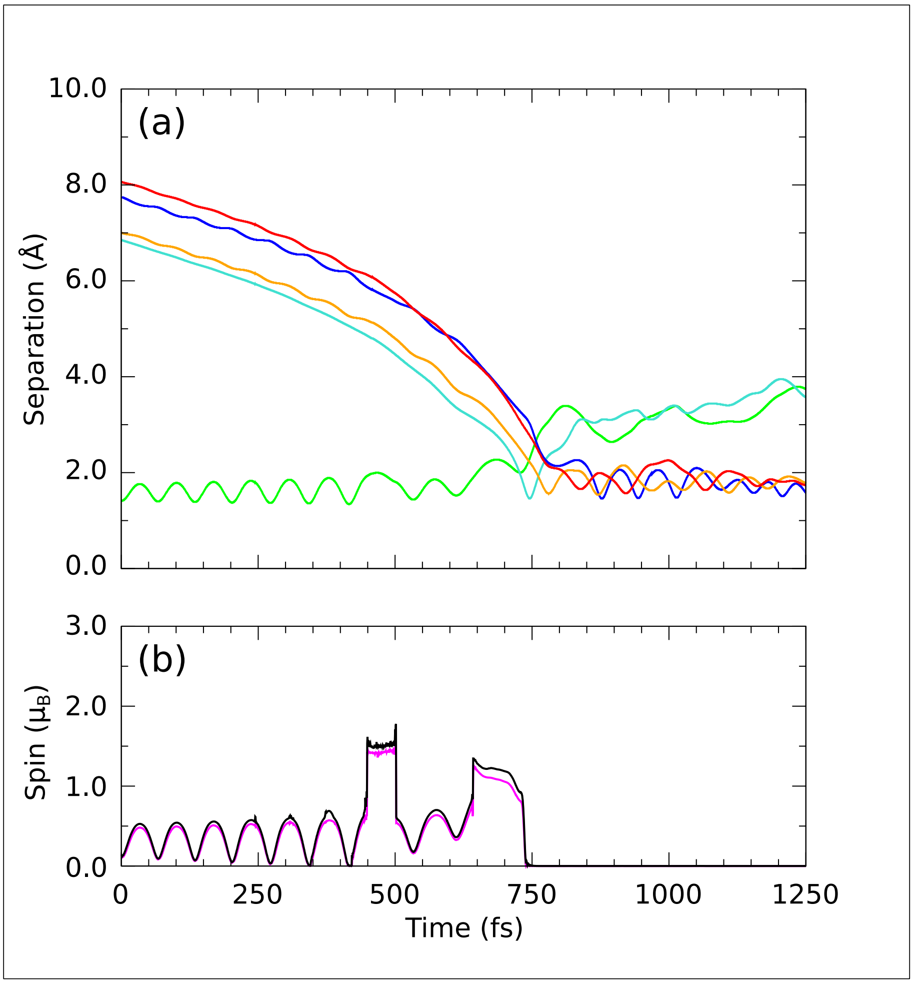

Figure S65: Bond lengths and spin traces during adsorption on the hydrogen-passivated Si{001} surface via the E/β trajectory.

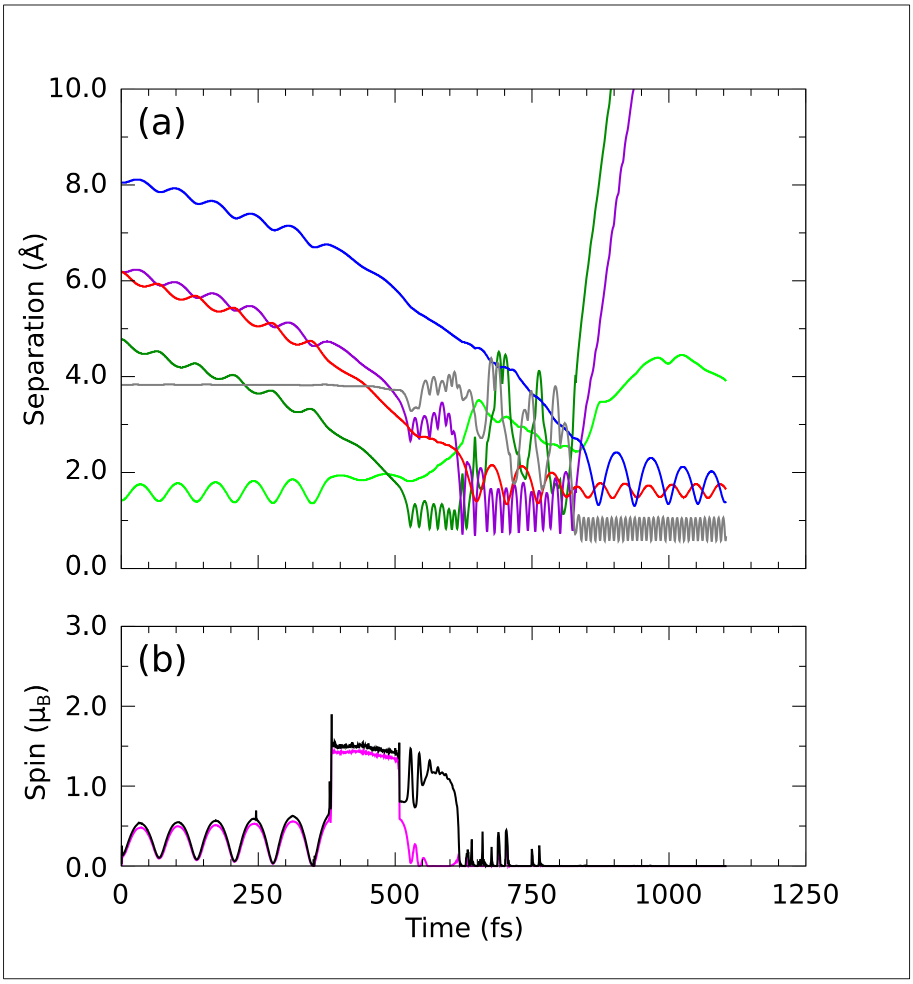

Figure S66: Bond lengths and spin traces during adsorption on the hydrogen-passivated Si{001} surface via the E/γ trajectory.
